# Supplementary material for: Biochemical Analyses of Bioactive Extracts from Plants Native to Lampedusa, Sicily Minor Island
Source: Plants (Basel). 2022 Dec 9;11(24):3447. doi: 10.3390/plants11243447 (PMC9788634; doi:10.3390/plants11243447)
Supplement: Supplementary file 1 [file plants-11-03447-s001.zip › plants-2041410-supplementary.pdf]

Article

Biochemical analyses of bioactive extracts from plants native to Lampedusa, Sicily minor island

Roberta Di Lecce[1], Natacha Mérindol[2], Mayra Galarza Pérez[3], Vahid Karimzadegan[2], Lionel Berthoux[4], Angela Boari[5], Christian Zidorn[3], Maurizio Vurro[5], Giuseppe Surico[6], I. Desgagné-Penix[2] and Antonio Evidente[1,5*]

[1] Department of Chemical Sciences, University of Naples Federico II, Complesso Universitario Monte Sant'Angelo, Napoli, Italy
[2] Département de Chimie, Biochimie et Physique, Université du Québec à Trois-Rivières, Trois-Rivières, QC, Canada
[3] Pharmazeutisches Institut, Abteilung Pharmazeutische Biologie, Christian-Albrechts-Universitat ¨ zu Kiel, Gutenbergstraße 76, 24118, Kiel, Germany
[4] Département de Biologie Médicale, Université du Québec à Trois-Rivières, Trois-Rivières, QC, Canada
[5] Institute of Sciences of Food Production, National Research Council, Via Amendola 122/O, 70125 Bari, Italy
[6] Department of Agriculture, Food, Environment, and Forestry (DAGRI), Section of Agricultural Microbiology, Plant Pathology and Enthomology, University of Florence, Italy
* Correspondence: e-evidente@unina.it

Supplementary Materials

PLANTS

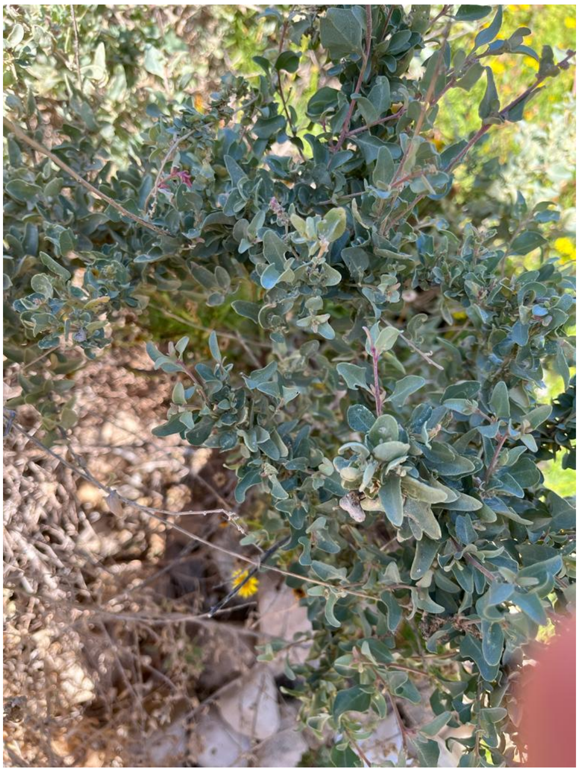

Atriplex halimus L. (*Ap*)

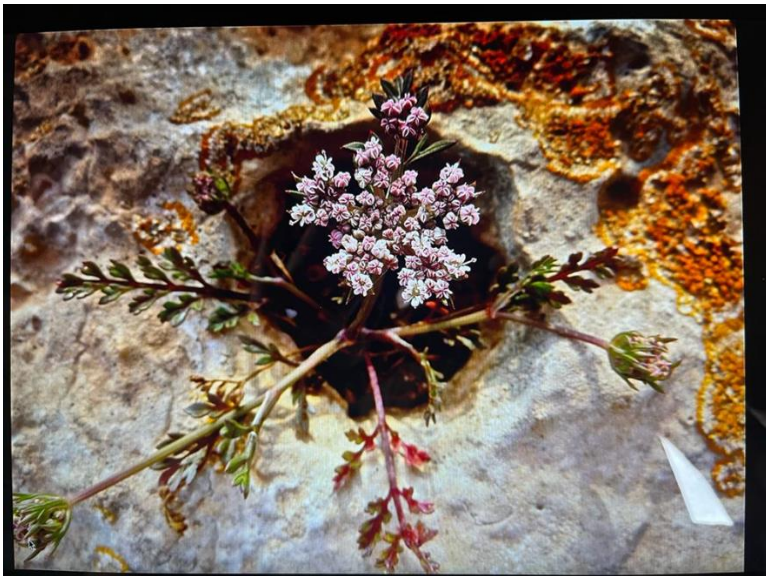

Daucus lopadusanus Tineo (*Dl*)

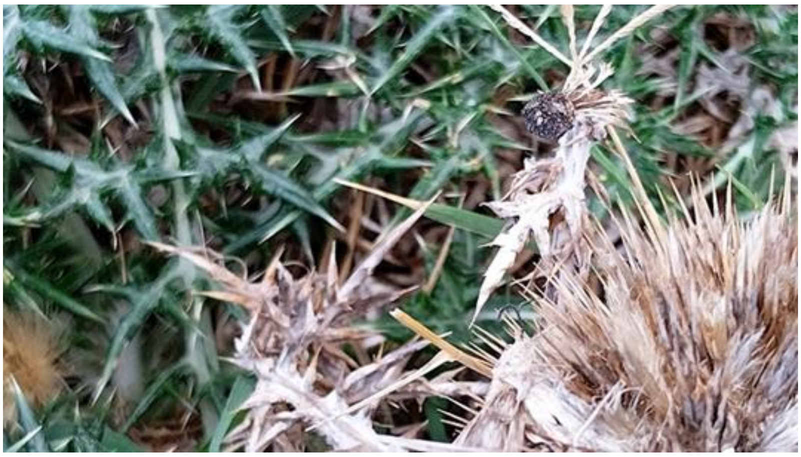

Echinops spinosus Fiori *(Es)*

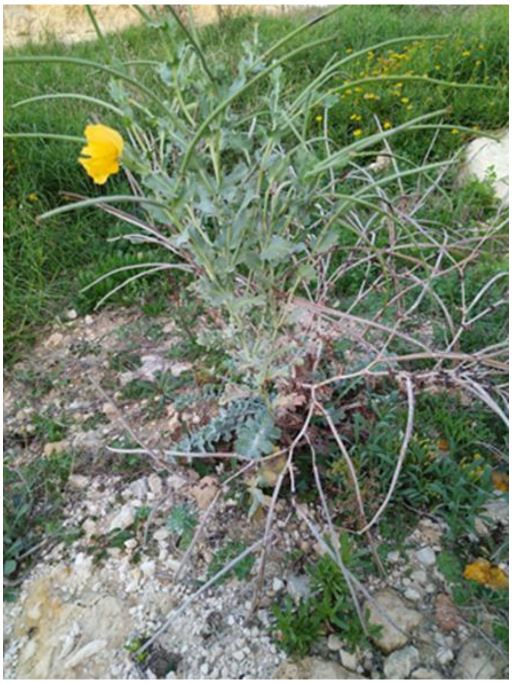

Glaucium flavum Crantz *(Gf)*

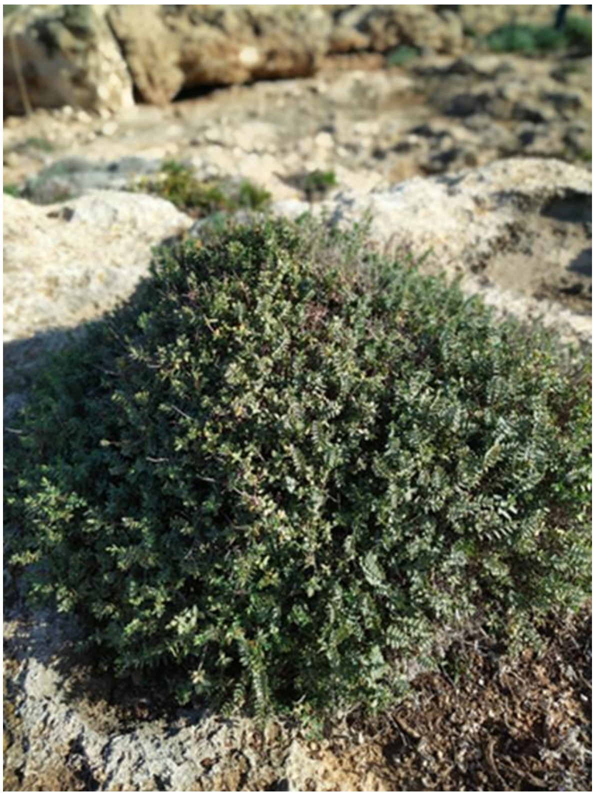

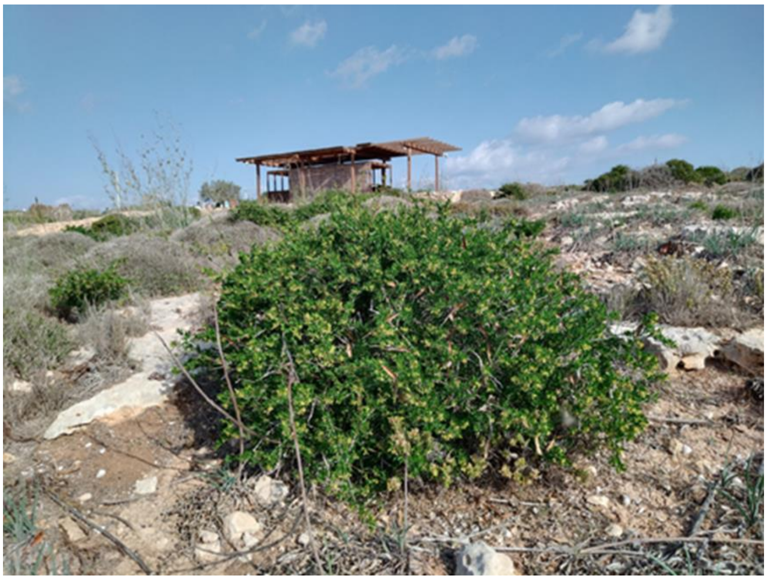

***Hypericum aegypticum* L. (*Ha*)** ***Periploca angustifolia* Labill (*Pa*)**

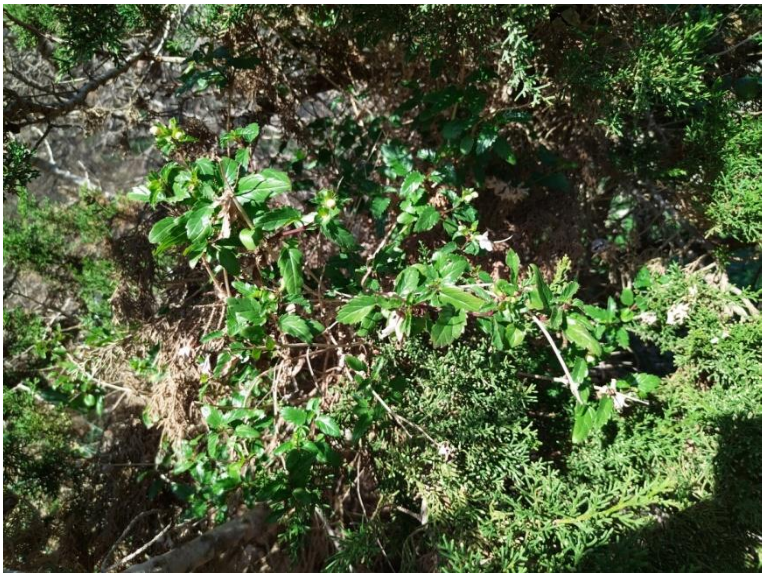

***Prasium majus* L. (*Pm*)**

References

1. Sommier S. *Le isole Pelagie – Lampedusa, Linosa e Lampione - e la loro flora*;Stabilimento Pellas, Luigi Chiti Successore: Firenze, Italia, 1908.
2. Di Martino, A. Flora e vegetazione. In:Zavattari e Coll. *Biogeografia delle isole Pelagie*, Zavattari e Coll. Eds.; Rendiconti Accademia Nazionale dei XL: Roma, Italia, 1958; serie IV, vol. XI, pp. 163-261

–Corti, C.; Lo Cascio, P.; Massetti, M.; Corti C.; Pasta S. *Storia naturale delle isole Pelagie*,; Società Editrice L'Epos: Palermo, Italia,2002; pp. 151-192.

Daucus lopadusanus

List of Figures

H_2O phase

Figure S1a. Base Peak Chromatogram (BPC) in the positive ionization mode of water extract of *Daucus lopadusanus*

Figure S1b. Base Peak Chromatogram (BPC) in the negative ionization mode of water extract of *Daucus lopadusanus*.

Figure S1c. PDA chromatogram of water extracts of *Daucus lopadusanus* at 245 nm.

Figure S1d. PDA chromatogram of water extracts of *Daucus lopadusanus* at 360 nm.

Hexane extract

Figure S2a. Base Peak Chromatogram (BPC) in positive mode of *n*-hexane extract of *Daucus lopadusanus*

Figure S2b. Base Peak Chromatogram (BPC) in negative mode of *n*-hexane extract of *Daucus lopadusanus.*

Figure S2c. PDA chromatogram of *n*-hexane extracts of *Daucus lopadu*sanus at 245 nm.

Figure S2d. PDA chromatogram of *n*-hexane extracts of *Daucus lopadusanus* at 360 nm.

CH2Cl2 extract

Figure S3a. Base Peak Chromatogram (BPC) in positive mode of dichloromethane extract of *Daucus lopadusanus*

Figure S3b. Base Peak Chromatogram (BPC) in negative mode of dichloromethane extract of *Daucus lopadusanus*.

Figure S3c. PDA chromatogram of dichloromethane extracts of *Daucus lopadusanus* at 245 nm.

Figure S3d. PDA chromatogram of dichloromethane of *Daucus lopadusanus* at 360 nm.

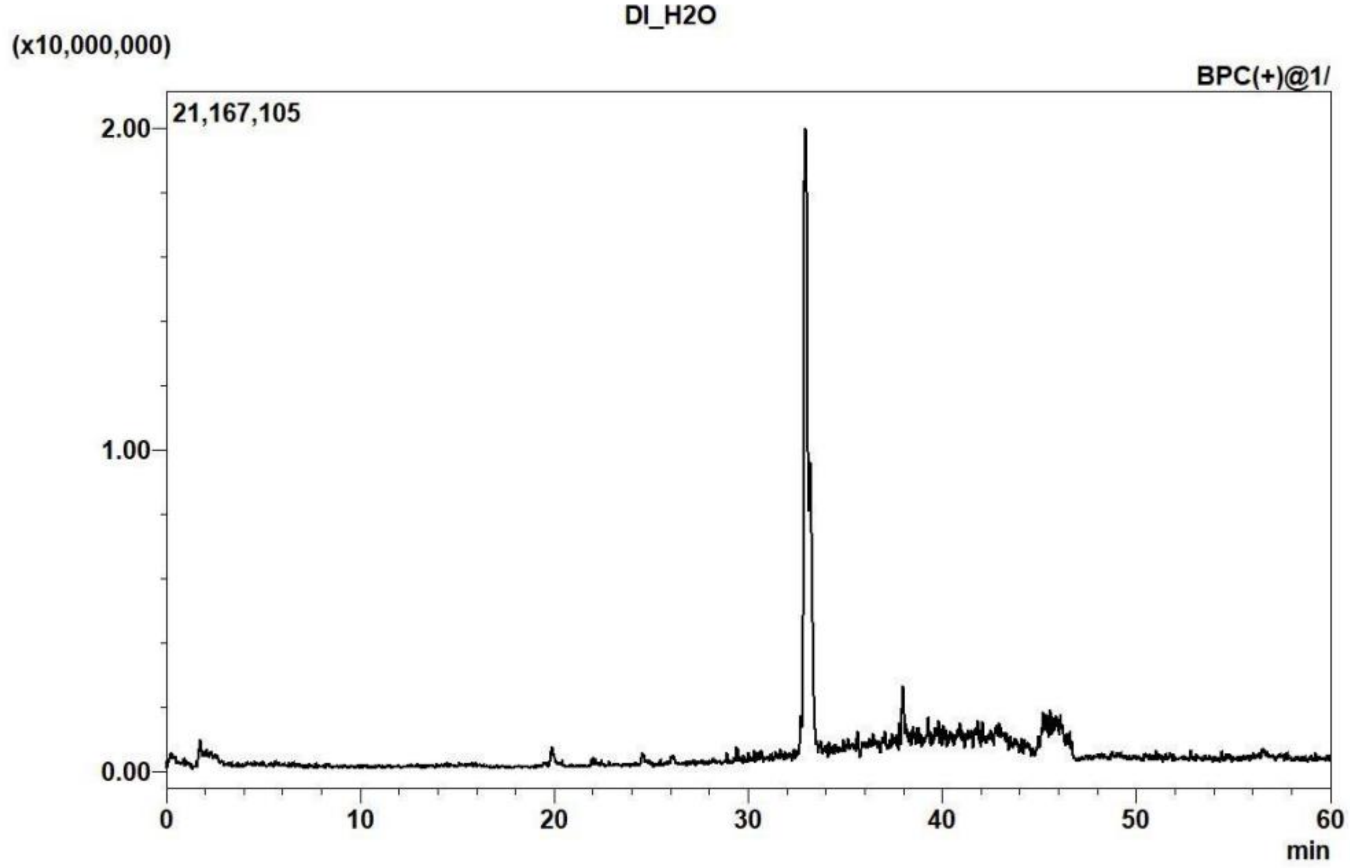
DI_H2O
(x10,000,000)
BPC(+)@1/
21,167,105
2.00
1.00
0.00
0
10
20
30
40
50
60
min

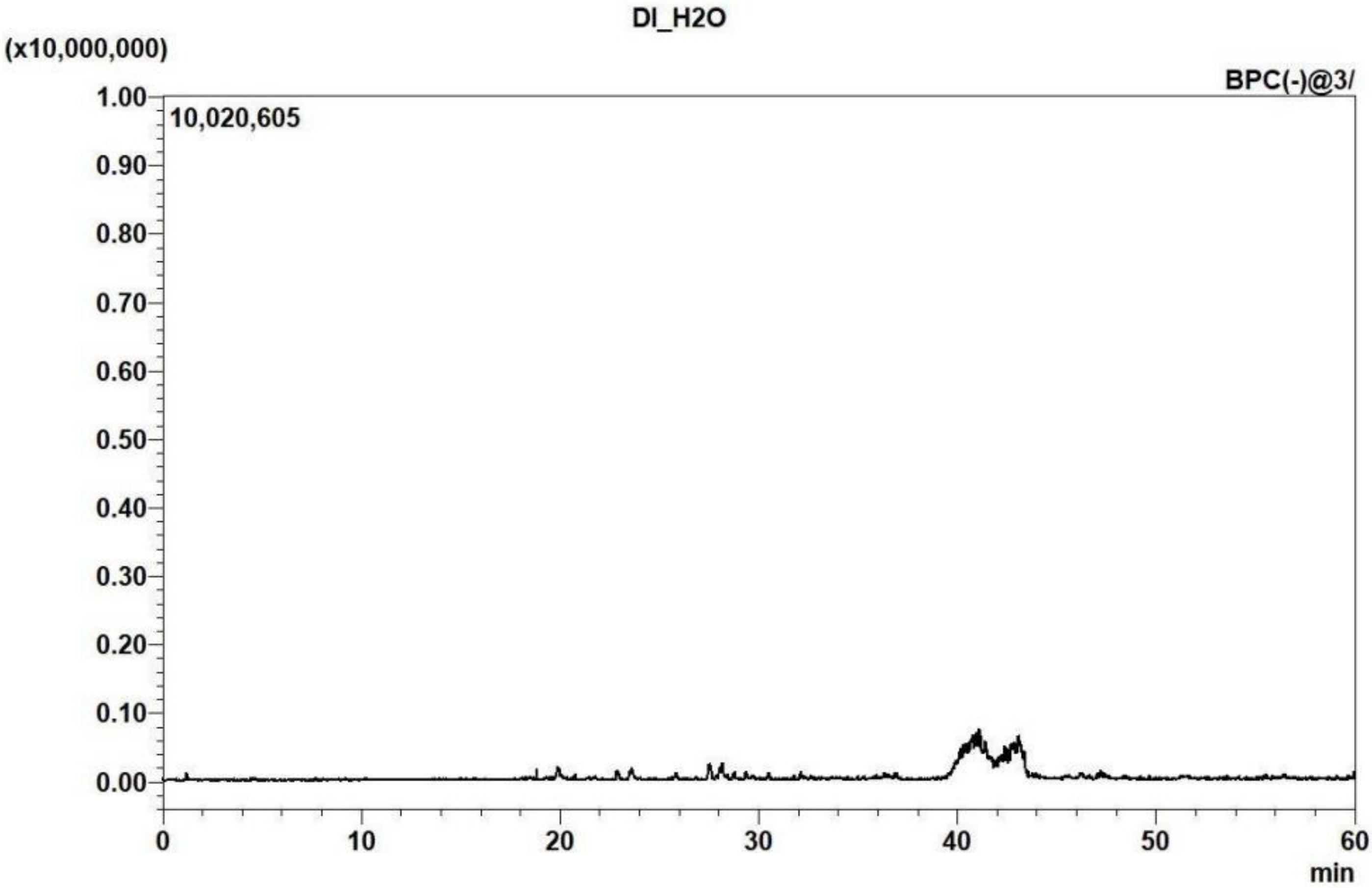
DI_H2O
(x10,000,000)
BPC(-)@3/
10,020,605
1.00
0.90
0.80
0.70
0.60
0.50
0.40
0.30
0.20
0.10
0.00
0
10
20
30
40
50
60
min

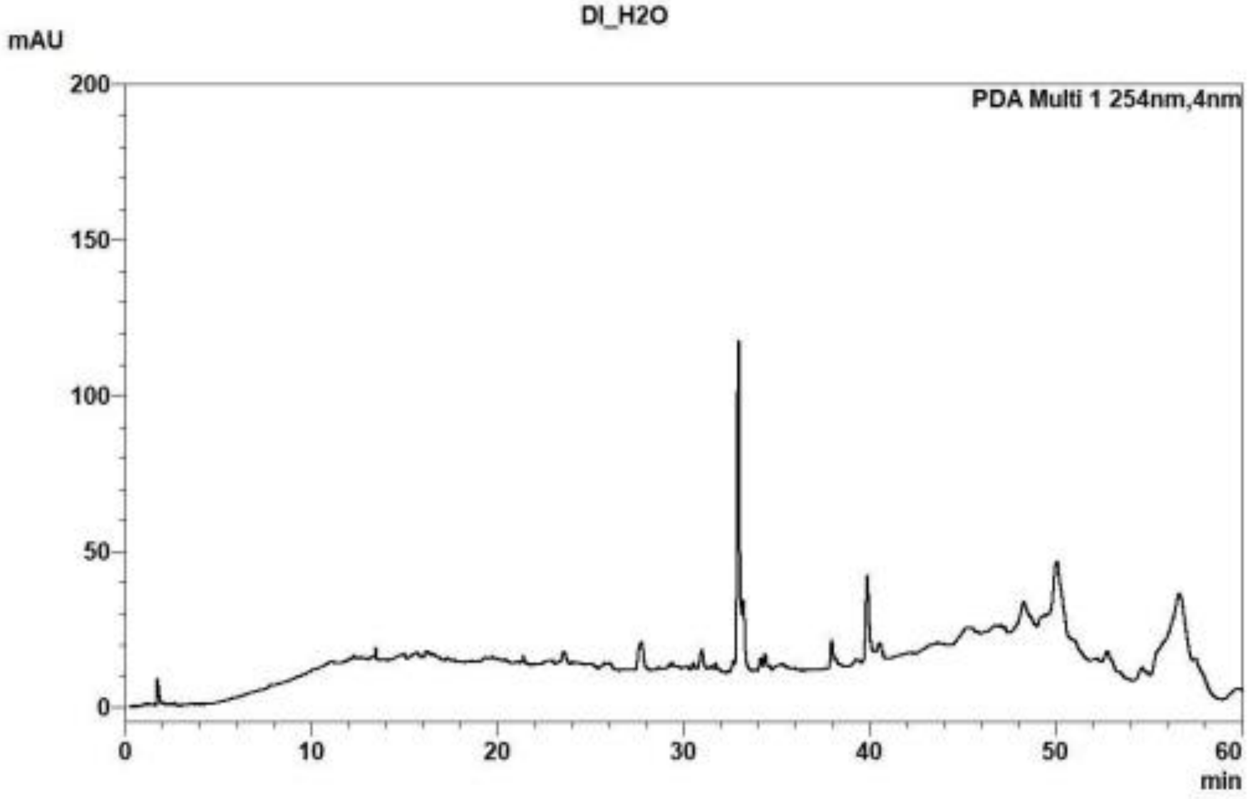
DI_H2O
mAU
PDA Multi 1 254nm,4nm
200
150
100
50
0
0
10
20
30
40
50
60
min

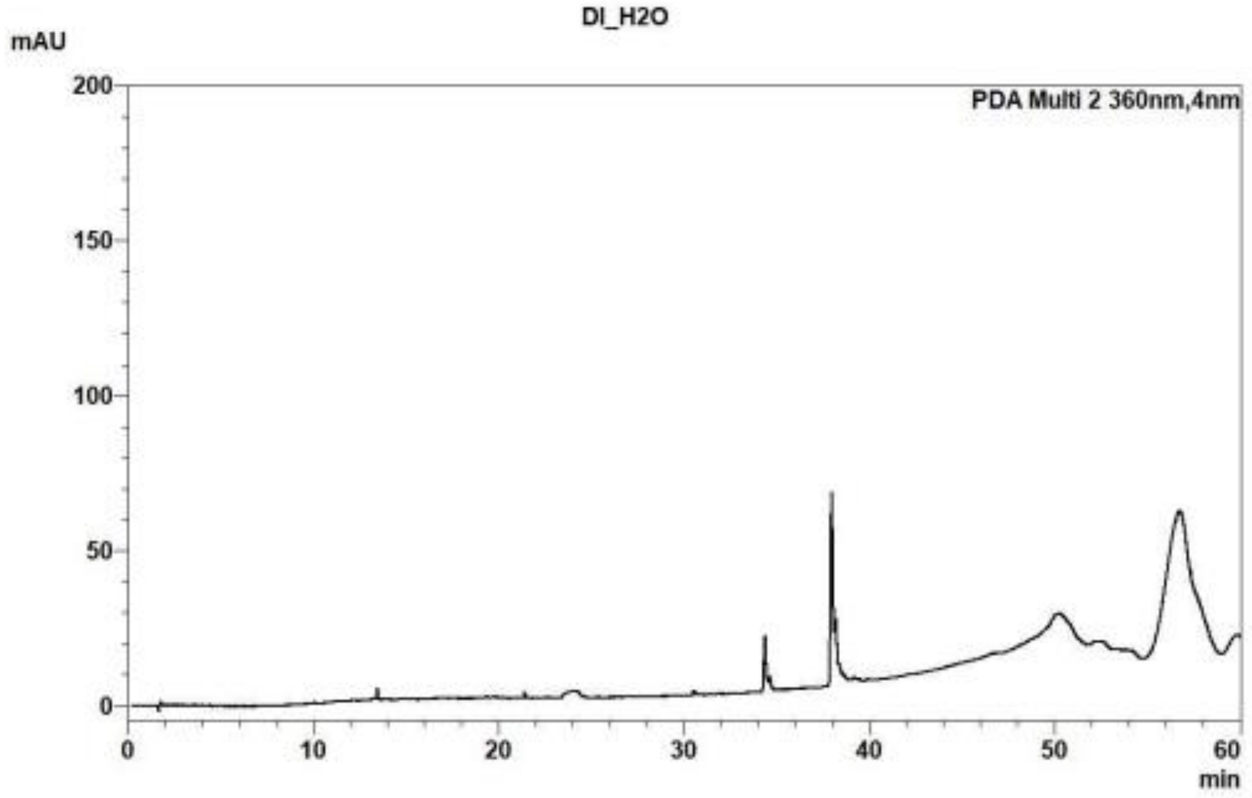
DI_H2O
mAU
PDA Multi 2 360nm,4nm
200
150
100
50
0
0
10
20
30
40
50
60
min

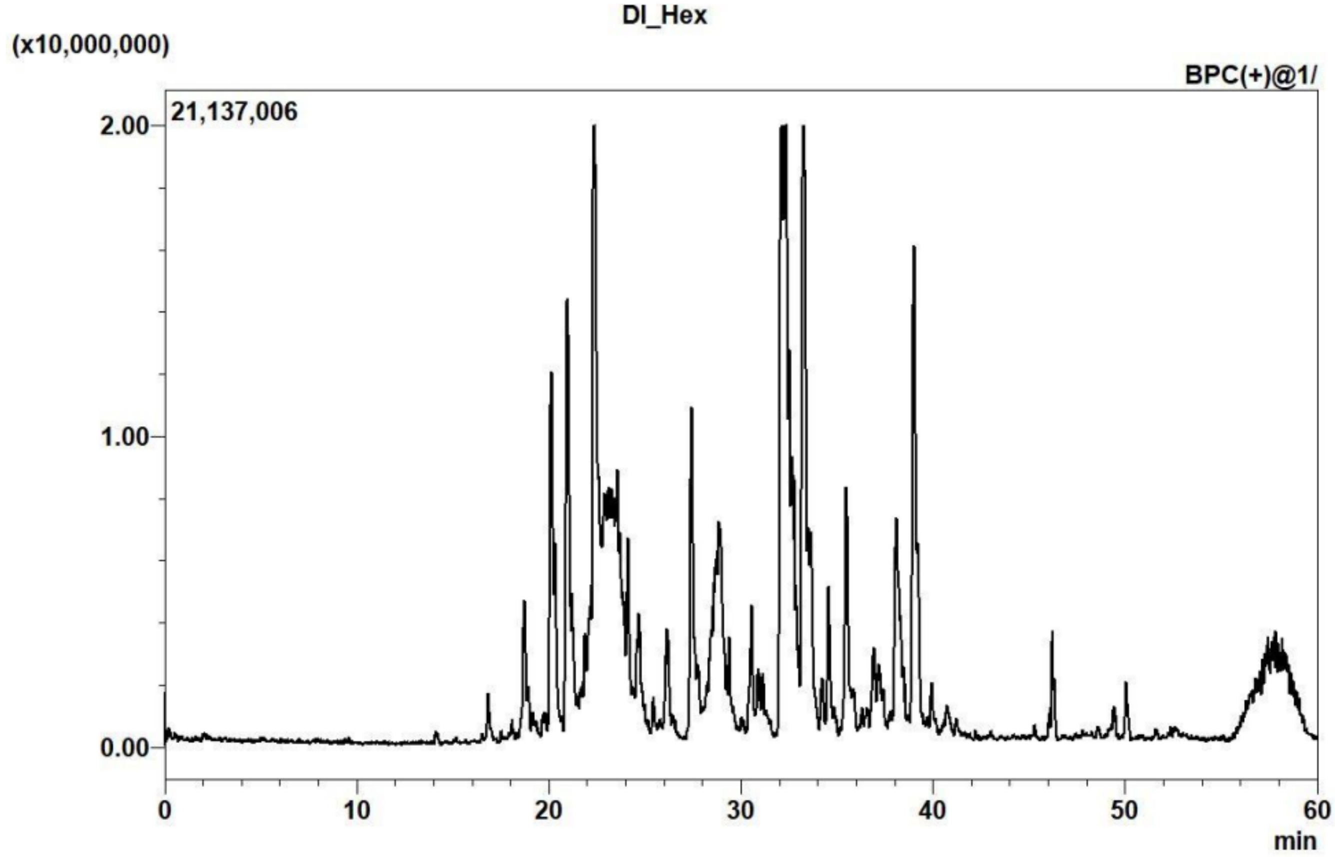

DI_Hex
(x10,000,000)
BPC(+)@1/
21,137,006
2.00
1.00
0.00
0
10
20
30
40
50
60
min

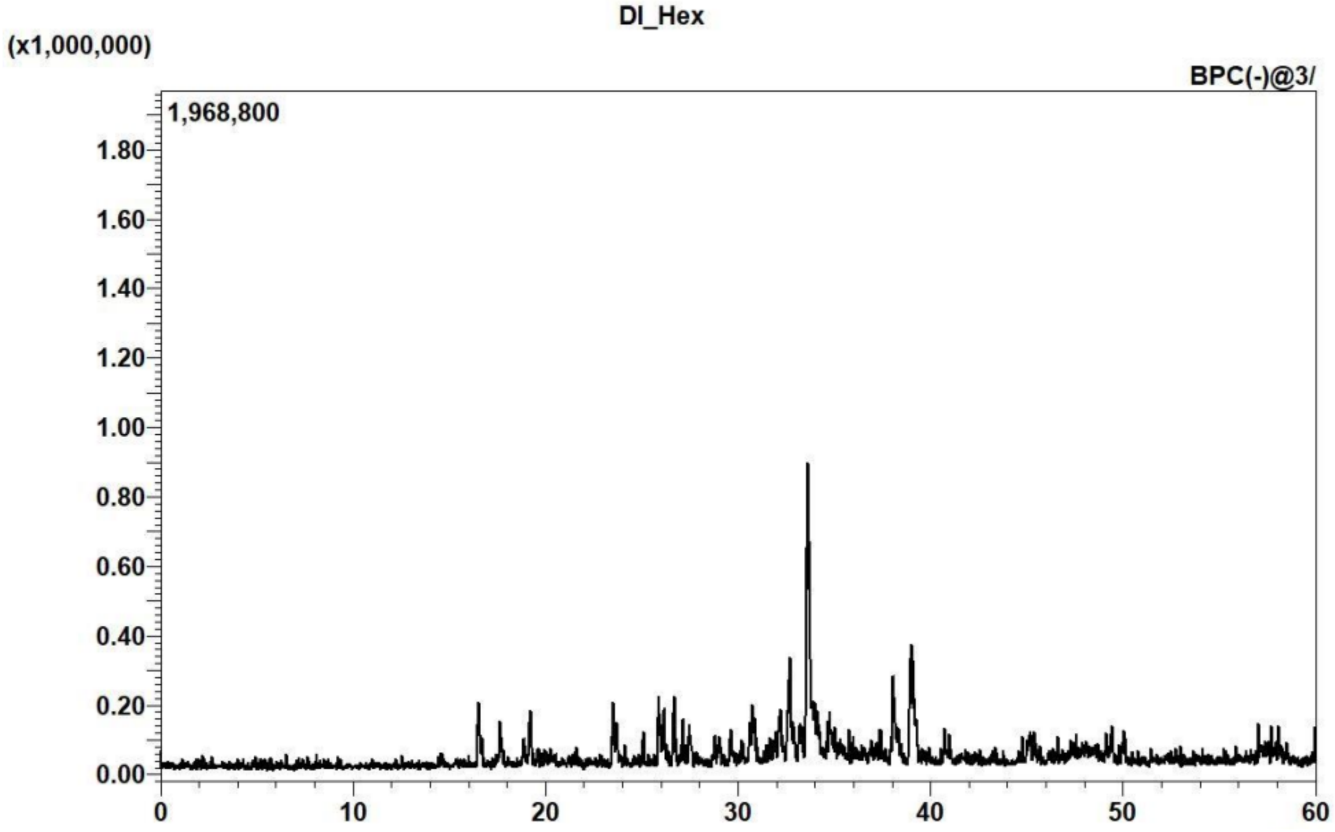

DI_Hex
(x1,000,000)
BPC(-)@3/
1,968,800
1.80
1.60
1.40
1.20
1.00
0.80
0.60
0.40
0.20
0.00
0
10
20
30
40
50
60

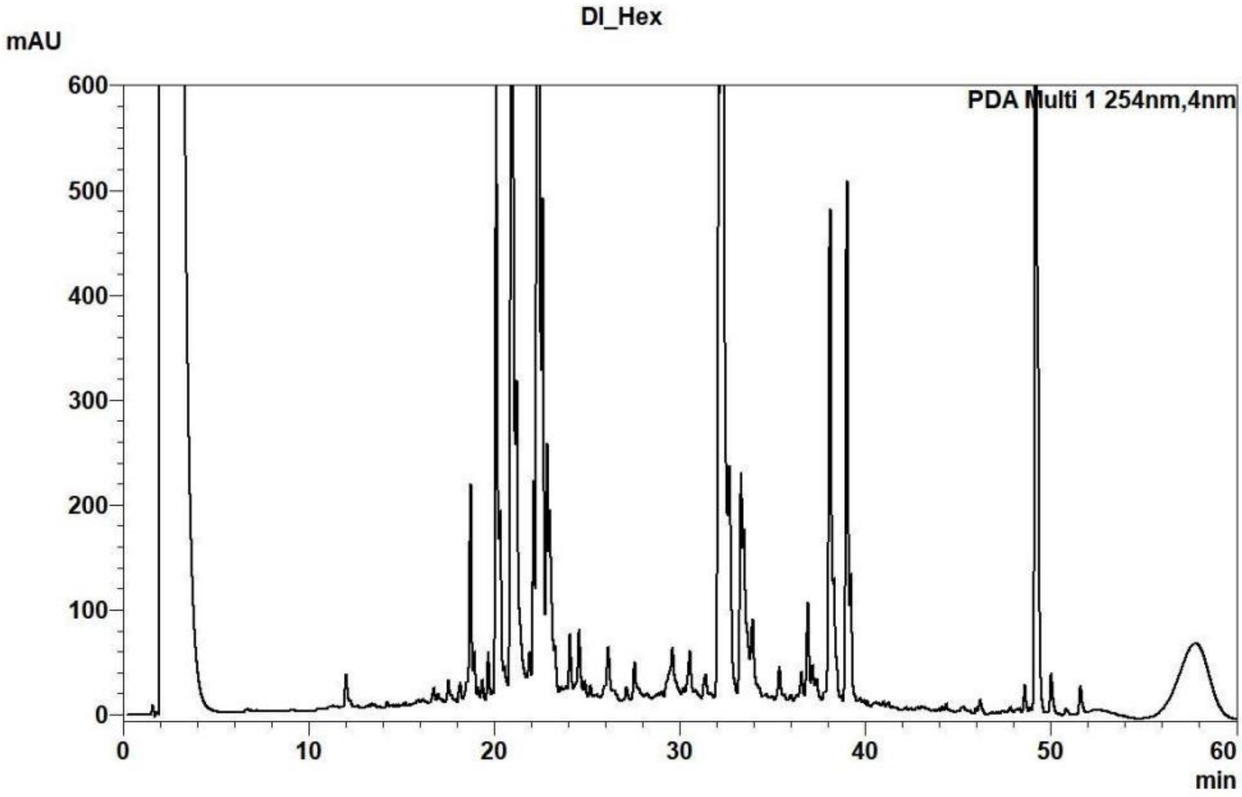
DI_Hex
mAU
PDA Multi 1 254nm,4nm
600
500
400
300
200
100
0
0
10
20
30
40
50
60
min

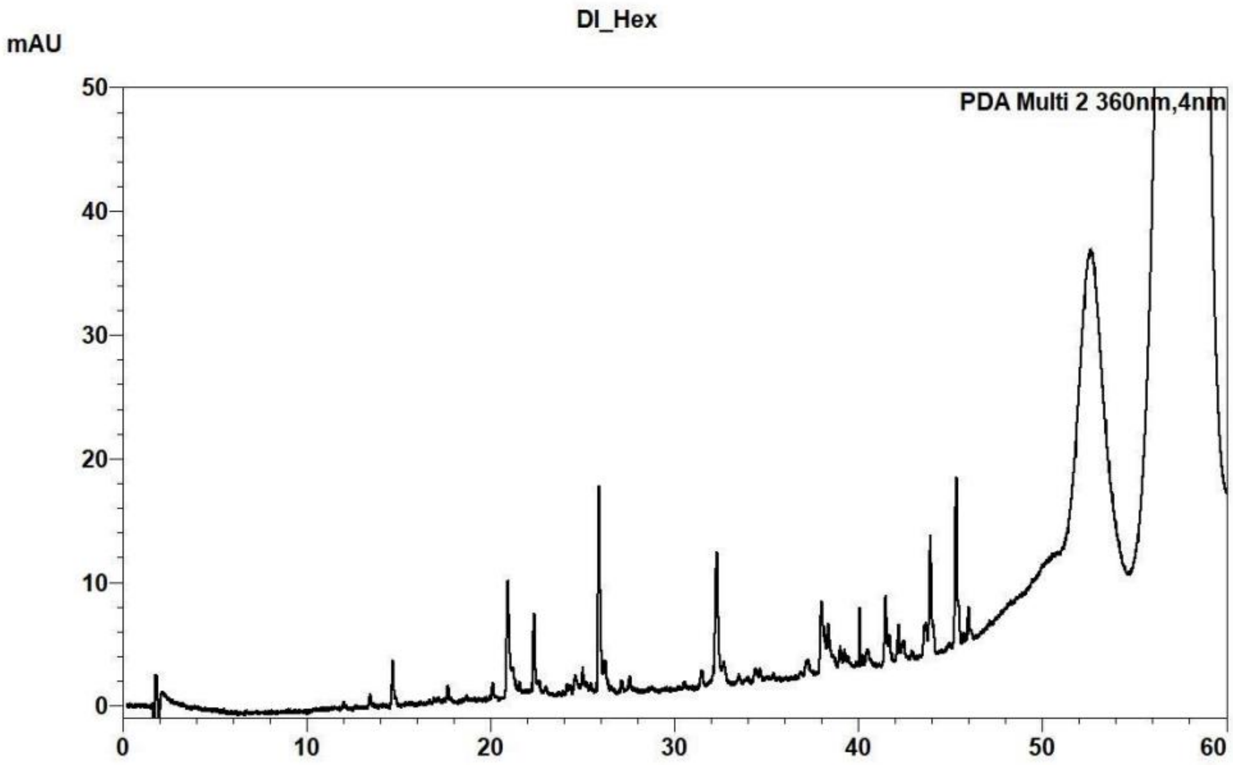
DI_Hex
mAU
PDA Multi 2 360nm,4nm
50
40
30
20
10
0
0
10
20
30
40
50
60

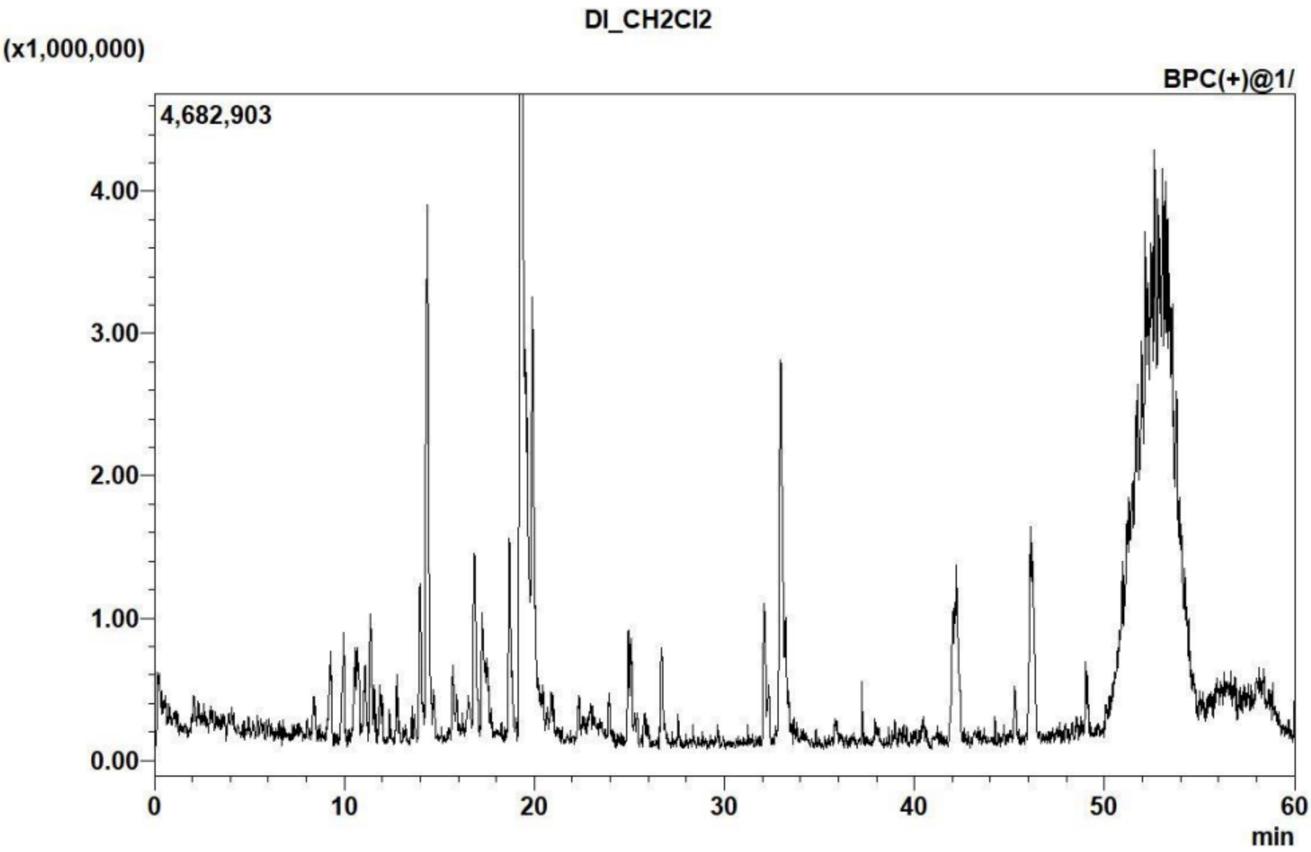
DI_CH2Cl2
(x1,000,000)
BPC(+)@1/
4,682,903
4.00
3.00
2.00
1.00
0.00
0
10
20
30
40
50
60
min

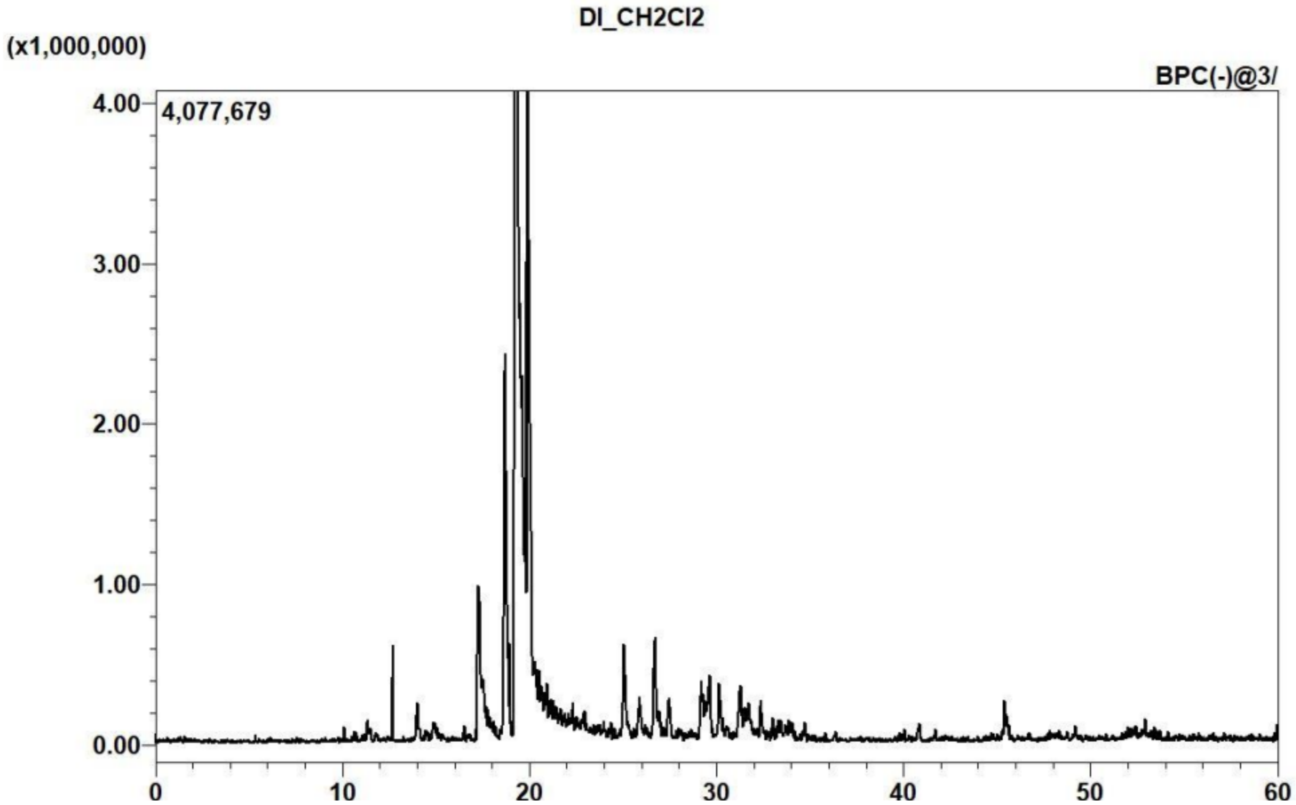
DI_CH2Cl2
(x1,000,000)
BPC(-)@3/
4,077,679
4.00
3.00
2.00
1.00
0.00
0
10
20
30
40
50
60

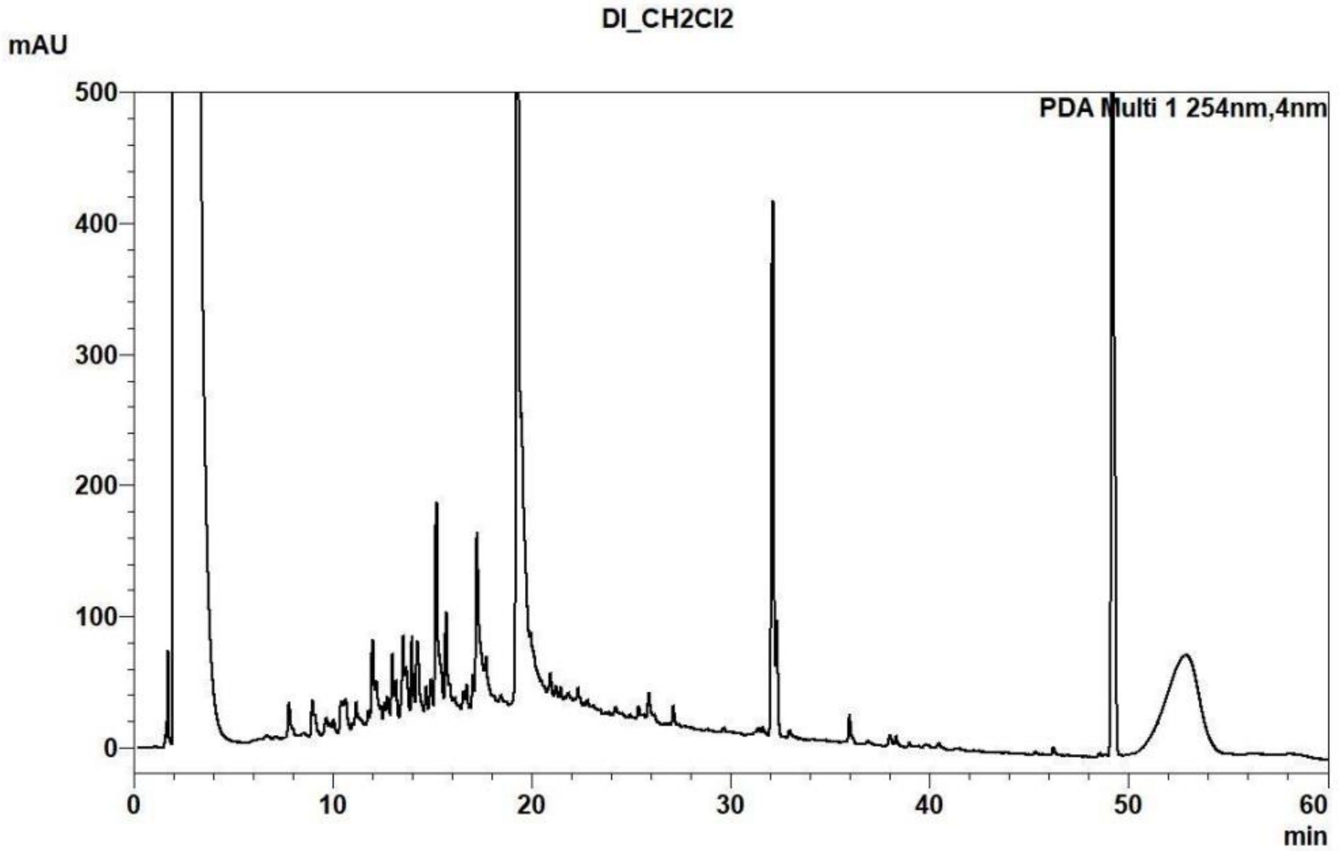

DI_CH2Cl2
mAU
PDA Multi 1 254nm,4nm
500
400
300
200
100
0
0
10
20
30
40
50
60
min

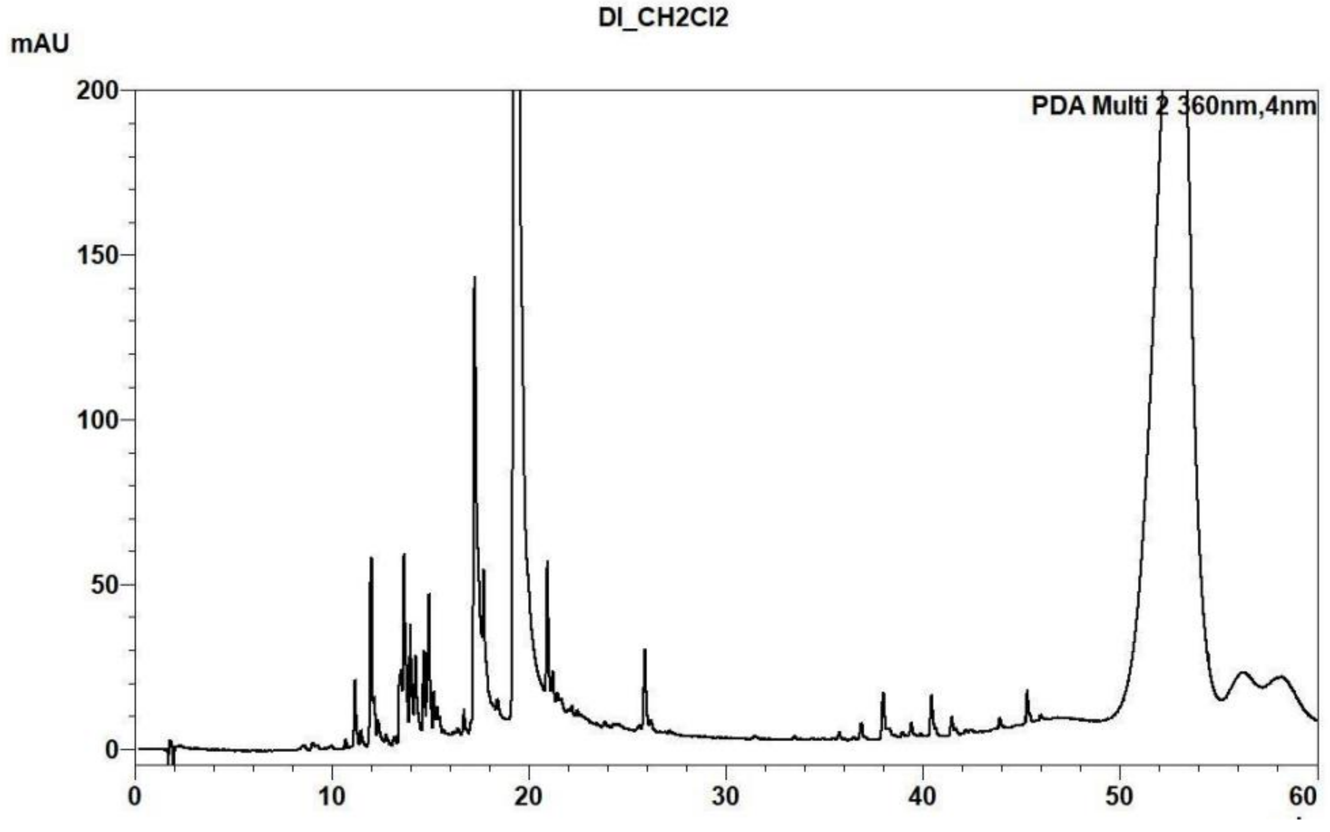

DI_CH2Cl2
mAU
PDA Multi 2 360nm,4nm
200
150
100
50
0
0
10
20
30
40
50
60

Glacium flavum

List of Figures

H_2O phase

Figure S4a. Base Peak Chromatogram (BPC) in the positive ionization mode of water extract of *Glacium flavum*.

Figure S4b. Base Peak Chromatogram (BPC) in the negative ionization mode of water extract of *Glacium flavum*.

Figure S4c. PDA chromatogram of water extracts of *Glacium flavum* at 245 nm.

Figure S4d. PDA chromatogram of water extracts of *Glacium flavum* at 360 nm.

Hexane extract

Figure S5a. Base Peak Chromatogram (BPC) in positive mode of *n*-hexane extract of *Glacium flavum*.

Figure S5b. Base Peak Chromatogram (BPC) in negative mode of *n*-hexane extract of *Glacium fl*avum.

Figure S5c. PDA chromatogram of *n*-hexane extracts of *Glacium flavum* at 245 nm.

Figure S5d. PDA chromatogram of *n*-hexane extracts of *Glacium flavum* at 360 nm.

CH2Cl2 extract

Figure S6a. Base Peak Chromatogram (BPC) in positive mode of dichloromethane extract of *Glacium* flavum.

Figure S6b. Base Peak Chromatogram (BPC) in negative mode of dichloromethane extract of *Glacium flavum*.

Figure S6c. PDA chromatogram of dichloromethane extracts of *Glacium flavum* at 245 nm.

Figure S6d. PDA chromatogram of dichloromethane of *Glacium flavum* at 360 nm.

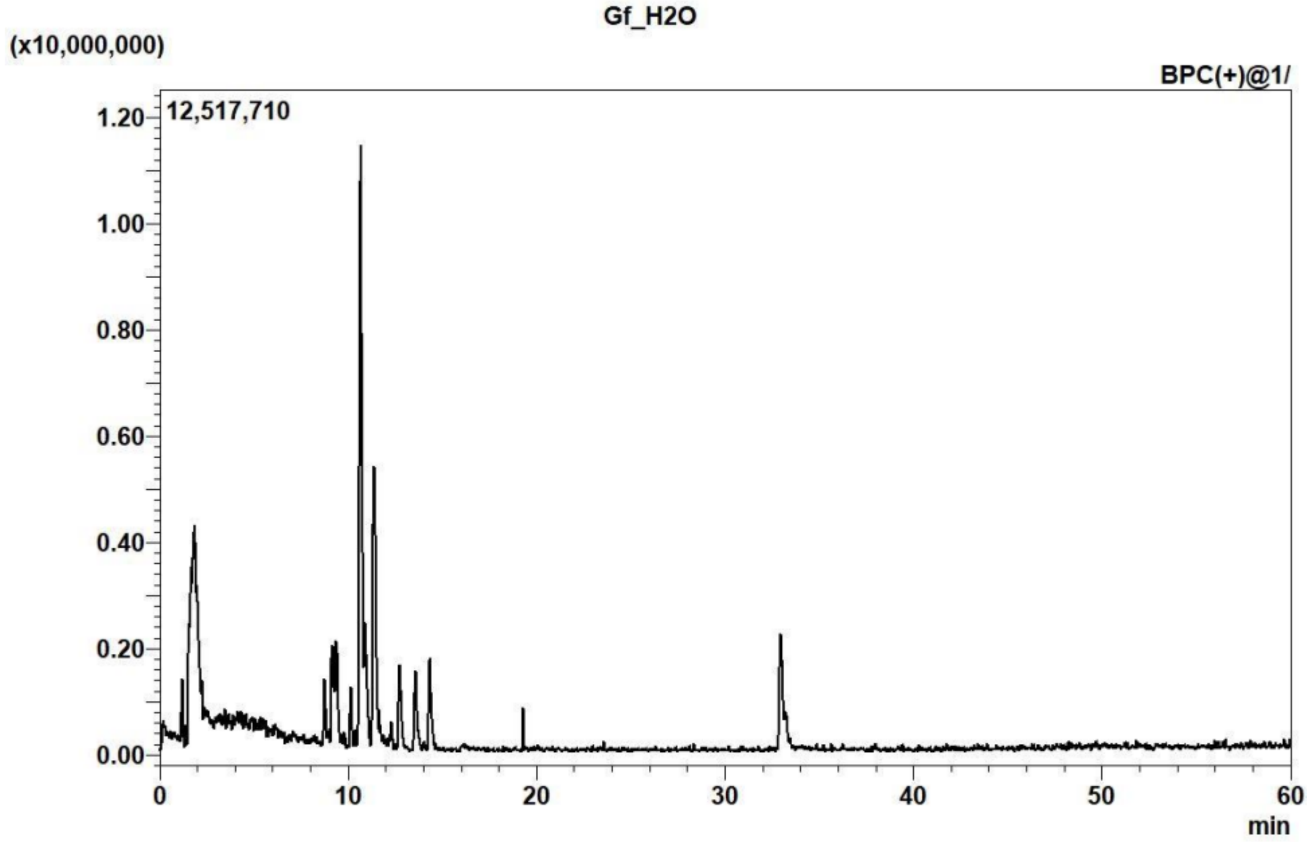
Gf_H2O
(x10,000,000)
BPC(+)@1/
12,517,710
1.20
1.00
0.80
0.60
0.40
0.20
0.00
0
10
20
30
40
50
60
min

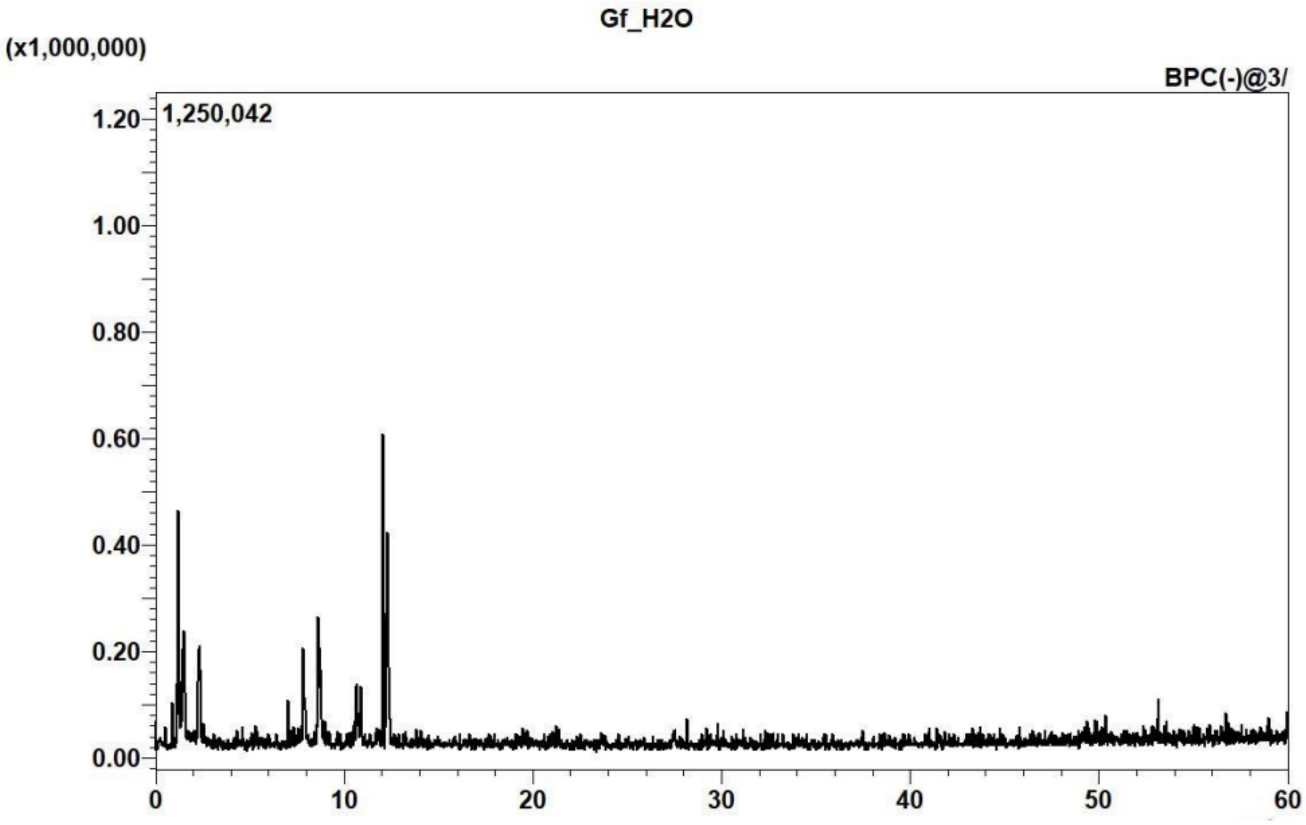
Gf_H2O
(x1,000,000)
BPC(-)@3/
1,250,042
1.20
1.00
0.80
0.60
0.40
0.20
0.00
0
10
20
30
40
50
60

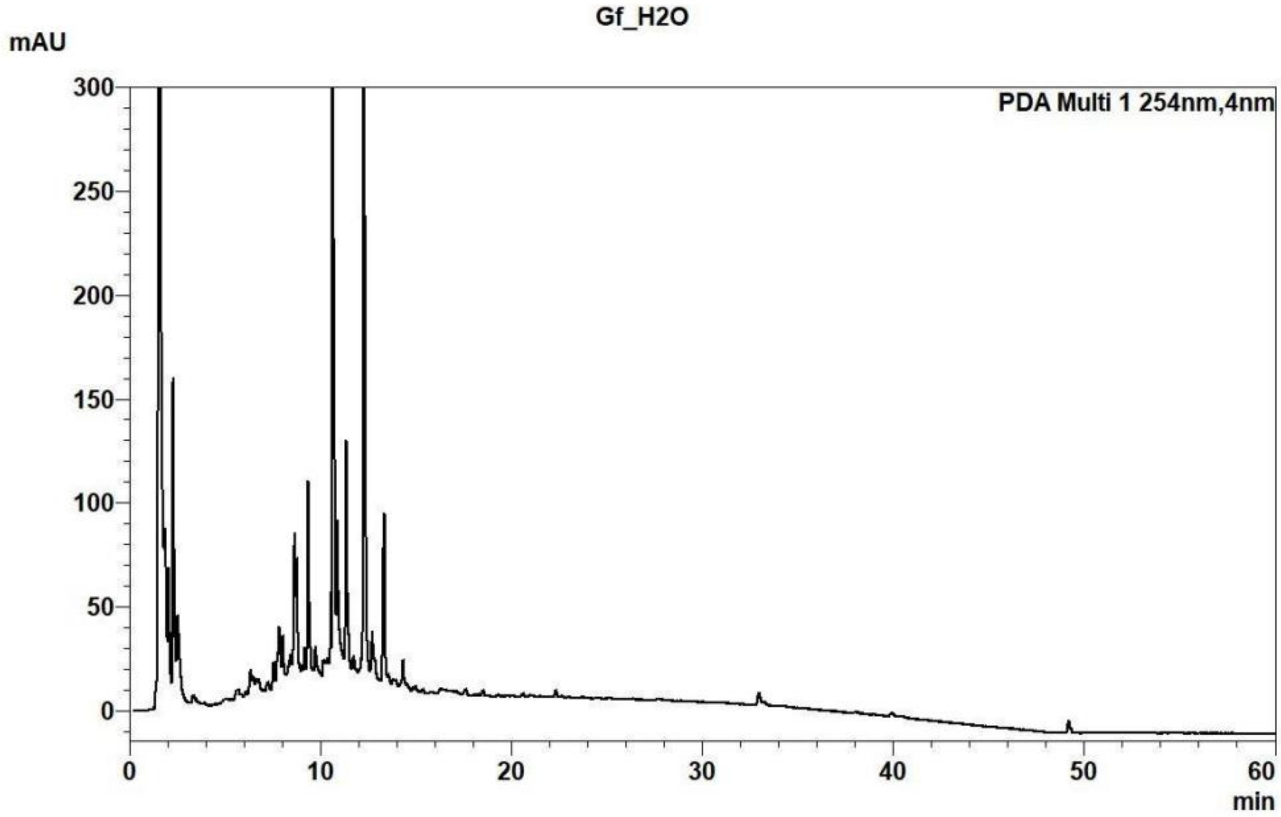
Gf_H2O
mAU
PDA Multi 1 254nm,4nm
300
250
200
150
100
50
0
0
10
20
30
40
50
60
min

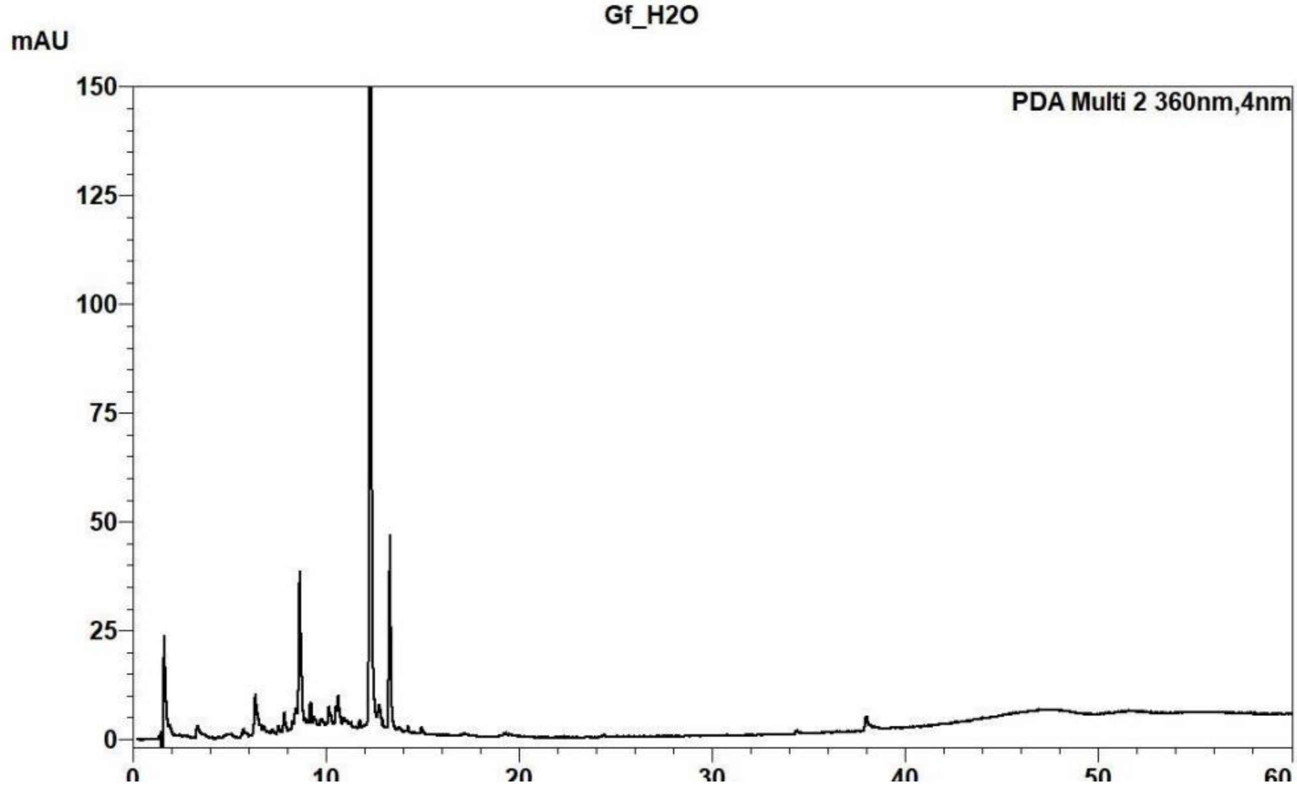
Gf_H2O
mAU
PDA Multi 2 360nm,4nm
150
125
100
75
50
25
0
0
10
20
30
40
50
60

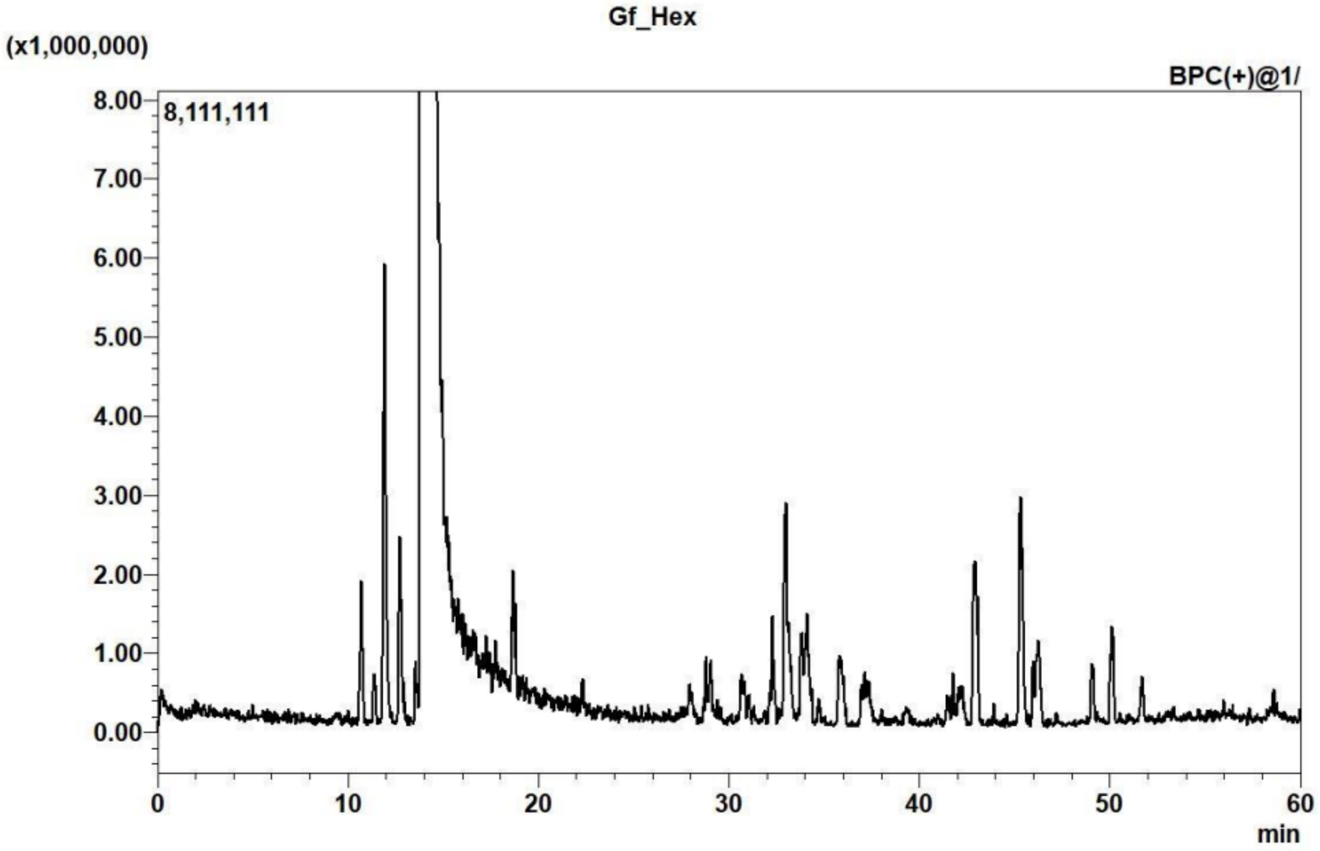
Gf_Hex
(x1,000,000)
BPC(+)@1/
8,111,111
8.00
7.00
6.00
5.00
4.00
3.00
2.00
1.00
0.00
0
10
20
30
40
50
60
min

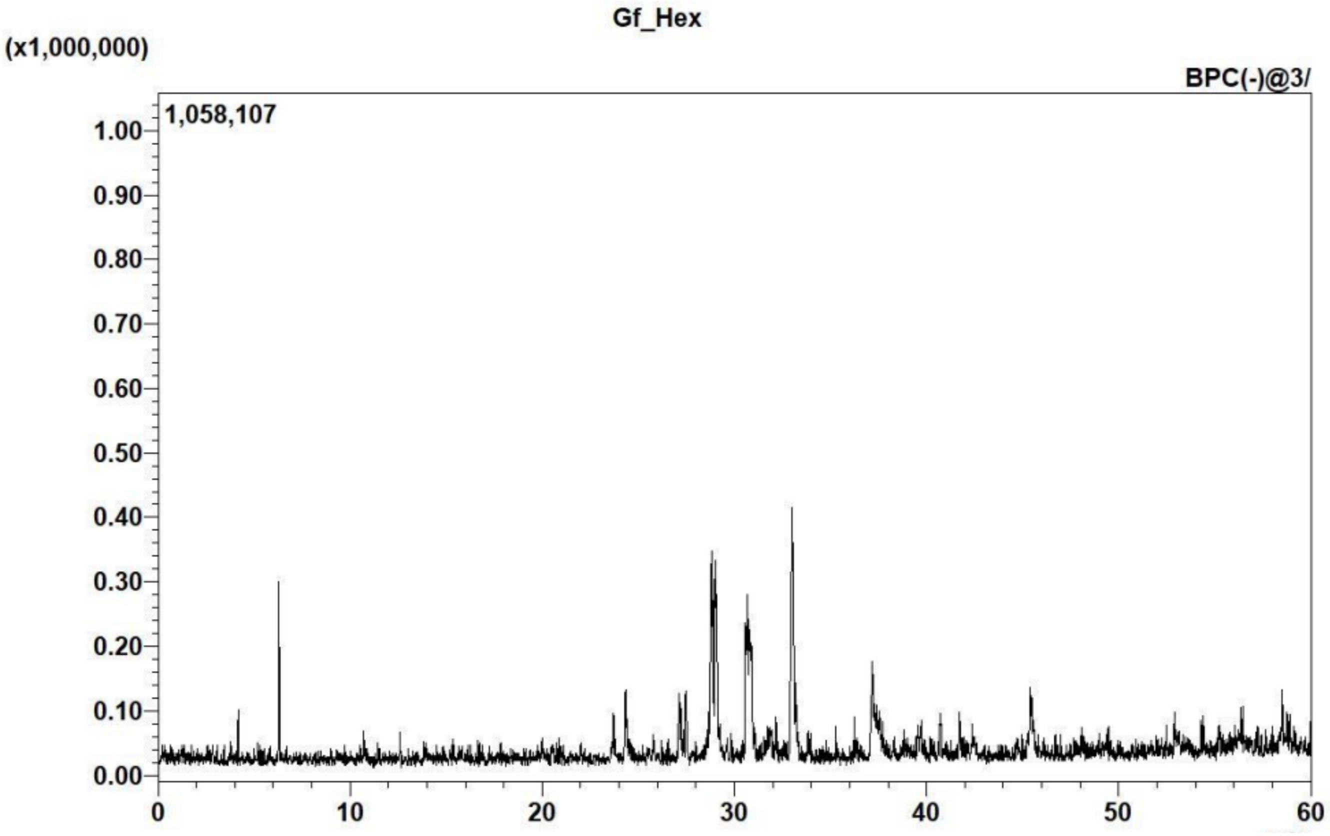
Gf_Hex
(x1,000,000)
BPC(-)@3/
1,058,107
1.00
0.90
0.80
0.70
0.60
0.50
0.40
0.30
0.20
0.10
0.00
0
10
20
30
40
50
60

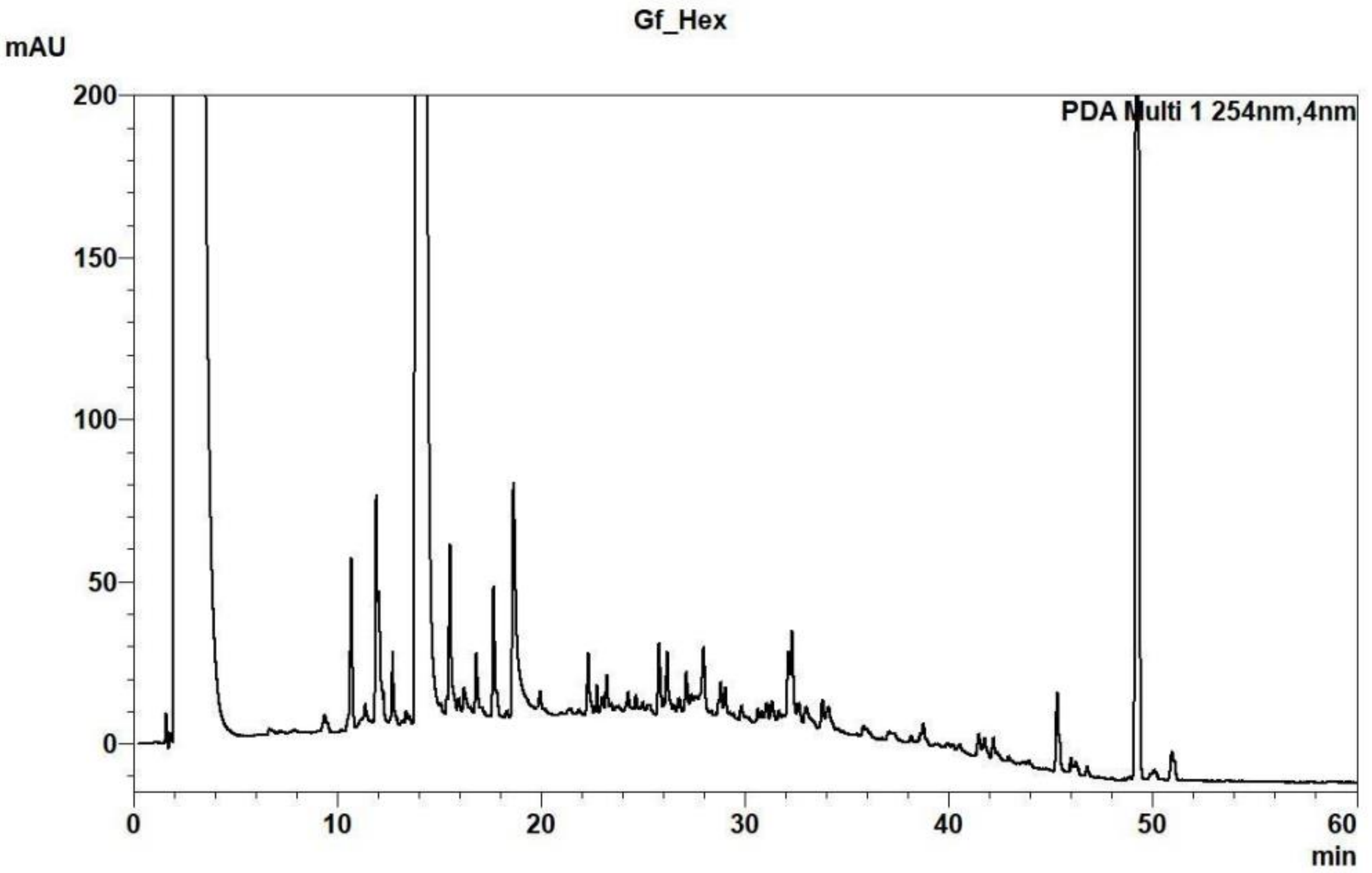
Gf_Hex
mAU
PDA Multi 1 254nm,4nm
200
150
100
50
0
0
10
20
30
40
50
60
min

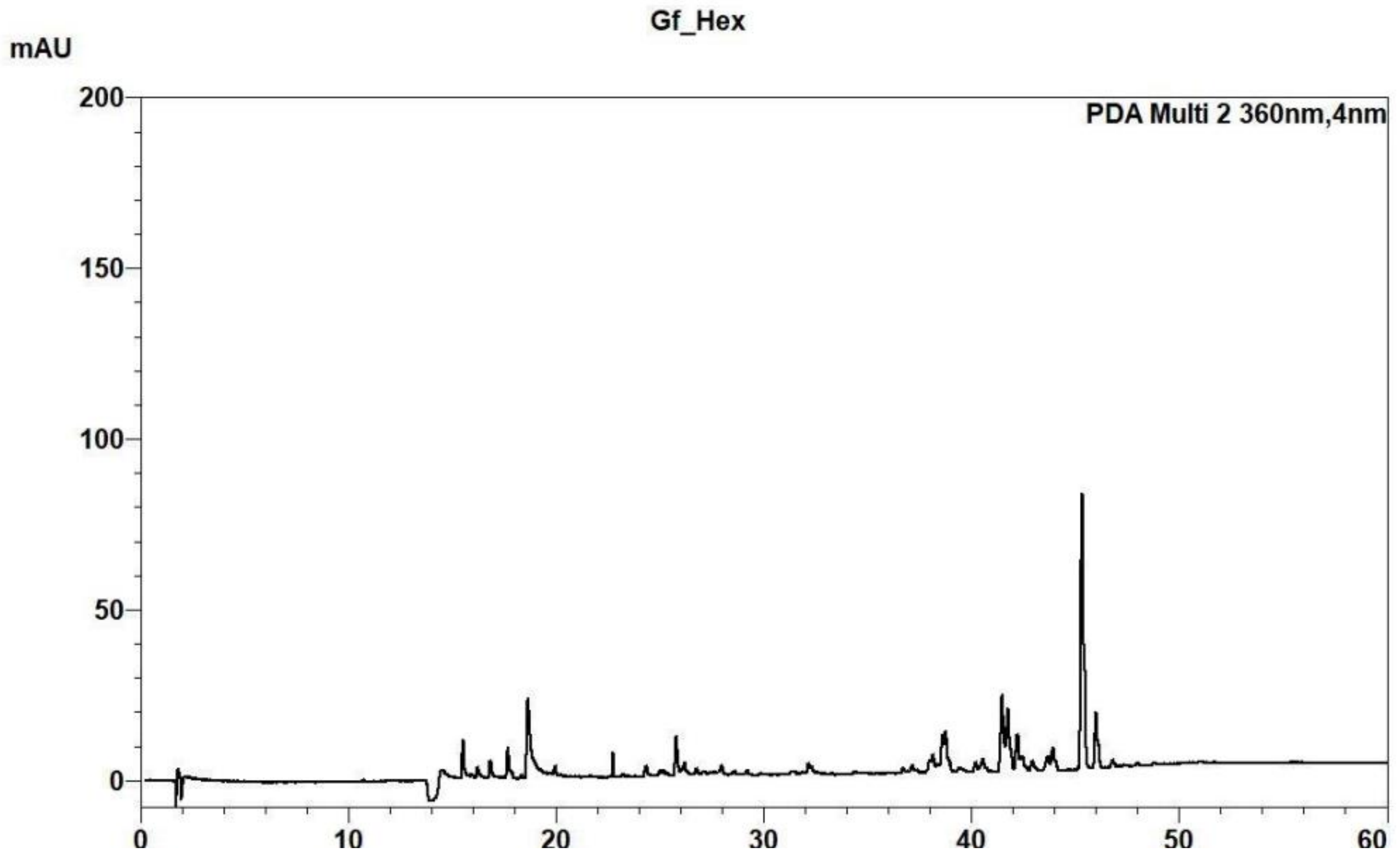
Gf_Hex
mAU
PDA Multi 2 360nm,4nm
200
150
100
50
0
0
10
20
30
40
50
60

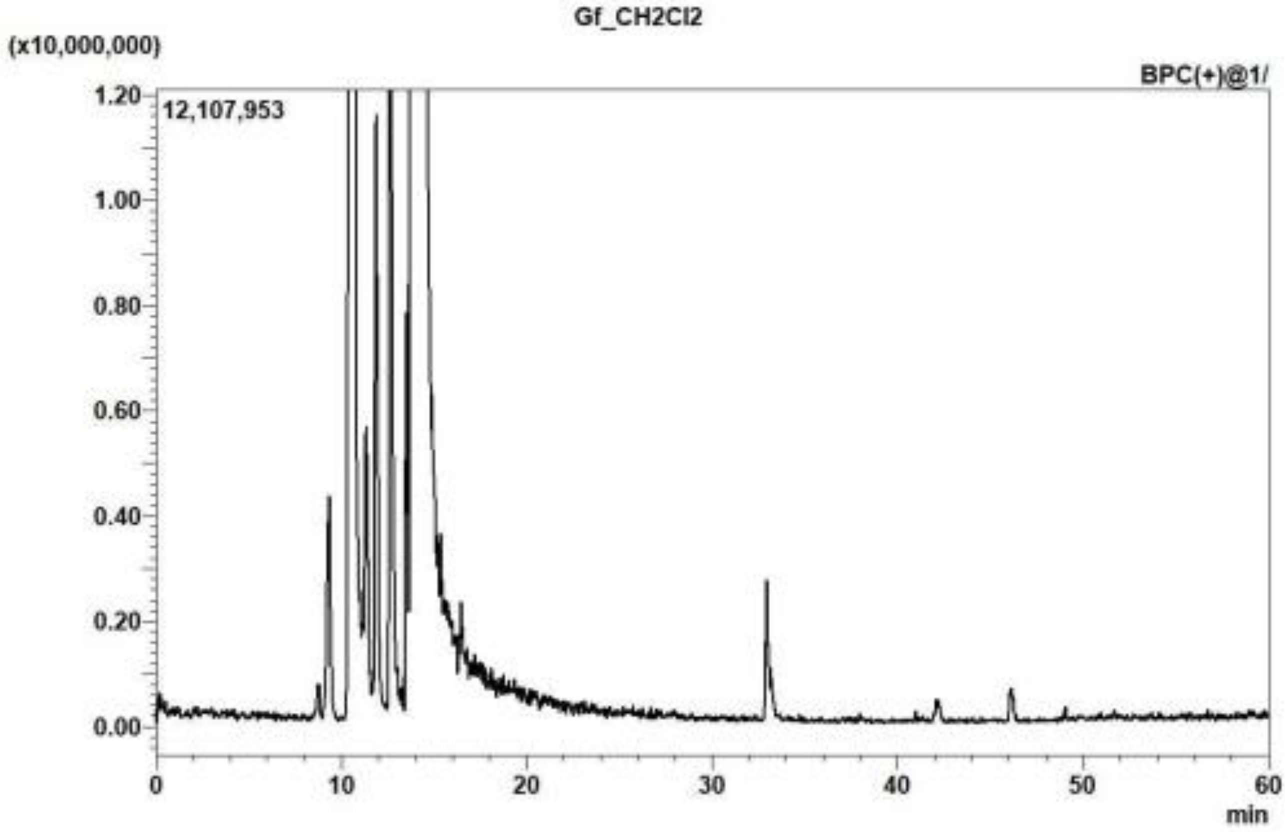
Gf_CH2Cl2
(x10,000,000)
BPC(+)@1/
12,107,953
1.20
1.00
0.80
0.60
0.40
0.20
0.00
0
10
20
30
40
50
60
min

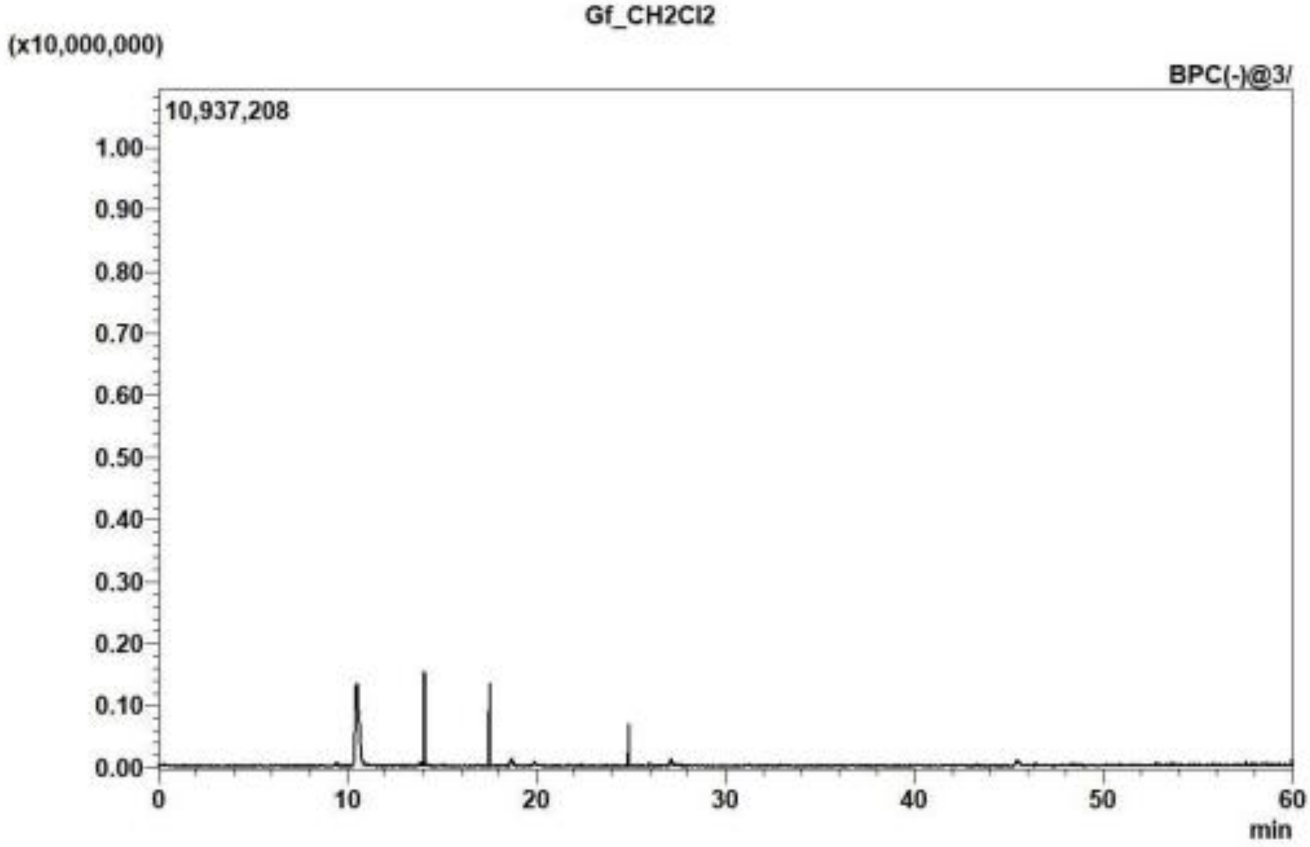
Gf_CH2Cl2
(x10,000,000)
BPC(-)@3/
10,937,208
1.00
0.90
0.80
0.70
0.60
0.50
0.40
0.30
0.20
0.10
0.00
0
10
20
30
40
50
60
min

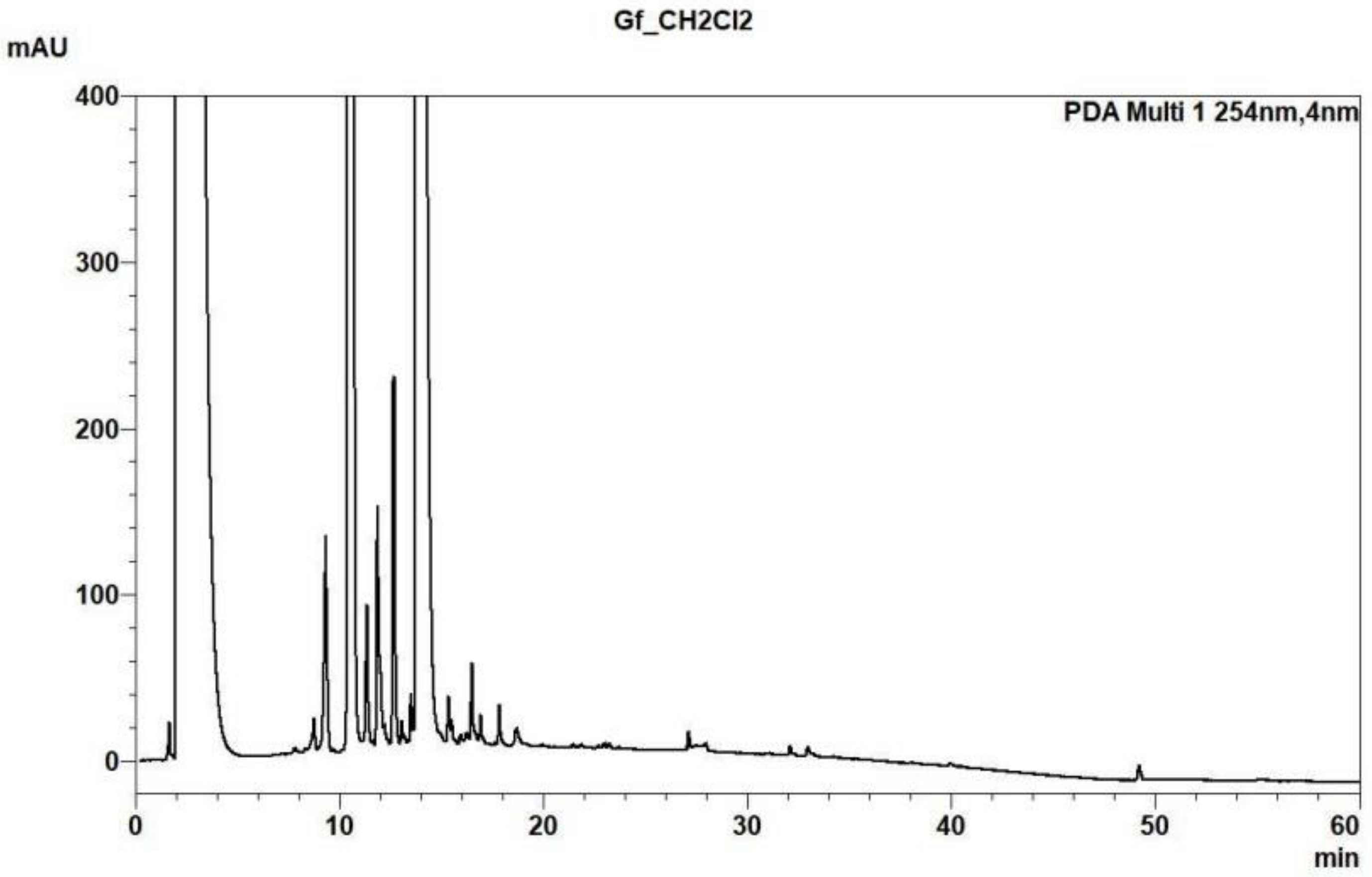
Gf_CH2Cl2
mAU
PDA Multi 1 254nm,4nm
400
300
200
100
0
0
10
20
30
40
50
60
min

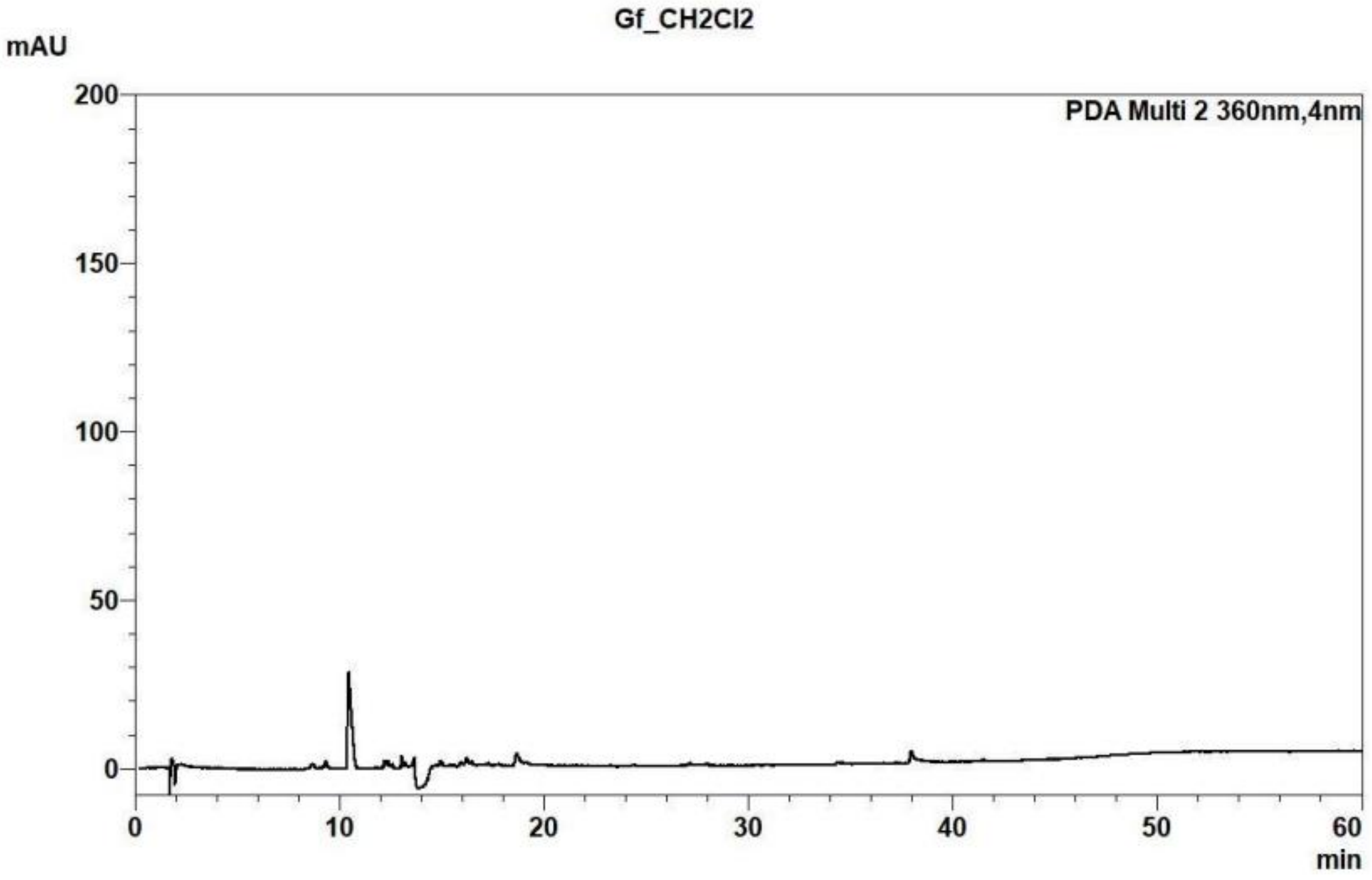
Gf_CH2Cl2
mAU
PDA Multi 2 360nm,4nm
200
150
100
50
0
0
10
20
30
40
50
60
min

Hypericum egyptum

List of Figures

H_2O phase

Figure S7a. Base Peak Chromatogram (BPC) in the positive ionization mode of water extract of *Hypericum egyptum*.

Figure S7b. Base Peak Chromatogram (BPC) in the negative ionization mode of water extract of *Hypericum egyptum*.

Figure S7c. PDA chromatogram of water extracts of *Hypericum egyptum* at 245 nm.

Figure S7d. PDA chromatogram of water extracts of *Hypericum egyptum* at 360 nm.

Hexane extract

Figure S8a. Base Peak Chromatogram (BPC) in positive mode of *n*-hexane extract of *Hypericum egyptum*.

Figure S8b. Base Peak Chromatogram (BPC) in negative mode of *n*-hexane extract of *Hypericum egyptum*.

Figure S8c. PDA chromatogram of *n*-hexane extracts of *Hypericum egyptum* at 245 nm.

Figure S8d. PDA chromatogram of *n*-hexane extracts of *Hypericum egyptum at* 360 nm.

CH2Cl2 extract

Figure S9a. Base Peak Chromatogram (BPC) in positive mode of dichloromethane extract of *Hypericum egyptum*.

Figure S9b. Base Peak Chromatogram (BPC) in negative mode of dichloromethane extract of *Hypericum egyptum*.

Figure S9c. PDA chromatogram of dichloromethane extracts of *Hypericum egyptum* at 245 nm.

Figure S9d. PDA chromatogram of dichloromethane of *Hypericum egyptum* at 360 nm.

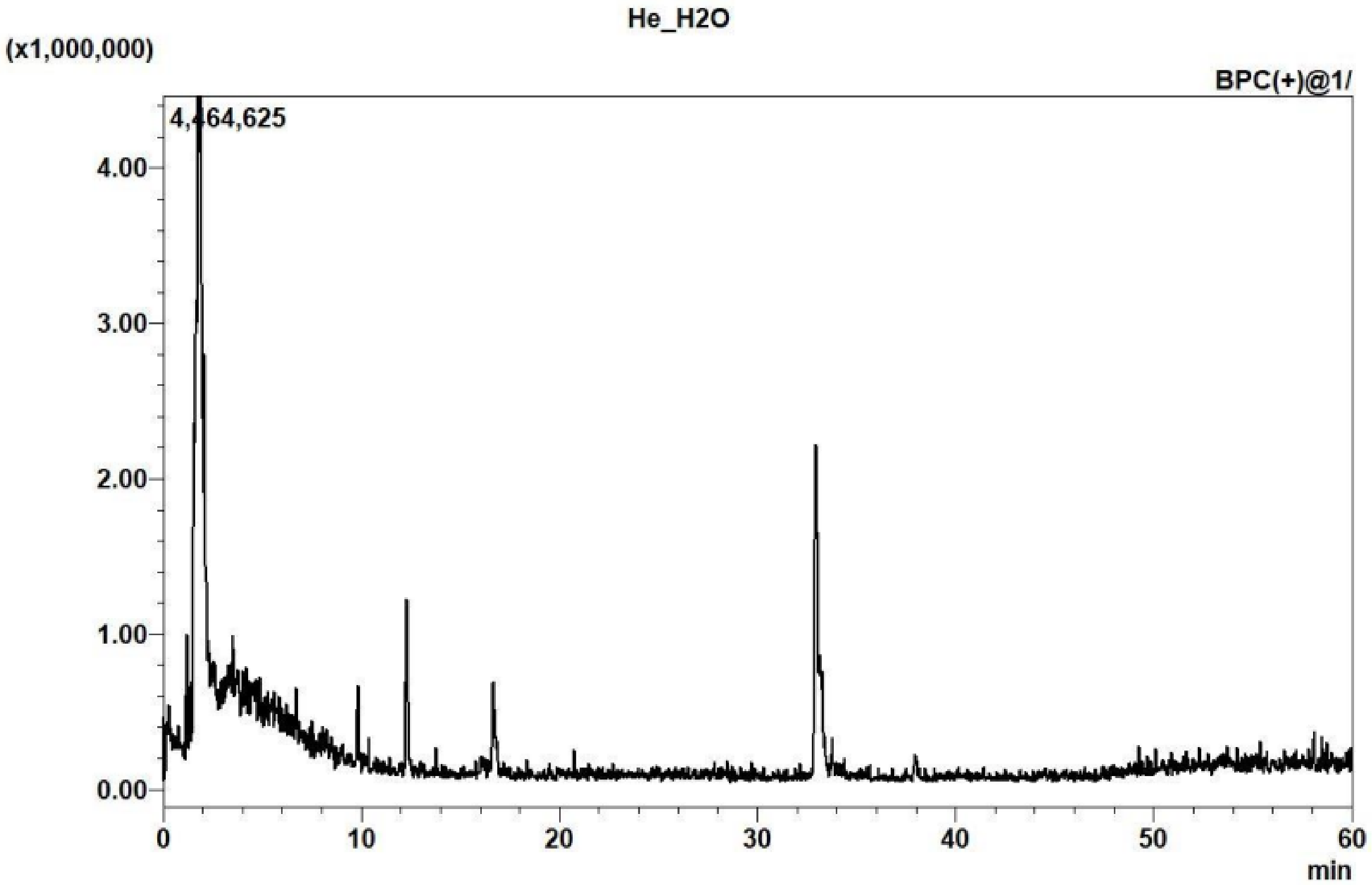
He_H2O
(x1,000,000)
BPC(+)@1/
4,464,625
4.00
3.00
2.00
1.00
0.00
0
10
20
30
40
50
60
min

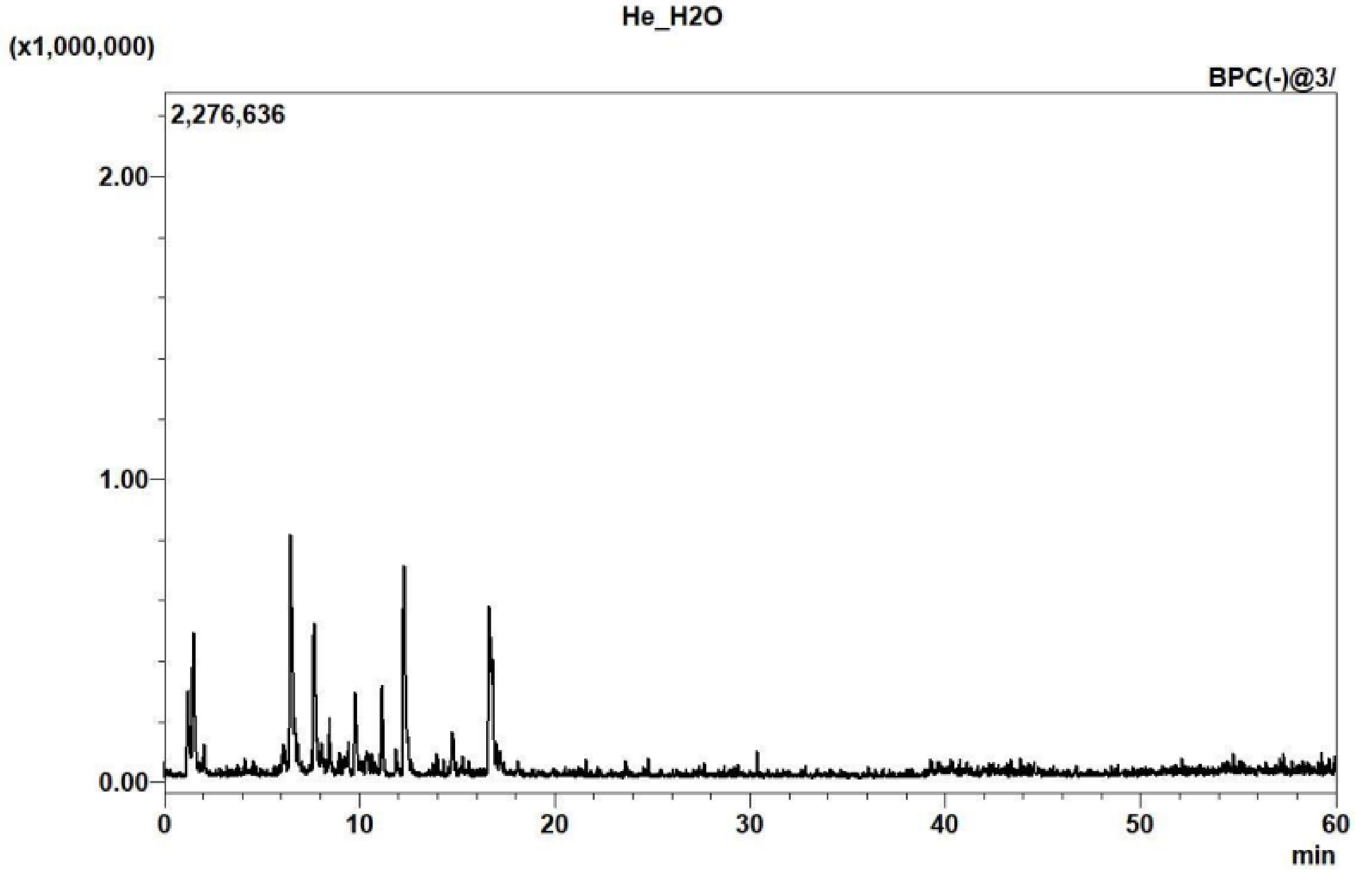
He_H2O
(x1,000,000)
BPC(-)@3/
2,276,636
2.00
1.00
0.00
0
10
20
30
40
50
60
min

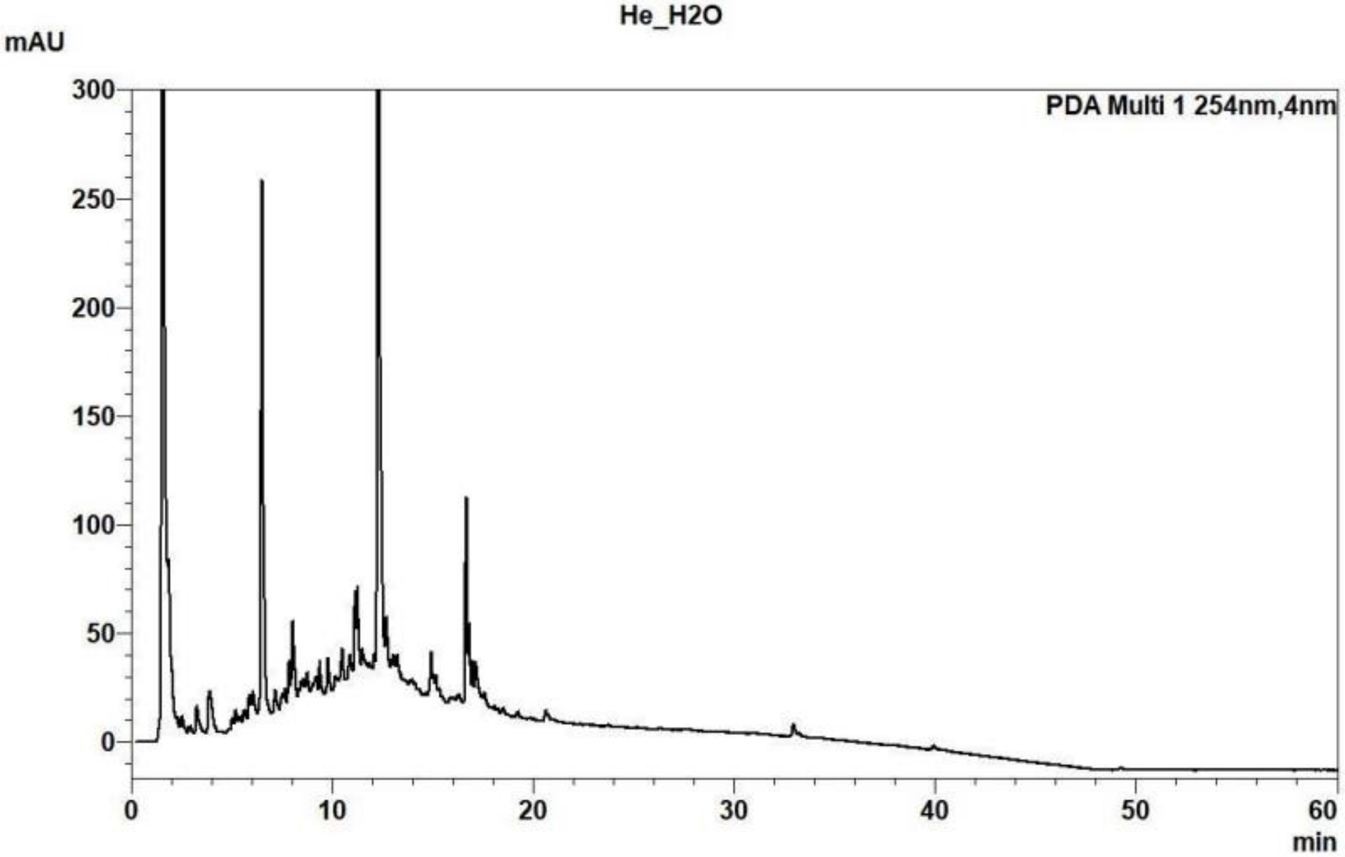
He_H2O
mAU
PDA Multi 1 254nm,4nm
300
250
200
150
100
50
0
0
10
20
30
40
50
60
min

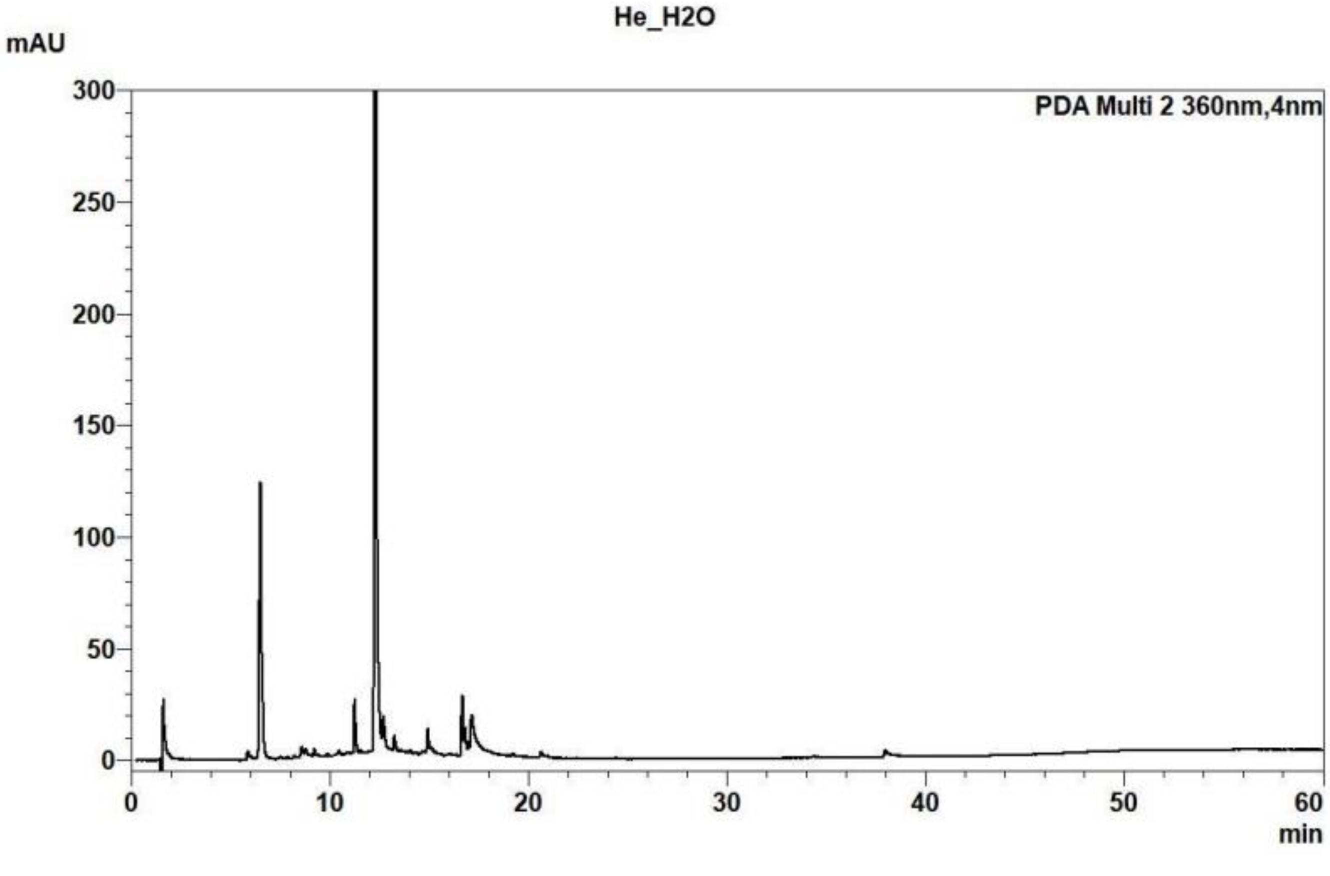
He_H2O
mAU
PDA Multi 2 360nm,4nm
300
250
200
150
100
50
0
0
10
20
30
40
50
60
min

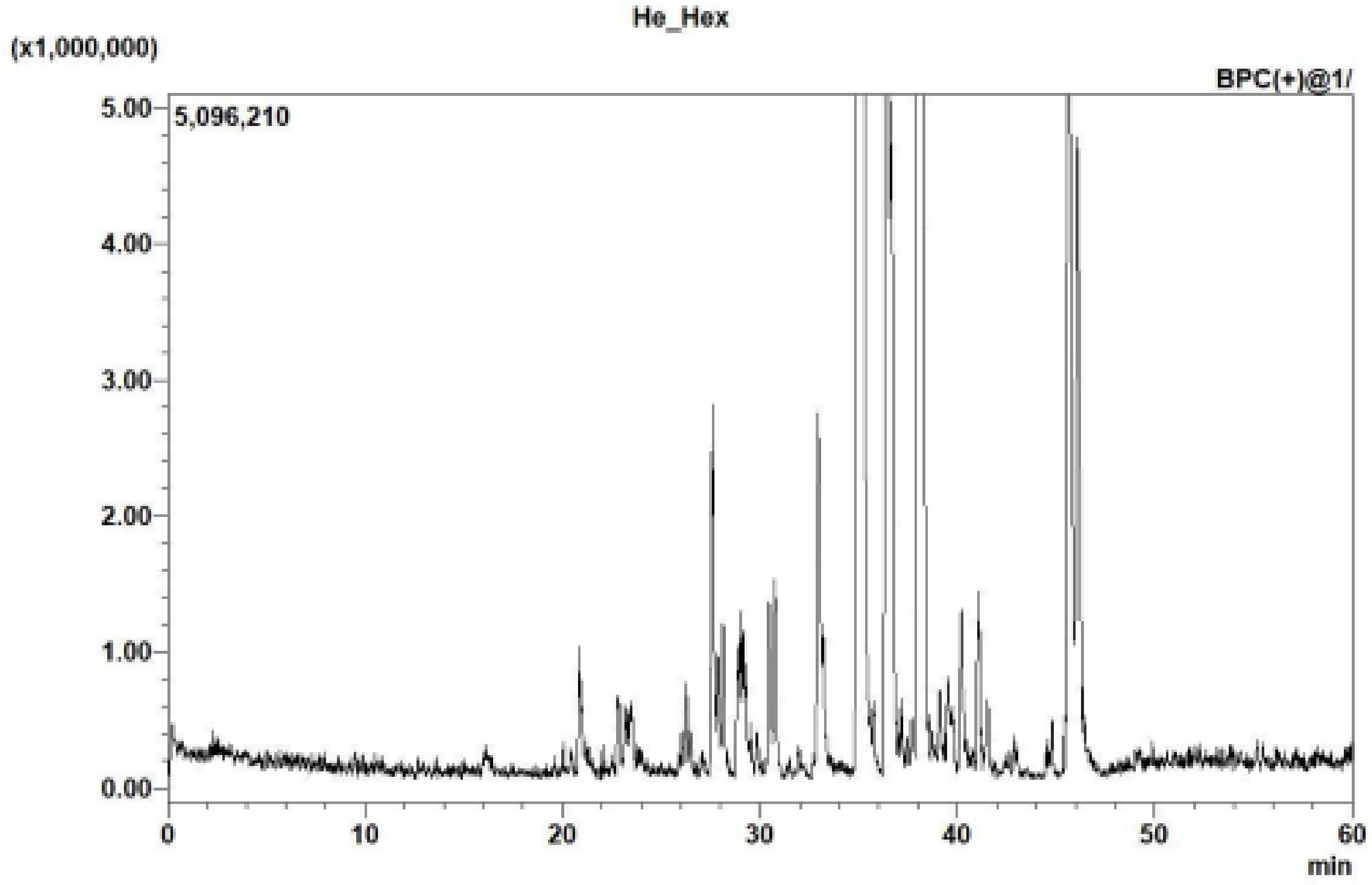
He_Hex
(x1,000,000)
BPC(+)@1/
5,096,210
5.00
4.00
3.00
2.00
1.00
0.00
0
10
20
30
40
50
60
min

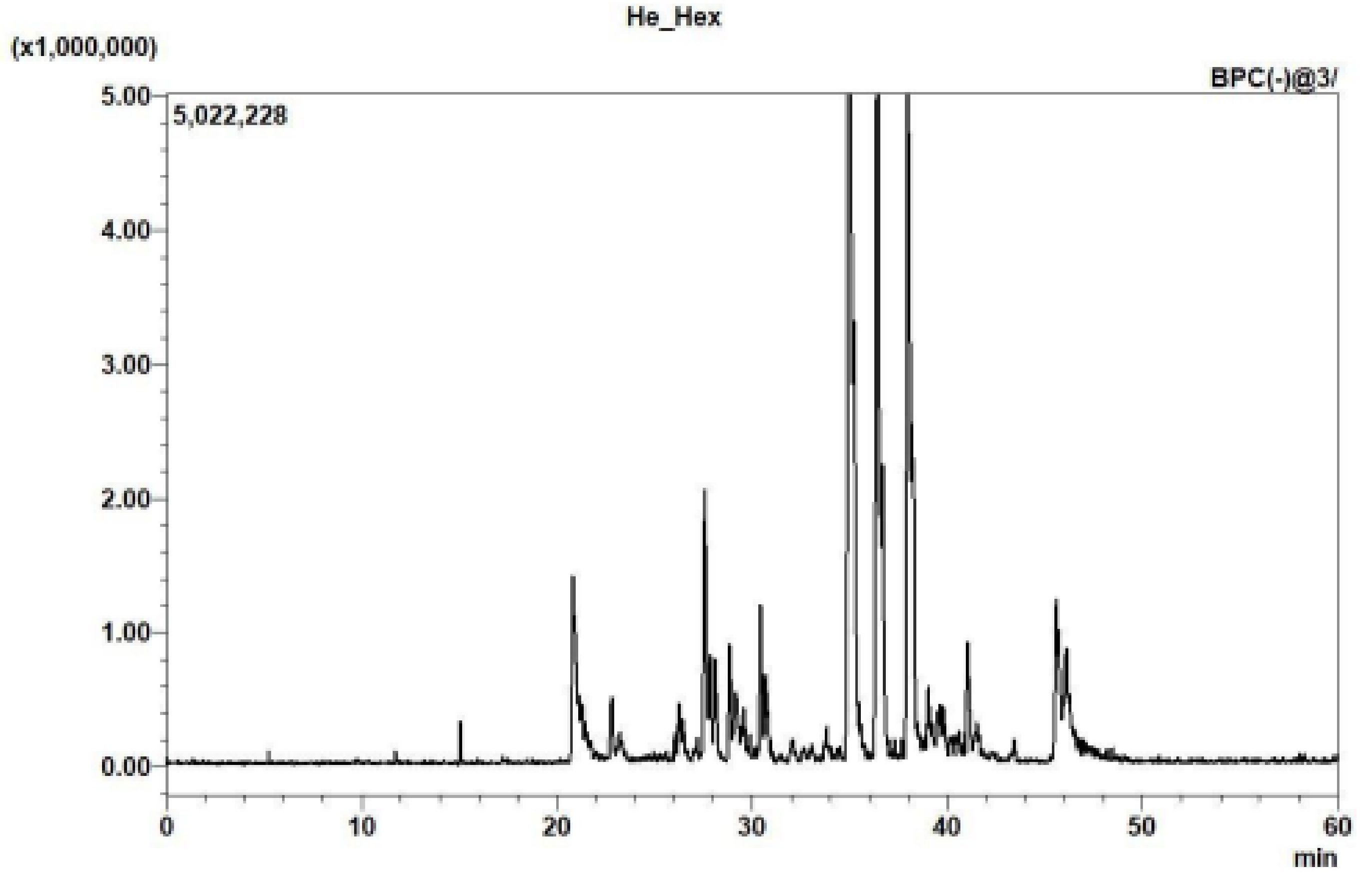
He_Hex
(x1,000,000)
BPC(-)@3/
5,022,228
5.00
4.00
3.00
2.00
1.00
0.00
0
10
20
30
40
50
60
min

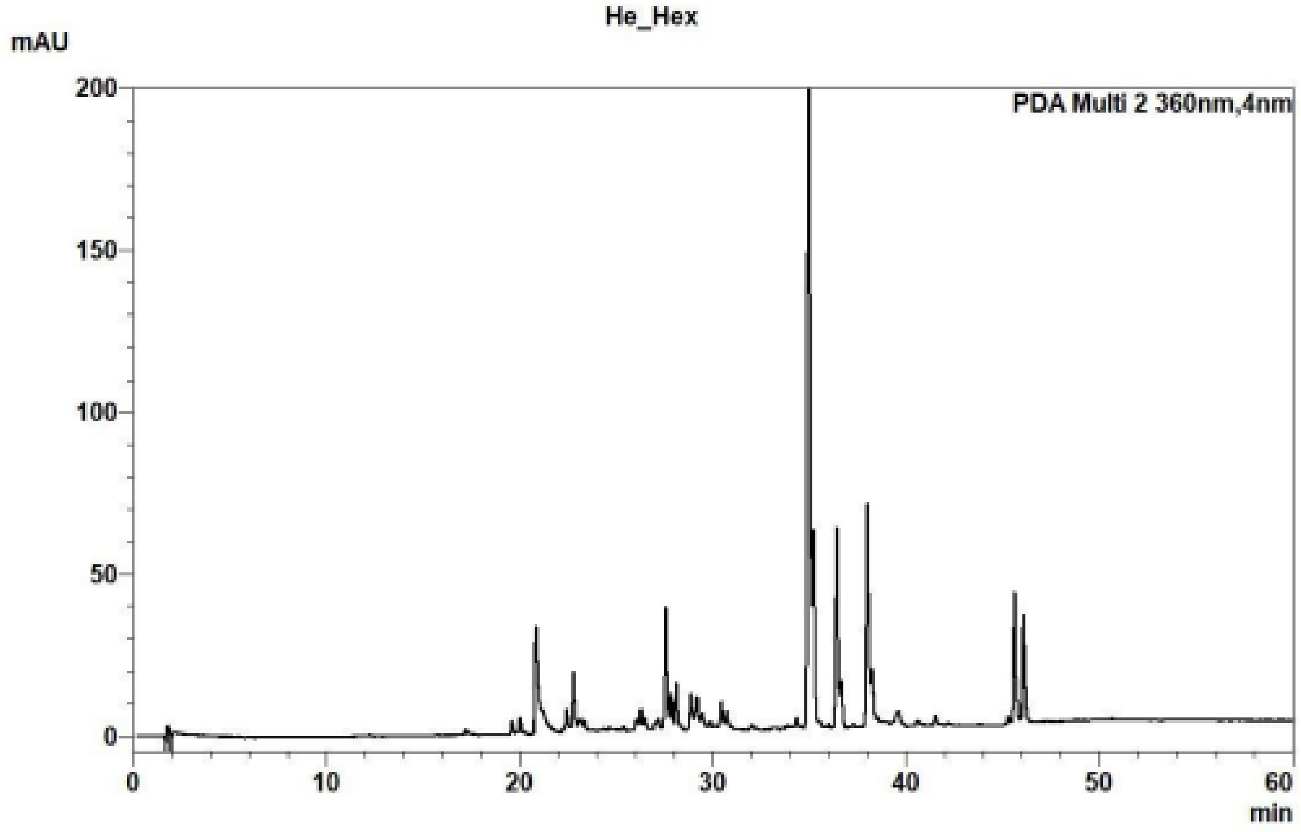
He_Hex
mAU
PDA Multi 2 360nm,4nm
200
150
100
50
0
0
10
20
30
40
50
60
min

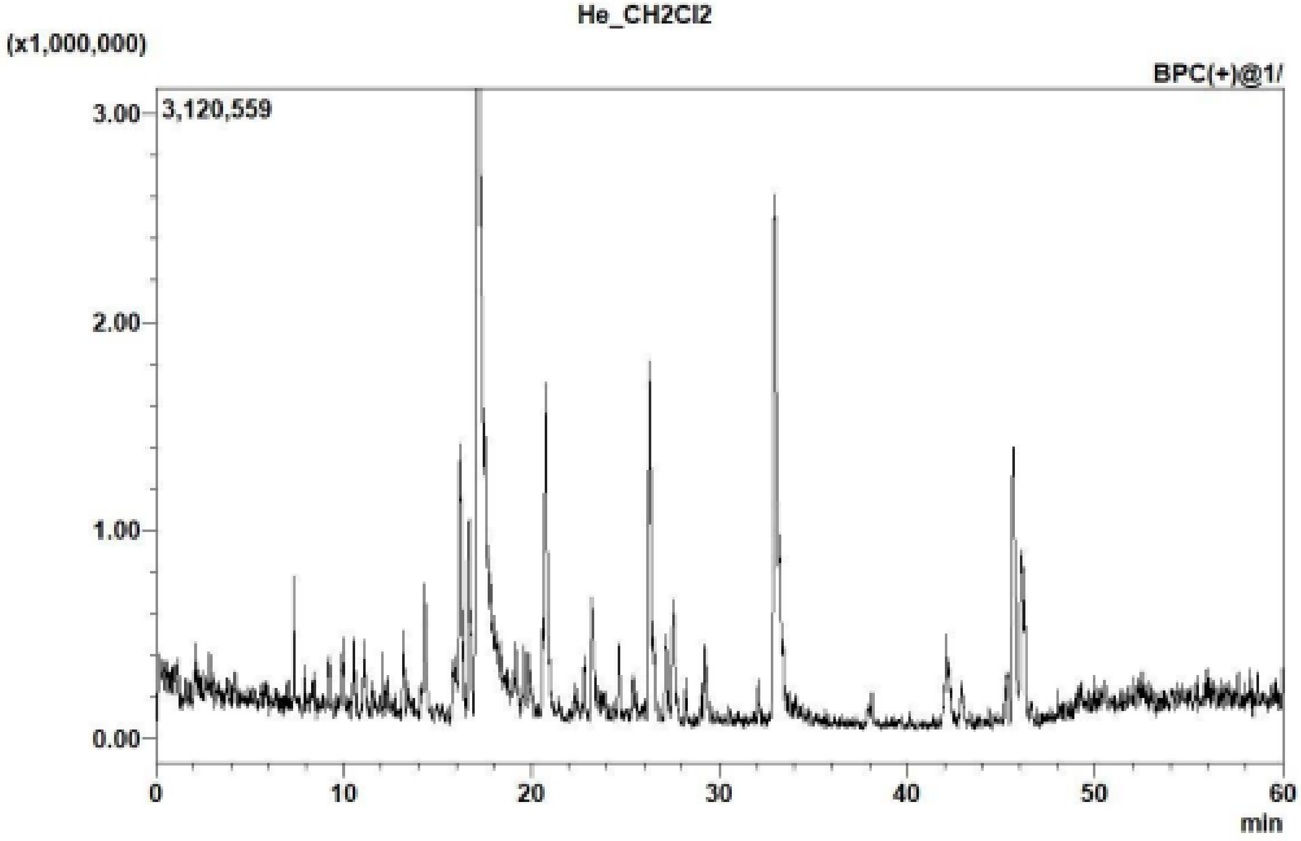
He_CH2Cl2
(x1,000,000)
BPC(+)@1/
3,120,559
3.00
2.00
1.00
0.00
0
10
20
30
40
50
60
min

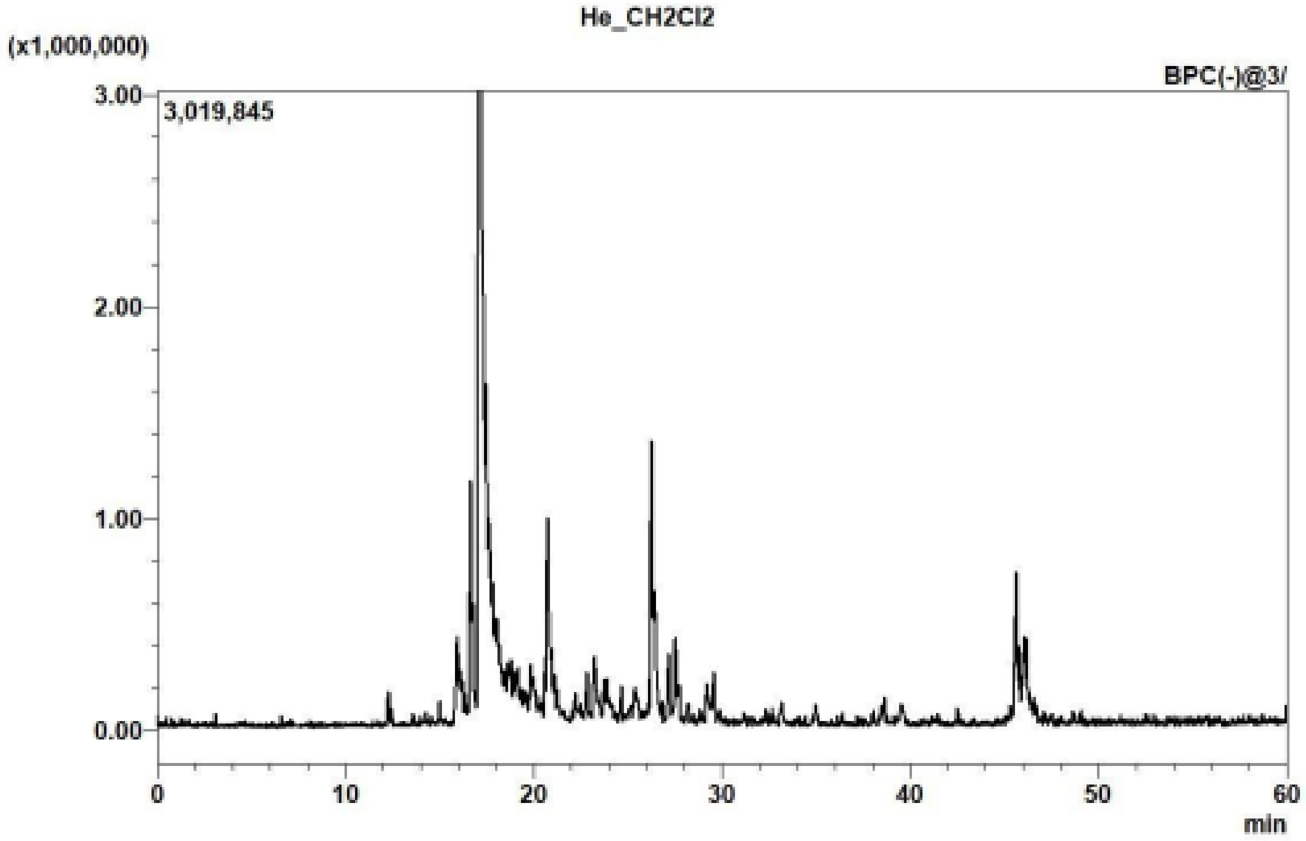
He_CH2Cl2
(x1,000,000)
BPC(-)@3/
3,019,845
3.00
2.00
1.00
0.00
0
10
20
30
40
50
60
min

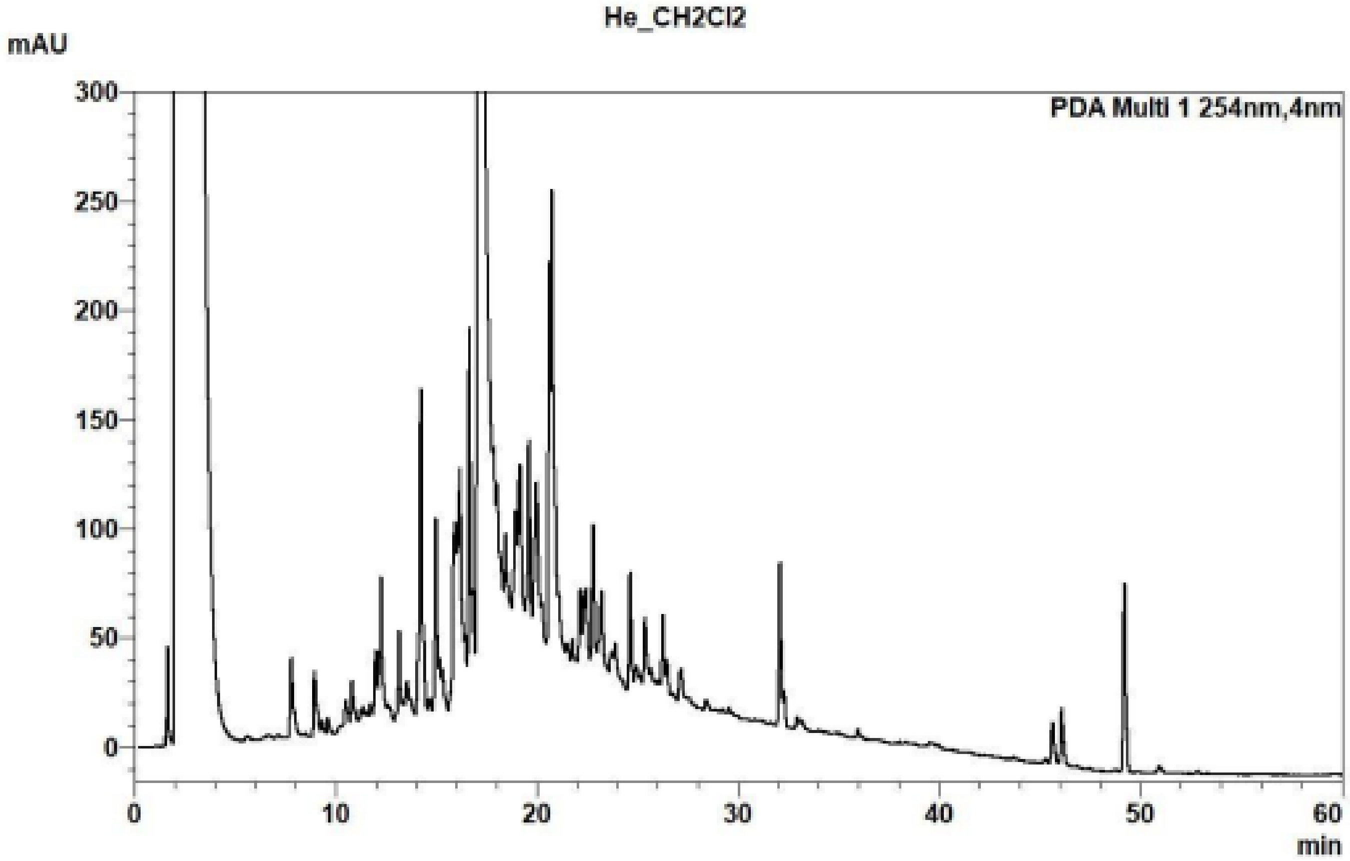
He_CH2Cl2
mAU
PDA Multi 1 254nm,4nm
300
250
200
150
100
50
0
0
10
20
30
40
50
60
min

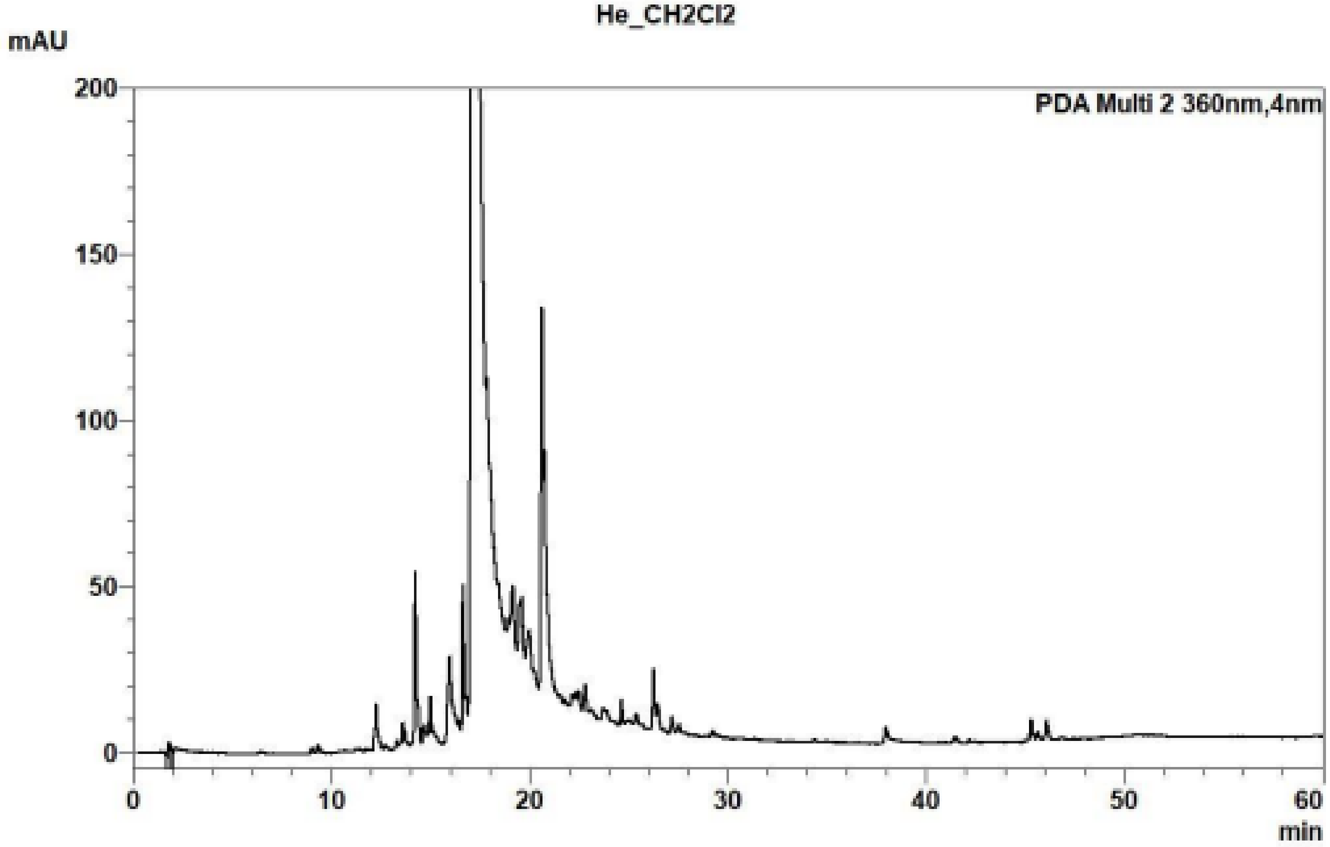
He_CH2Cl2
mAU
PDA Multi 2 360nm,4nm
200
150
100
50
0
0
10
20
30
40
50
60
min

Periploca angustifolia

List of Figures

H_2O phase

Figure S10a. Base Peak Chromatogram (BPC) in the positive ionization mode of water extract of *Periploca angustifolia*.

Figure S10b. Base Peak Chromatogram (BPC) in the negative ionization mode of water extract of Periploca angustifolia.

Figure S10c. PDA chromatogram of water extracts of *Periploca angustifolia* at 245 nm.

Figure S10d. PDA chromatogram of water extracts of *Periploca angustifolia* at 360 nm. Hexane extract

Figure S11a. Base Peak Chromatogram (BPC) in positive mode of *n*-hexane extract of *Periploca angustifolia*.

Figure S11b. Base Peak Chromatogram (BPC) in negative mode of *n*-hexane extract of *Periploca angustifolia.*

Figure S11c. PDA chromatogram of *n*-hexane extracts of *Periploca angus*tifolia at 245 nm.

Figure S11d. PDA chromatogram of *n*-hexane extracts of *Periploca angustifolia* at 360 nm.

CH2Cl2 extract

Figure S12a. Base Peak Chromatogram (BPC) in positive mode of dichloromethane extract of *Periploca angustifolia.*

Figure S12b. Base Peak Chromatogram (BPC) in negative mode of dichloromethane extract of *Periploca angustifolia.*

Figure S12c. PDA chromatogram of dichloromethane extracts of *Periploca angustifolia* at 245 nm.

Figure S12d. PDA chromatogram of dichloromethane of *Periploca angustifolia* at 360 nm

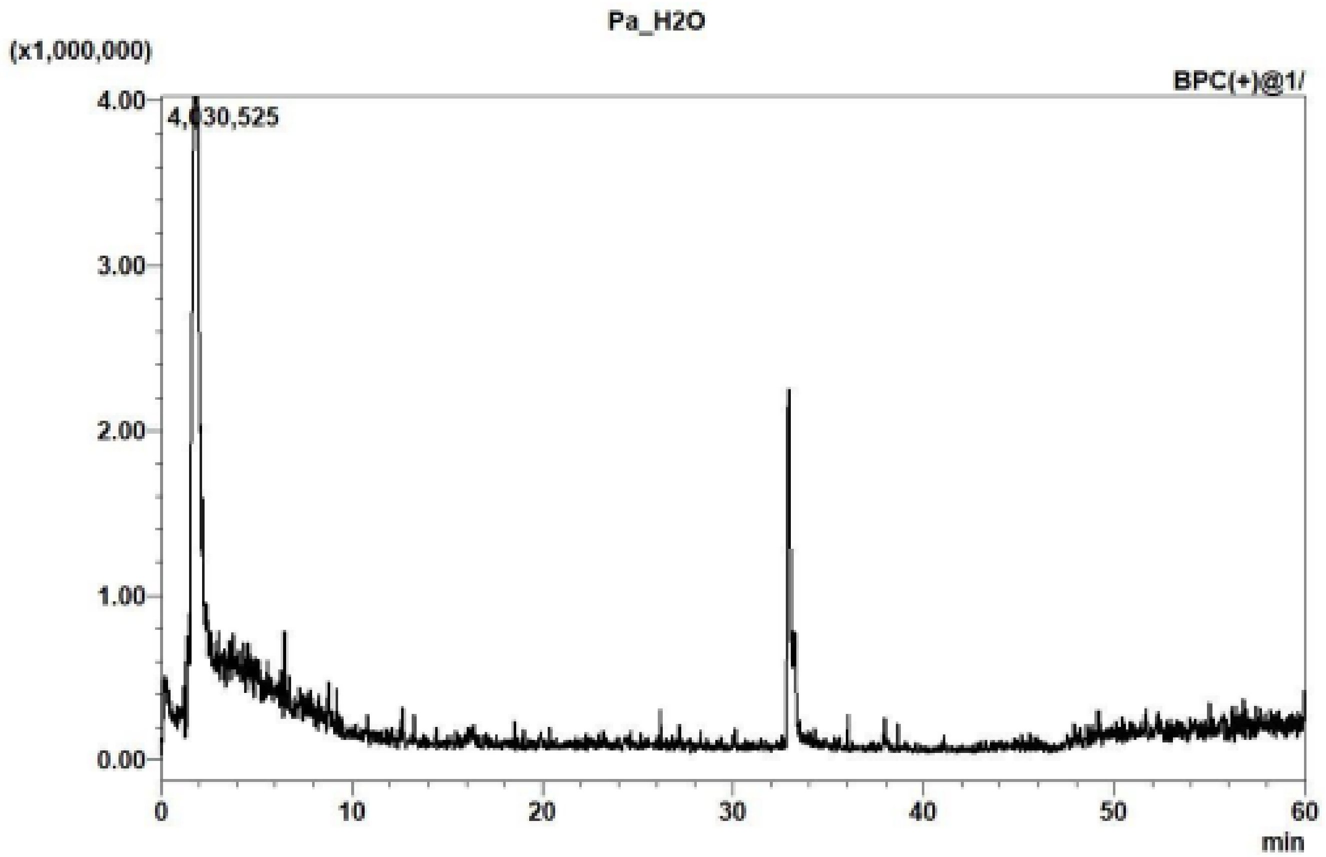

Pa_H2O
(x1,000,000)
BPC(+)@1/
4.00
3.00
2.00
1.00
0.00
0
10
20
30
40
50
60
min

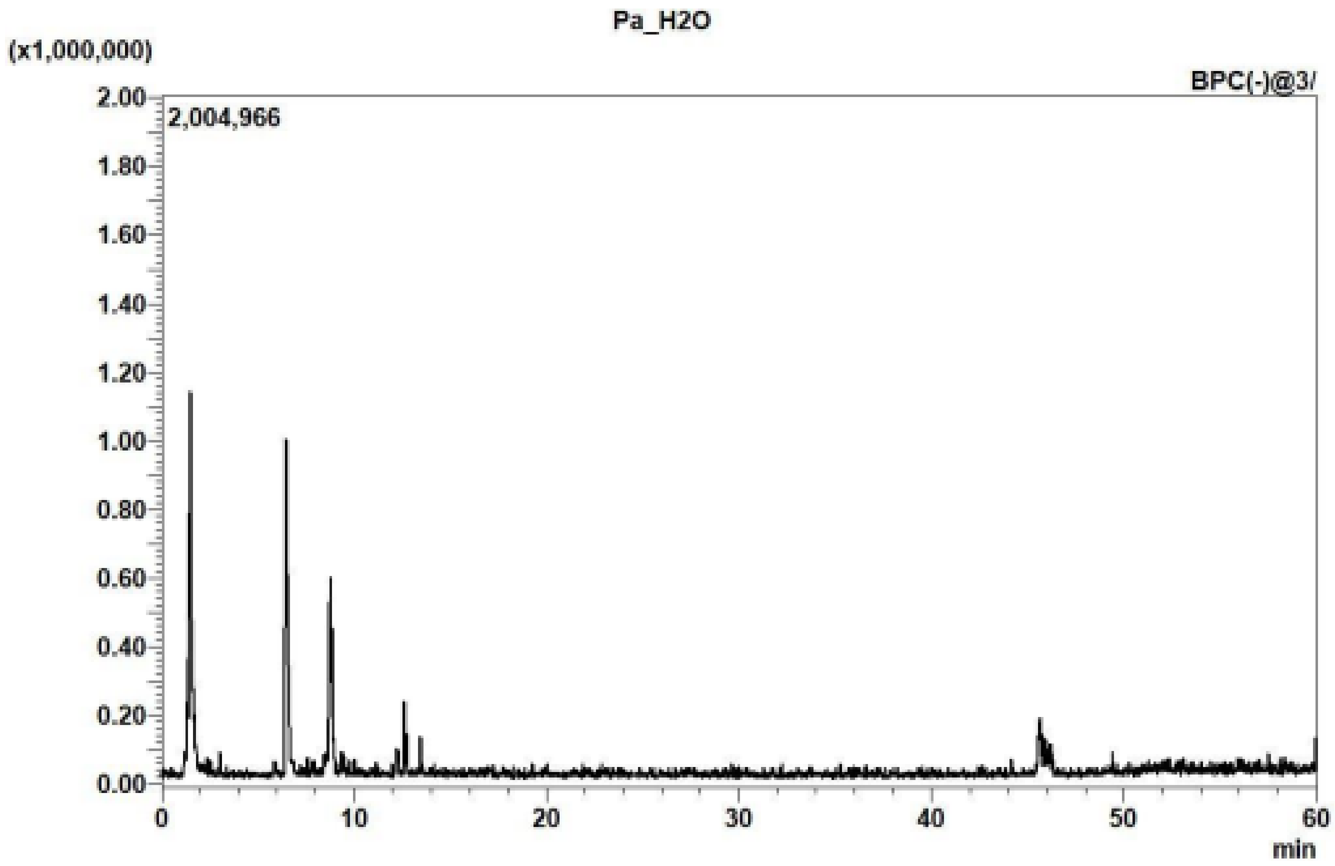

Pa_H2O
(x1,000,000)
BPC(-)@3/
2,004,966
2.00
1.80
1.60
1.40
1.20
1.00
0.80
0.60
0.40
0.20
0.00
0
10
20
30
40
50
60
min

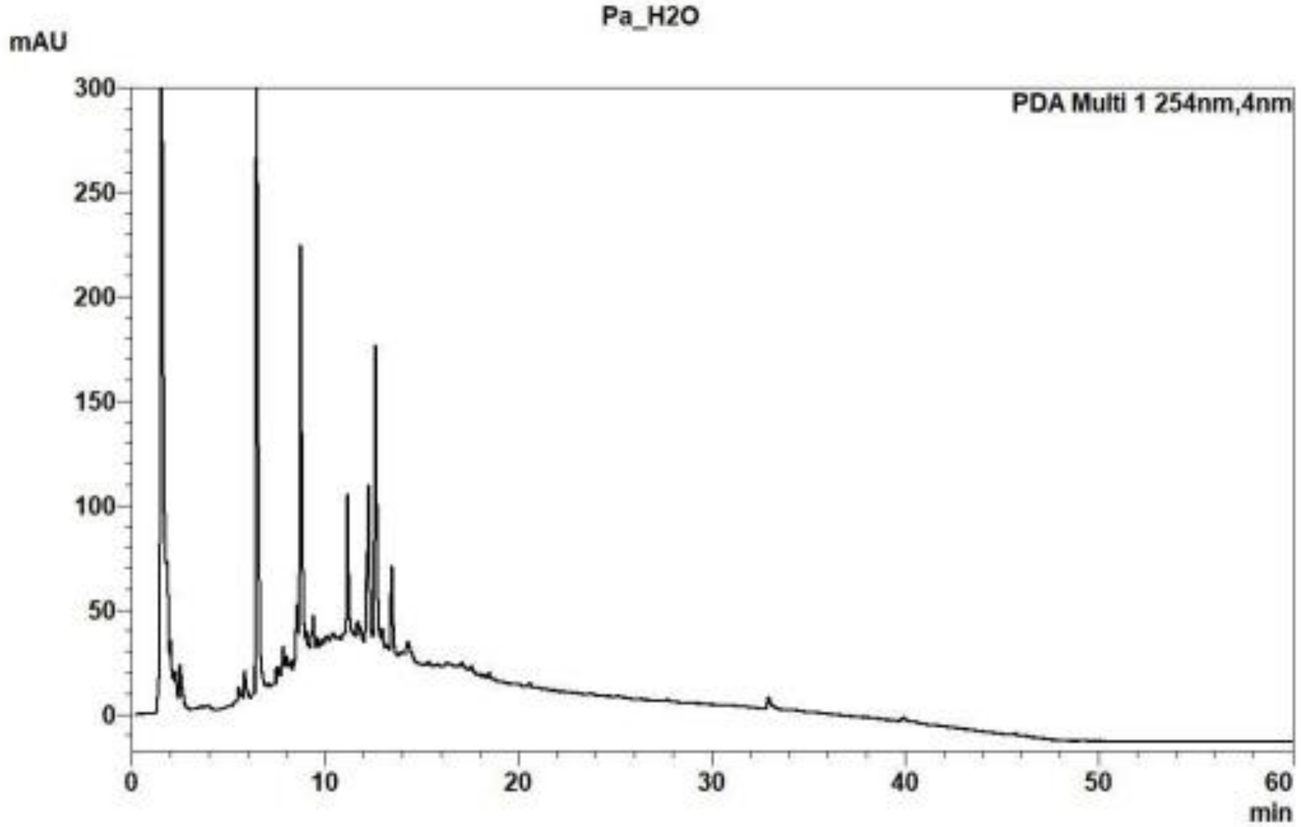
Pa_H2O
mAU
PDA Multi 1 254nm,4nm
300
250
200
150
100
50
0
0
10
20
30
40
50
60
min

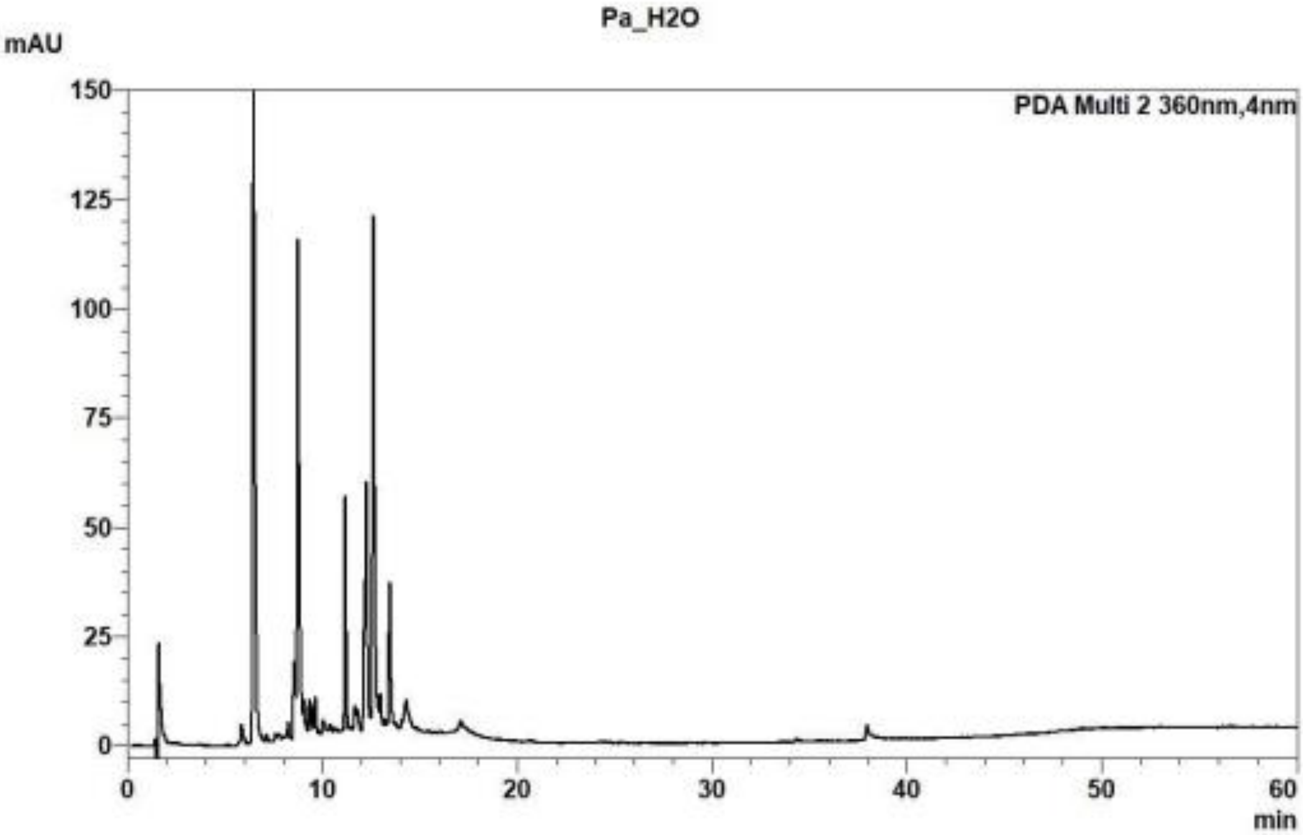
Pa_H2O
mAU
PDA Multi 2 360nm,4nm
150
125
100
75
50
25
0
0
10
20
30
40
50
60
min

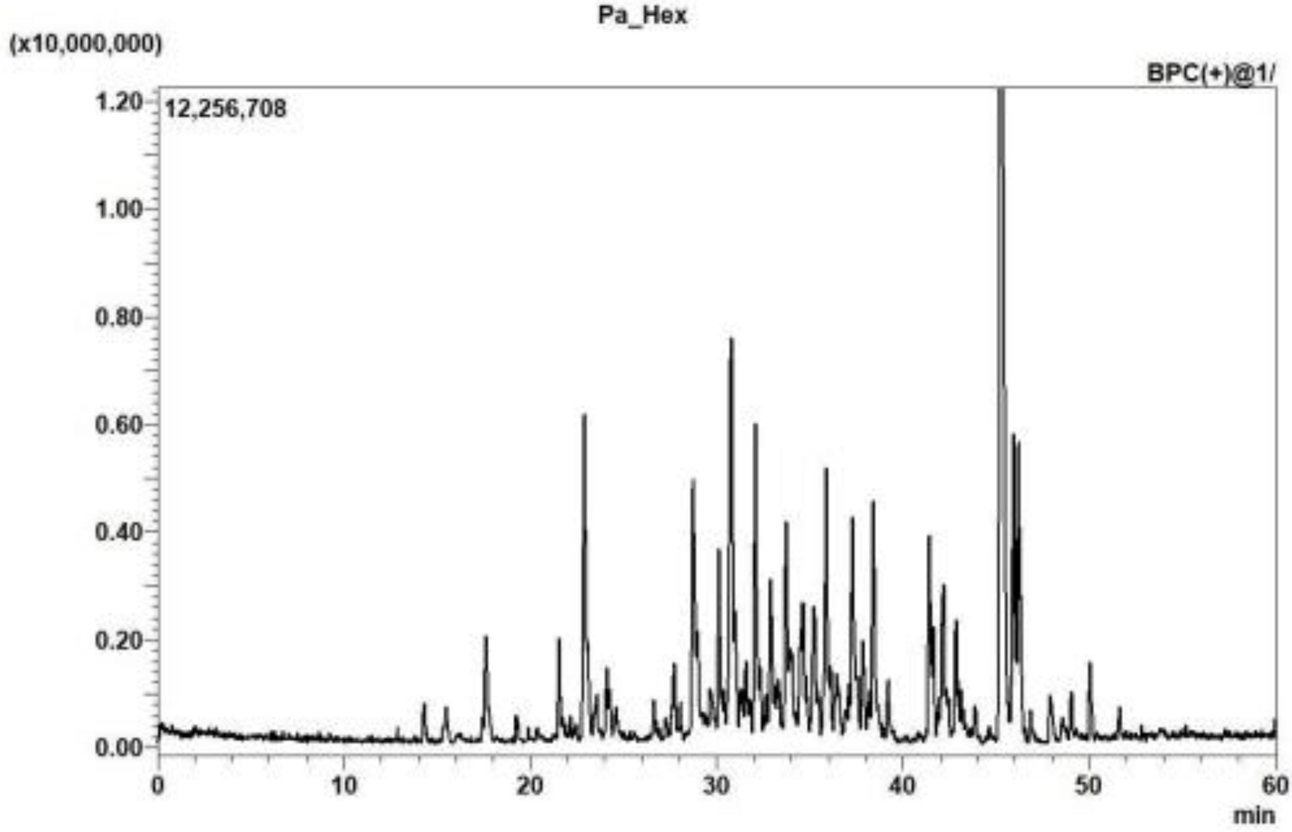

Pa_Hex
(x10,000,000)
BPC(+)@1/
12,256,708
1.20
1.00
0.80
0.60
0.40
0.20
0.00
0
10
20
30
40
50
60
min

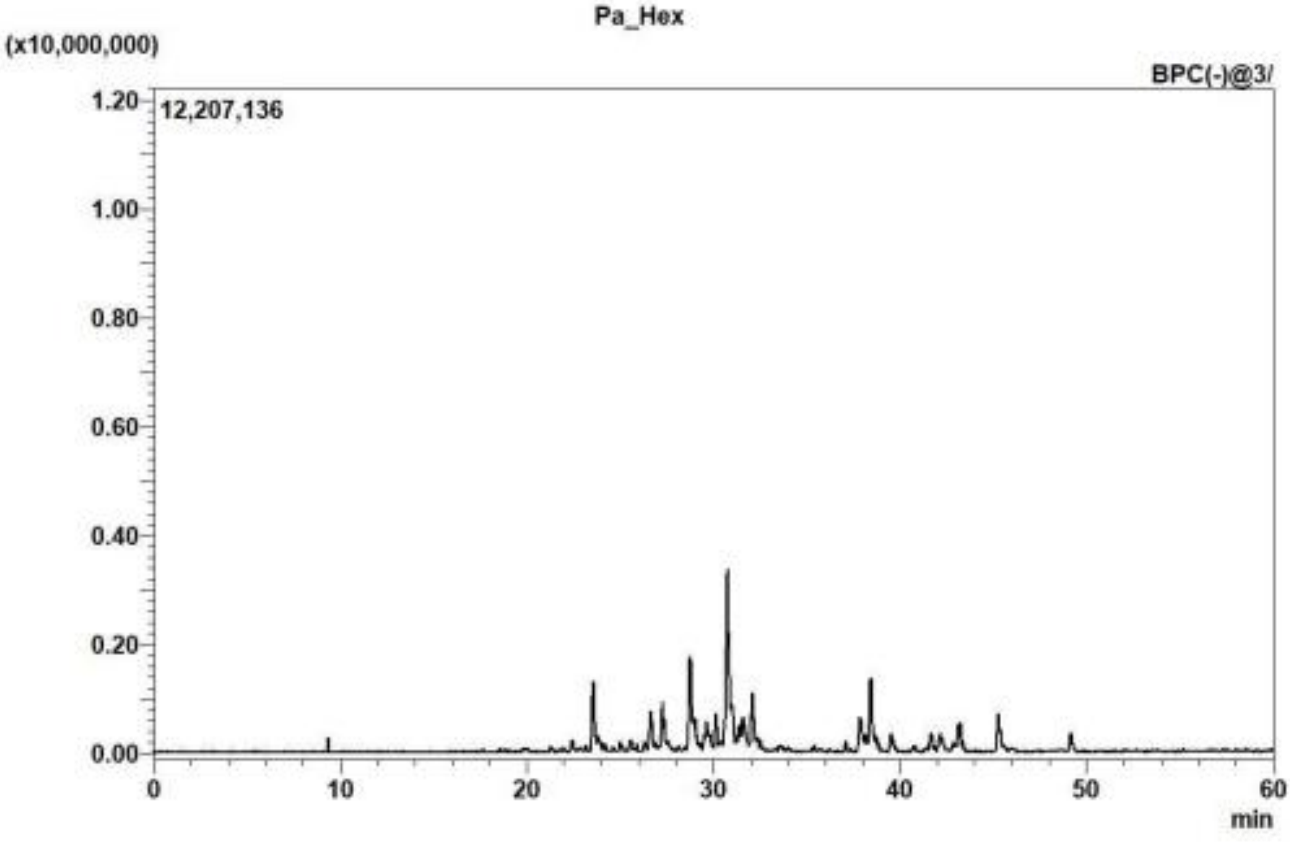

Pa_Hex
(x10,000,000)
BPC(-)@3/
12,207,136
1.20
1.00
0.80
0.60
0.40
0.20
0.00
0
10
20
30
40
50
60
min

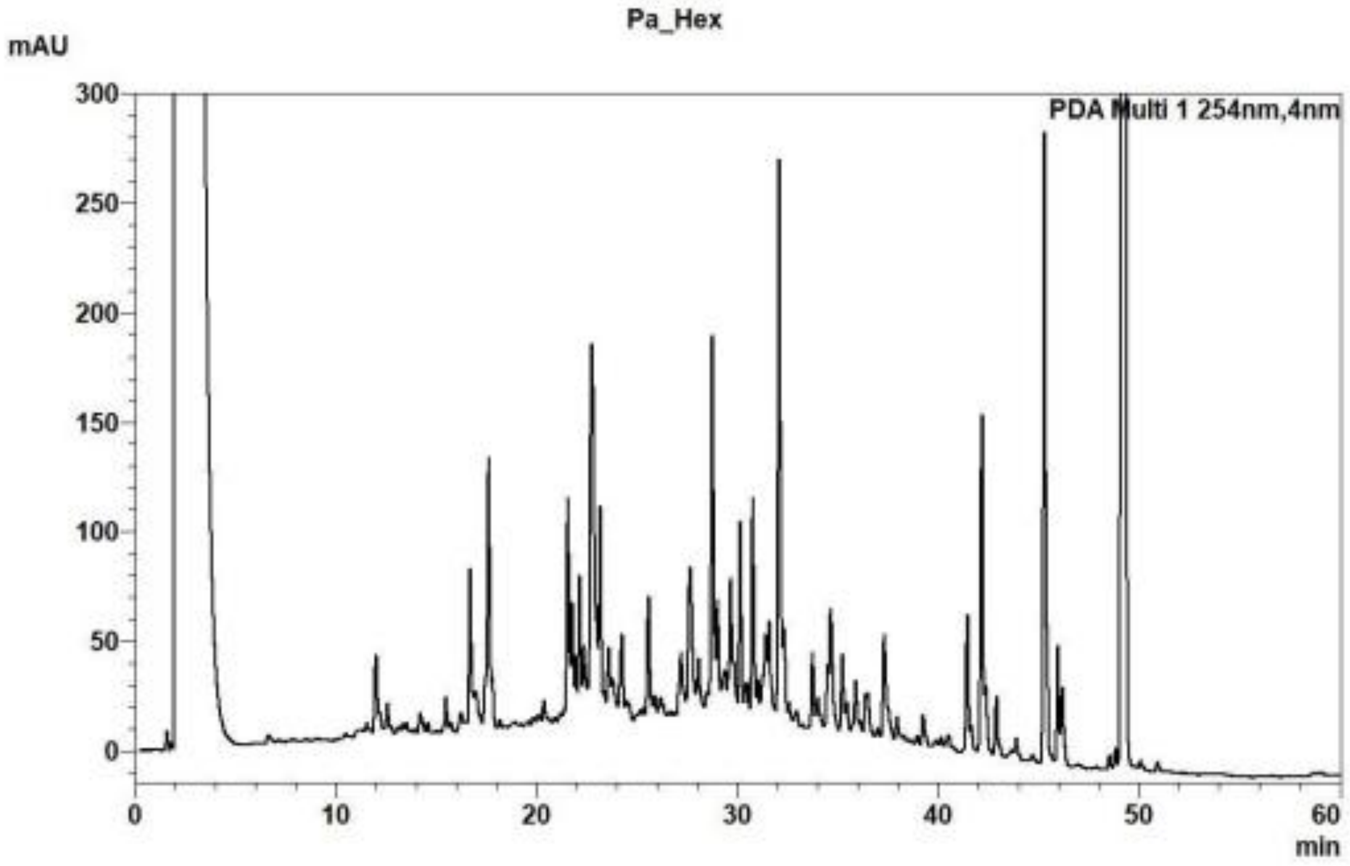
Pa_Hex
mAU
PDA Multi 1 254nm,4nm
300
250
200
150
100
50
0
0
10
20
30
40
50
60
min

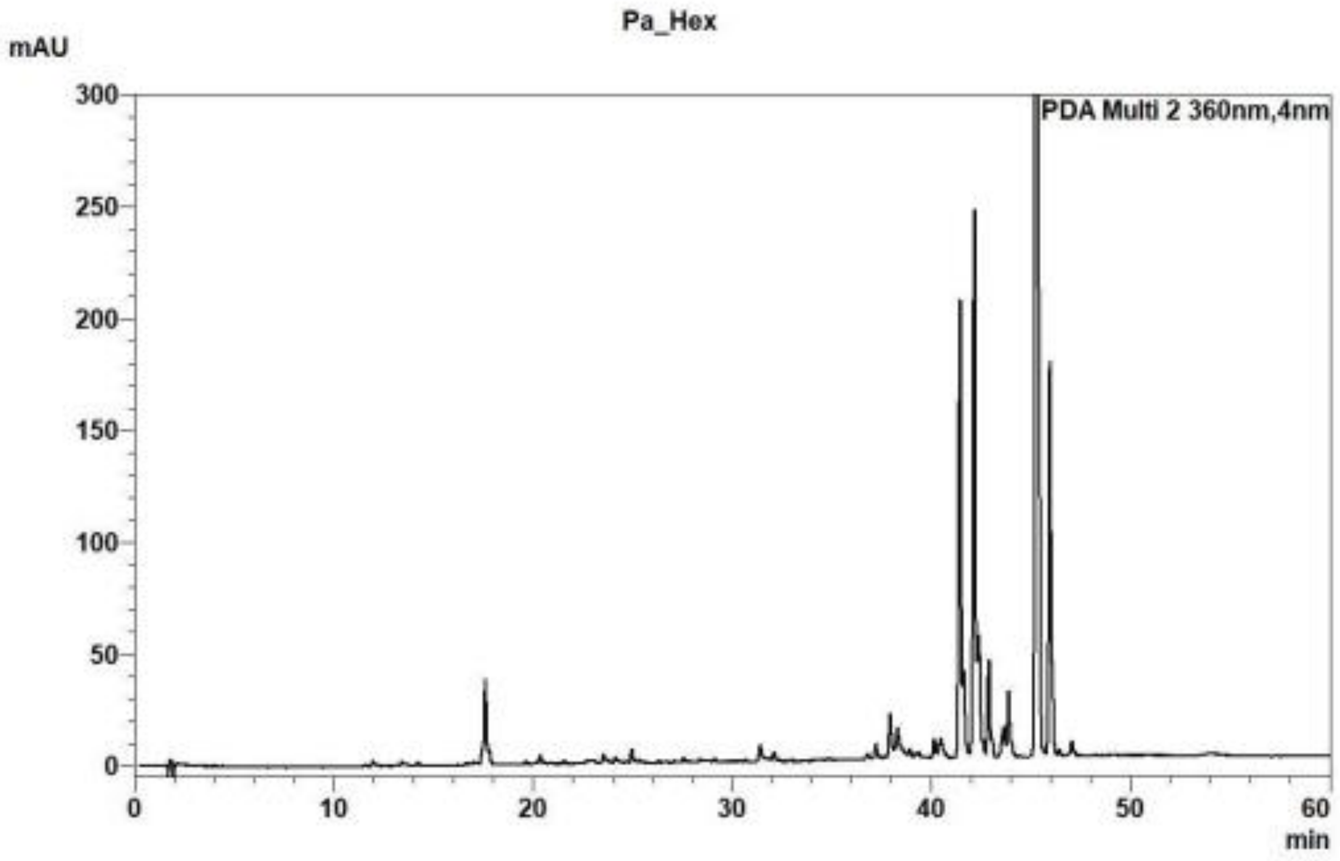
Pa_Hex
mAU
PDA Multi 2 360nm,4nm
300
250
200
150
100
50
0
0
10
20
30
40
50
60
min

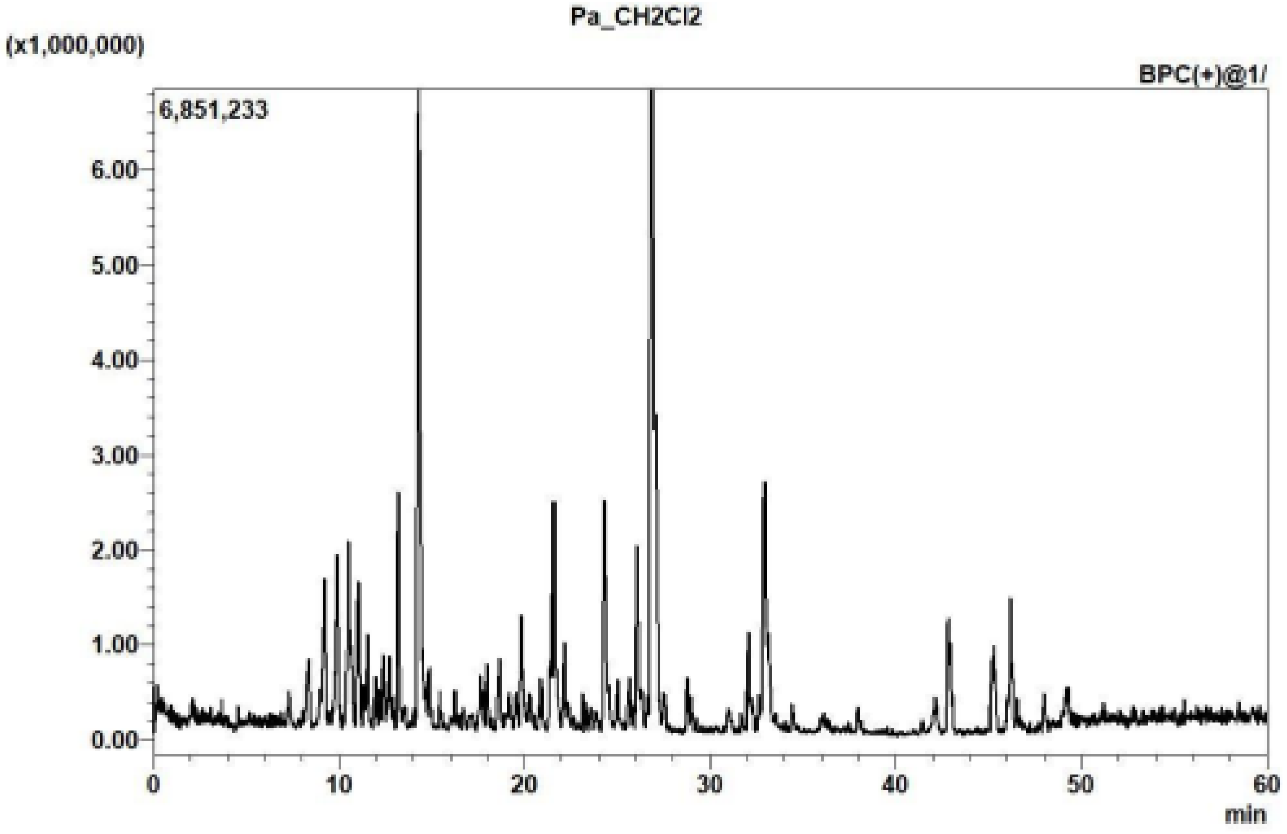
Pa_CH2Cl2
(x1,000,000)
BPC(+)@1/
6,851,233
6.00
5.00
4.00
3.00
2.00
1.00
0.00
0
10
20
30
40
50
60
min

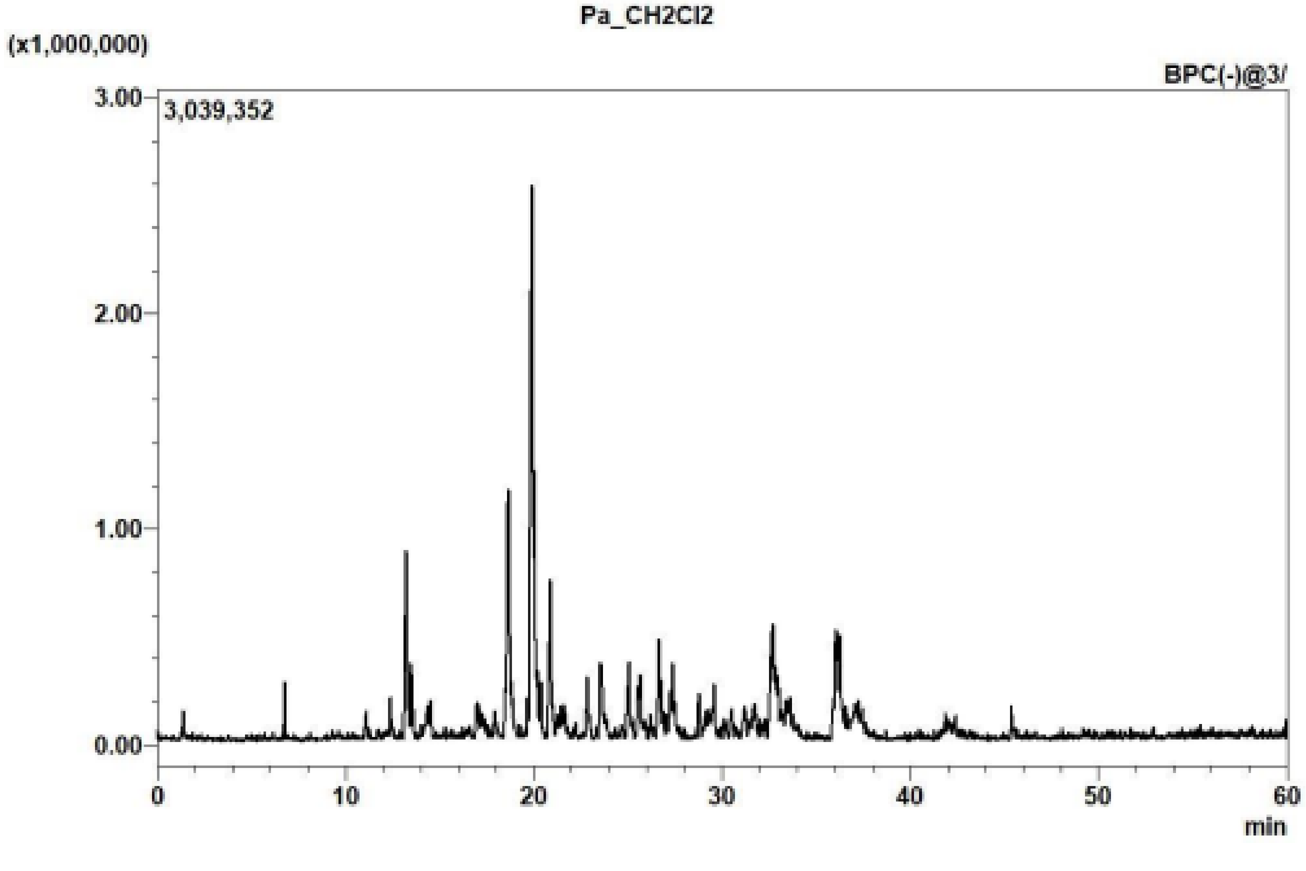
Pa_CH2Cl2
(x1,000,000)
BPC(-)@3/
3.00
3,039,352
2.00
1.00
0.00
0
10
20
30
40
50
60
min

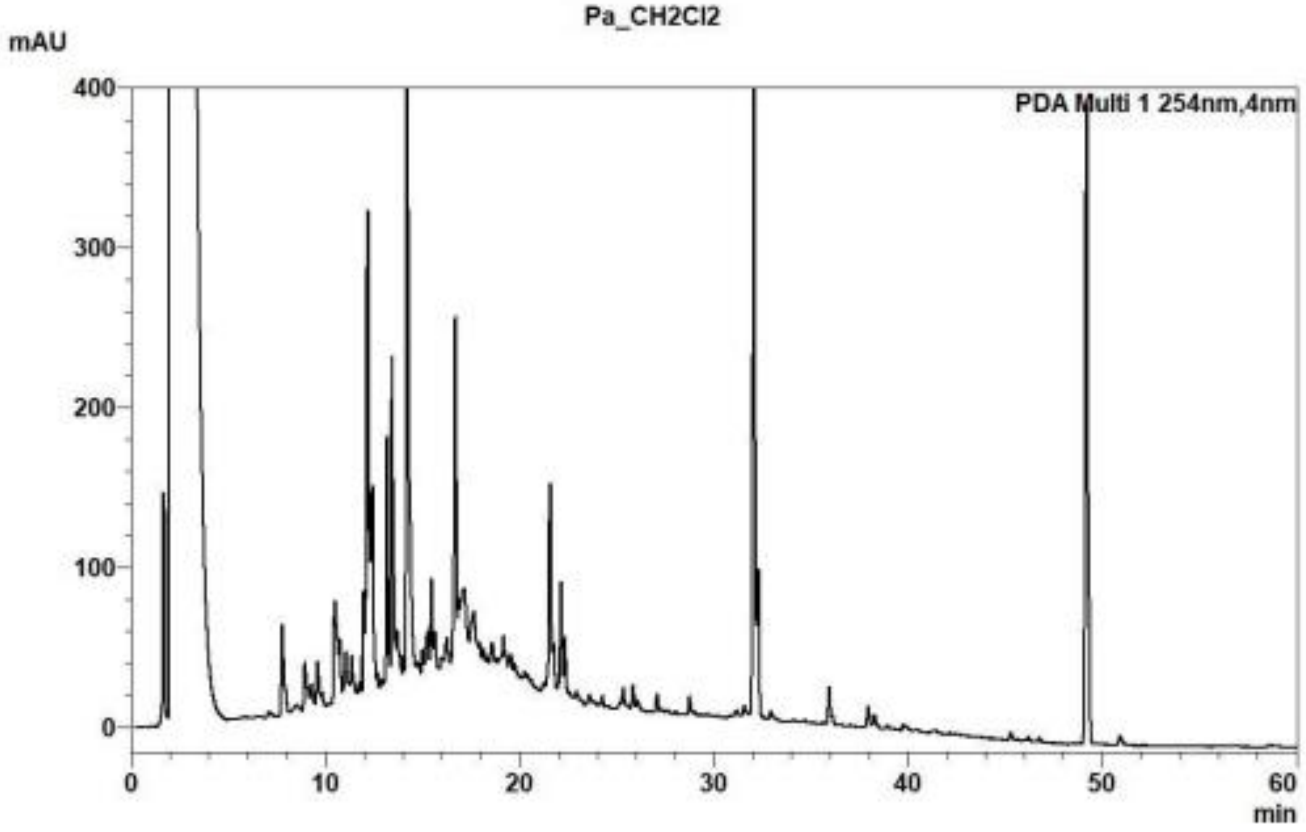
Pa_CH2Cl2
mAU
PDA Multi 1 254nm,4nm
400
300
200
100
0
0
10
20
30
40
50
60
min

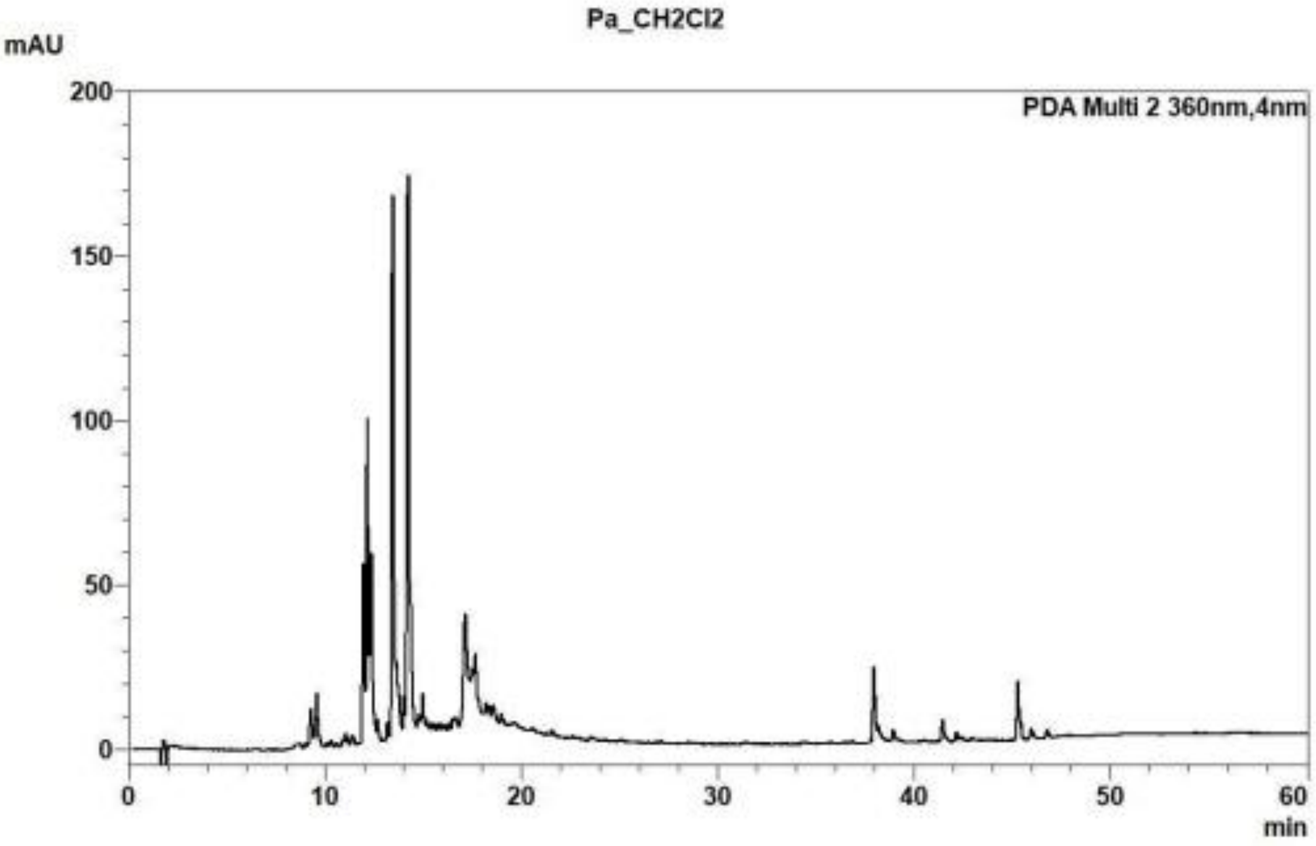
Pa_CH2Cl2
mAU
PDA Multi 2 360nm,4nm
200
150
100
50
0
0
10
20
30
40
50
60
min

Echinops ritro

List of Figures

H_2O phase
Figure S13a. Base Peak Chromatogram (BPC) in the positive ionization mode of water extract of *Echinops ritro*.
Figure S13b. Base Peak Chromatogram (BPC) in the negative ionization mode of water extract of *Echinops ritro*.
Figure S13c. PDA chromatogram of water extracts of *Echinops ritro* at 245 nm.
Figure S13d. PDA chromatogram of water extracts of *Echinops* ritro at 360 nm.
Hexane extract
Figure S14a. Base Peak Chromatogram (BPC) in positive mode of *n*-hexane extract of *Echinops ritro*.
Figure S14b. Base Peak Chromatogram (BPC) in negative mode of *n*-hexane extract of *Echinops ritro*.
Figure S14c. PDA chromatogram of *n*-hexane extracts of *Echinops ritro* at 245 nm.
Figure S14d. PDA chromatogram of *n*-hexane extracts of *Echinops ritro* at 360 nm.
CH2Cl2 extract
Figure S15a. Base Peak Chromatogram (BPC) in positive mode of dichloromethane extract of *Echinops ritro*.
Figure S15b. Base Peak Chromatogram (BPC) in negative mode of dichloromethane extract of *Echinops ritro*.
Figure S15c. PDA chromatogram of dichloromethane extracts of *Echinops ritro* at 245 nm.
Figure S15d. PDA chromatogram of dichloromethane of *Echinops ritro* at 360 nm.

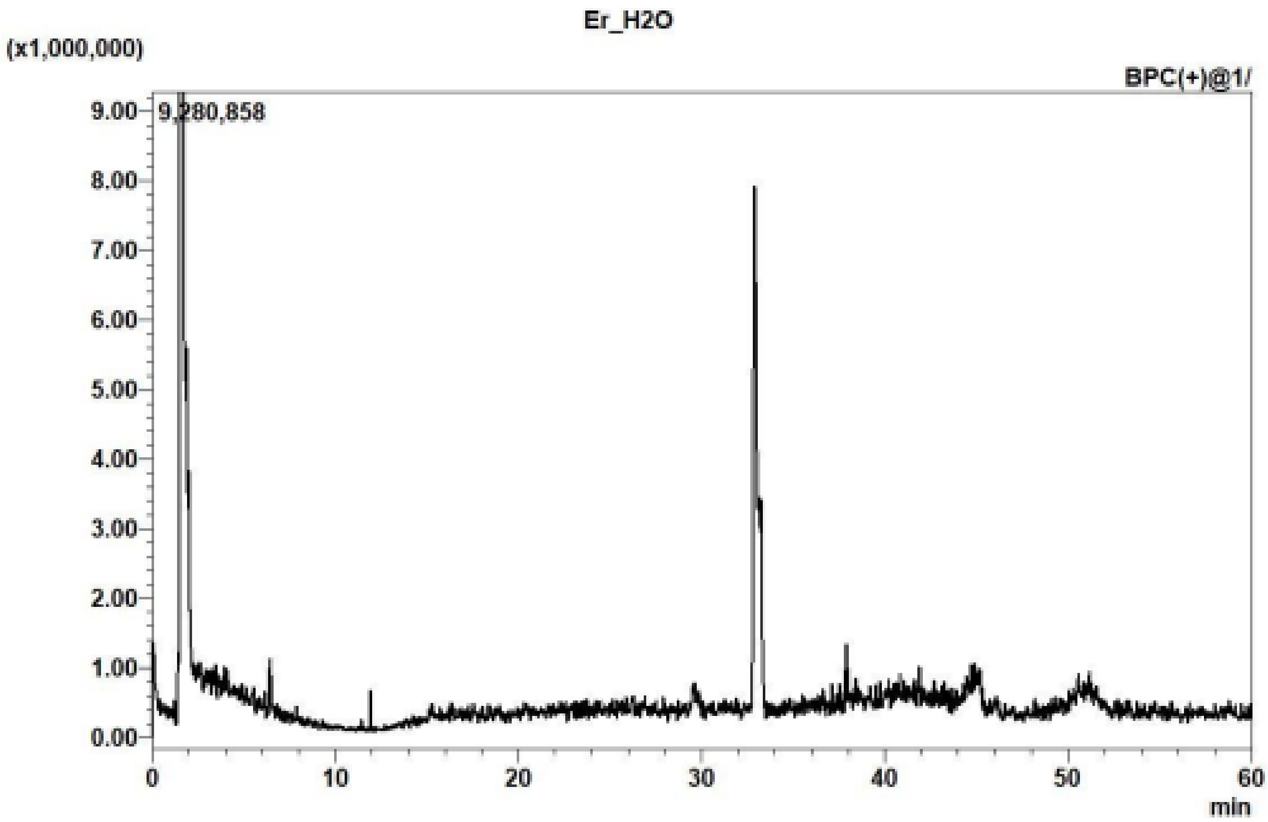

Er_H2O
(x1,000,000)
BPC(+)@1/
9,280,858
9.00
8.00
7.00
6.00
5.00
4.00
3.00
2.00
1.00
0.00
0
10
20
30
40
50
60
min

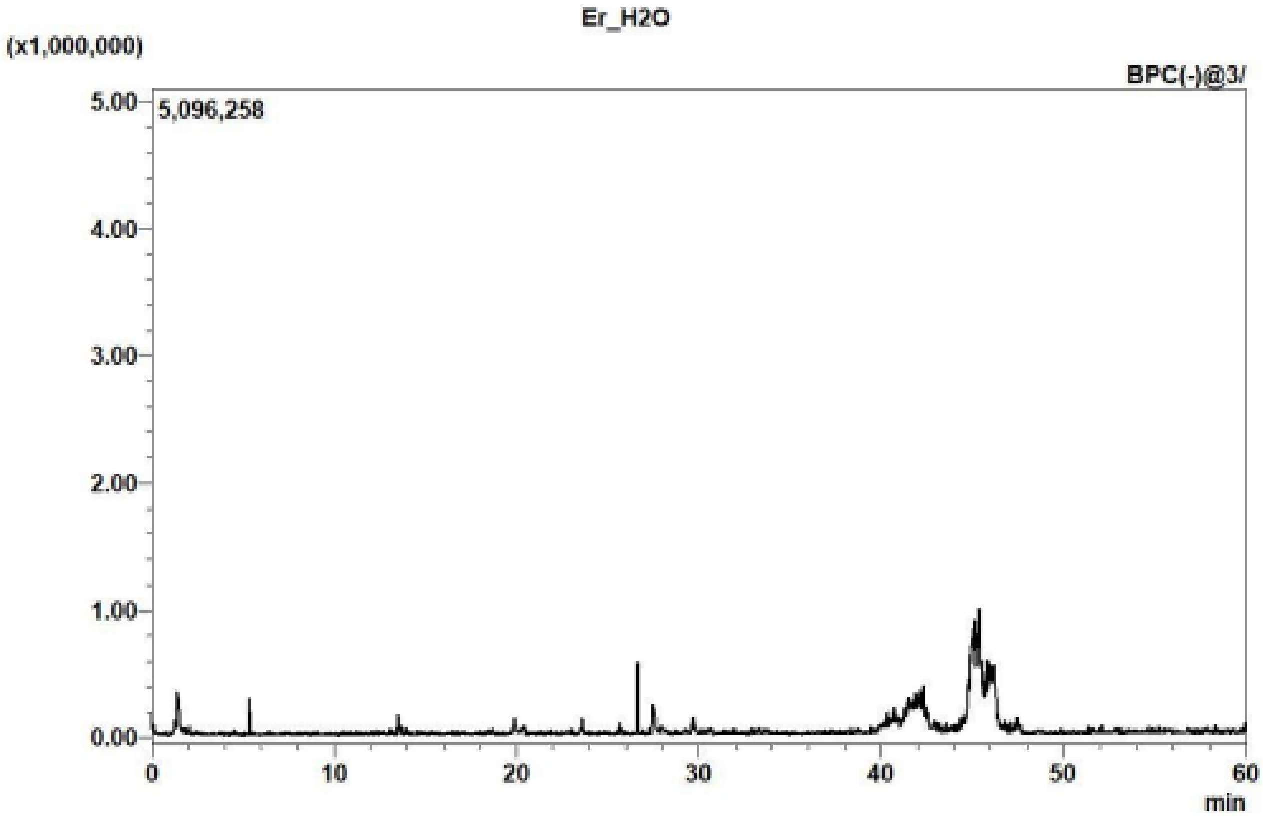

Er_H2O
(x1,000,000)
BPC(-)@3/
5,096,258
5.00
4.00
3.00
2.00
1.00
0.00
0
10
20
30
40
50
60
min

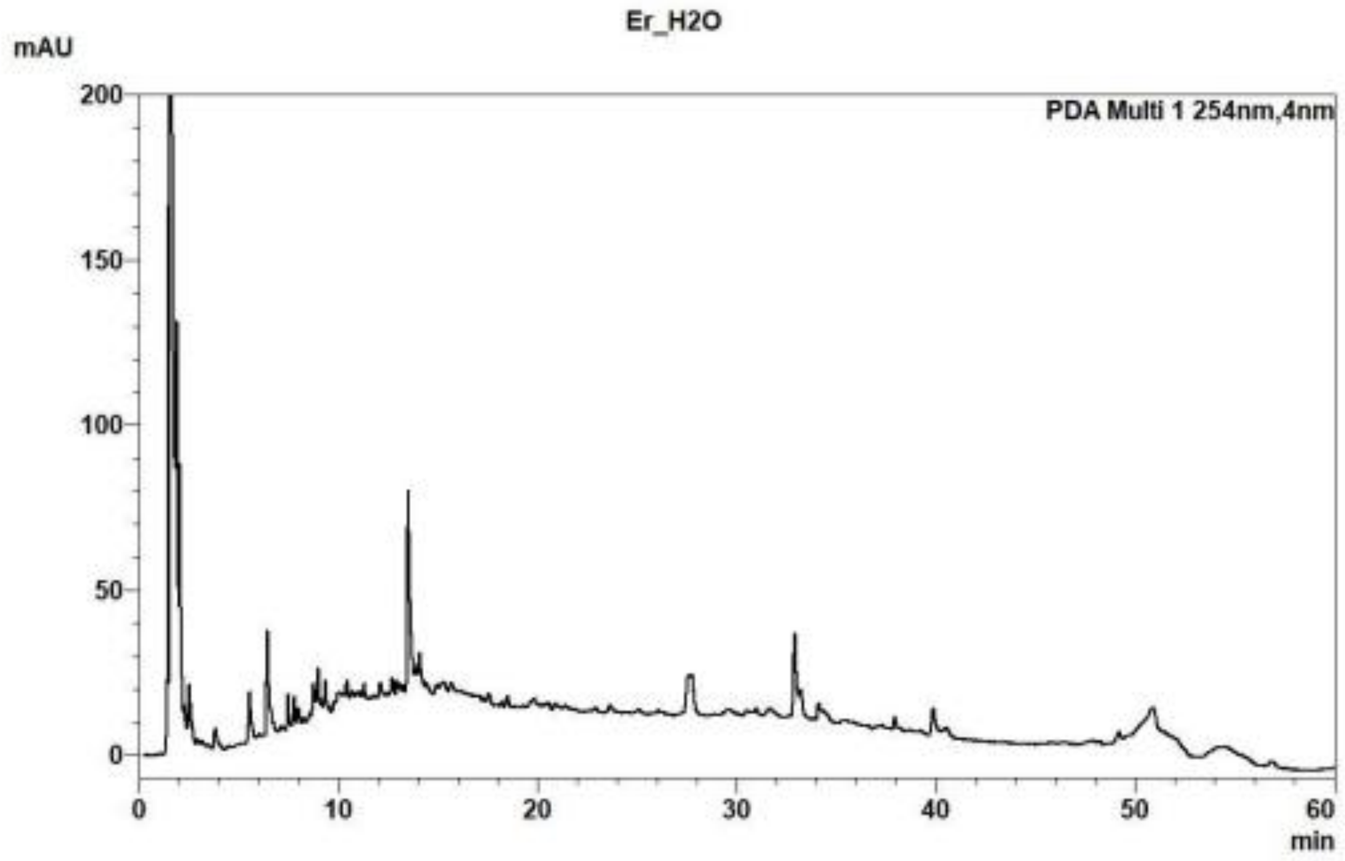
Er_H2O
mAU
PDA Multi 1 254nm,4nm
200
150
100
50
0
0
10
20
30
40
50
60
min

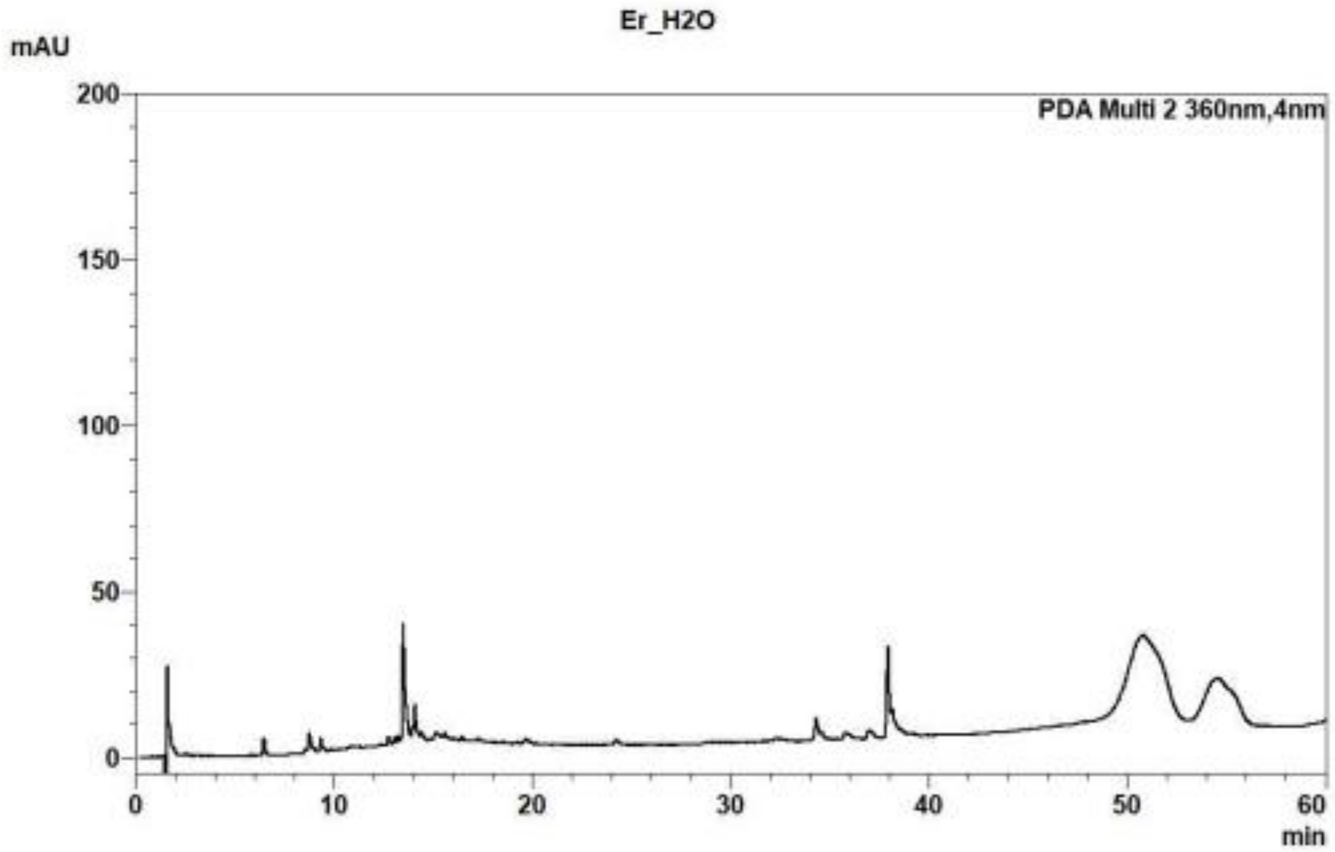
Er_H2O
mAU
PDA Multi 2 360nm,4nm
200
150
100
50
0
0
10
20
30
40
50
60
min

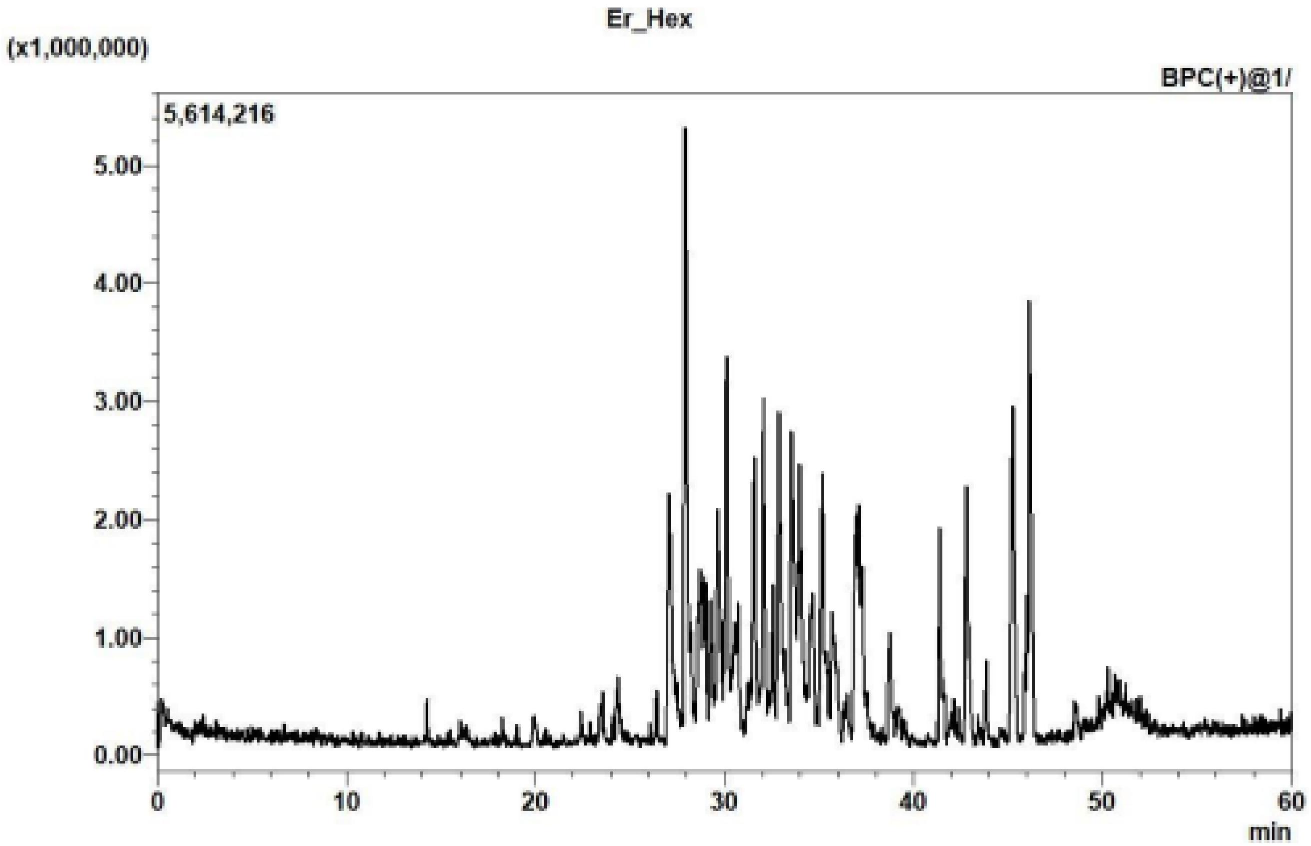
Er_Hex
(x1,000,000)
BPC(+)@1/
5,614,216
5.00
4.00
3.00
2.00
1.00
0.00
0
10
20
30
40
50
60
min

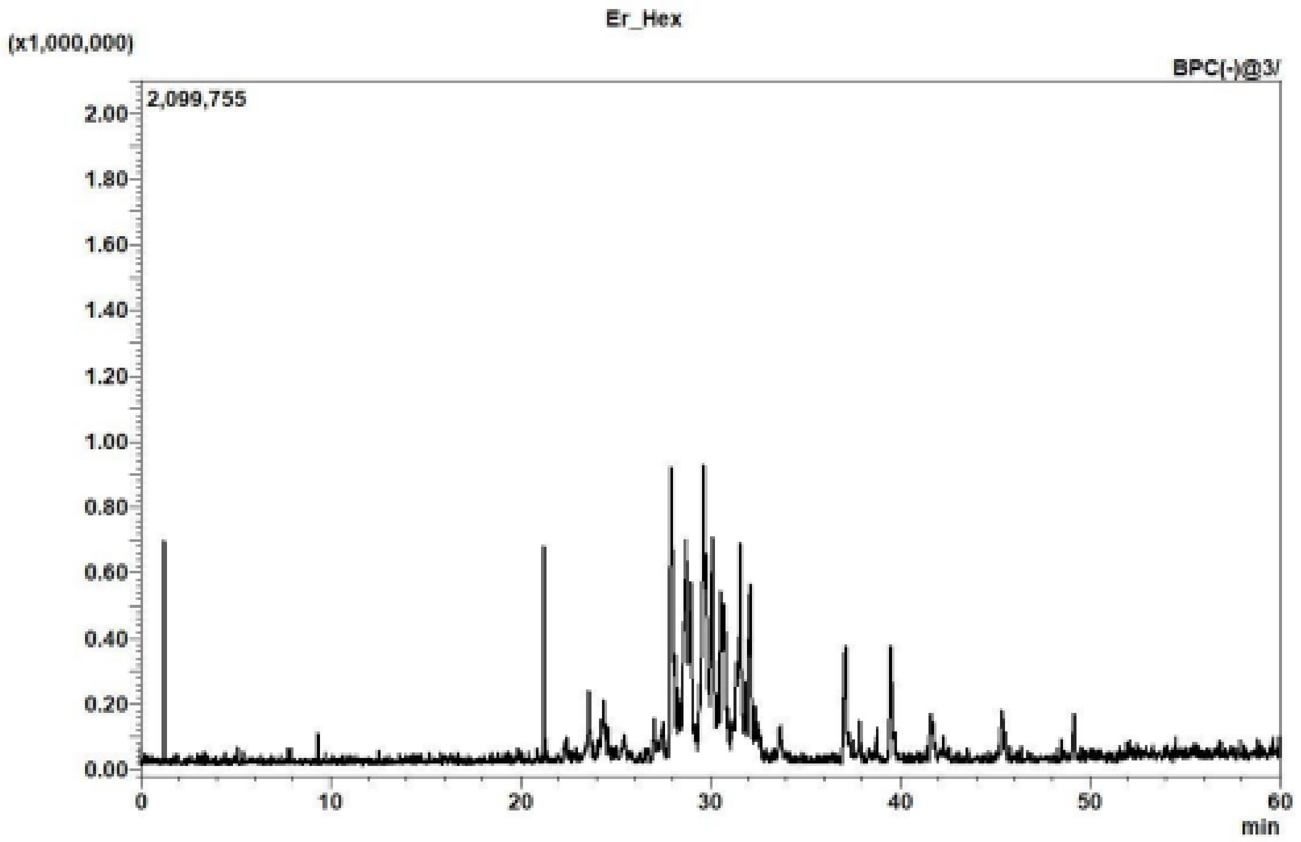
Er_Hex
(x1,000,000)
BPC(-)@3/
2,099,755
2.00
1.80
1.60
1.40
1.20
1.00
0.80
0.60
0.40
0.20
0.00
0
10
20
30
40
50
60
min

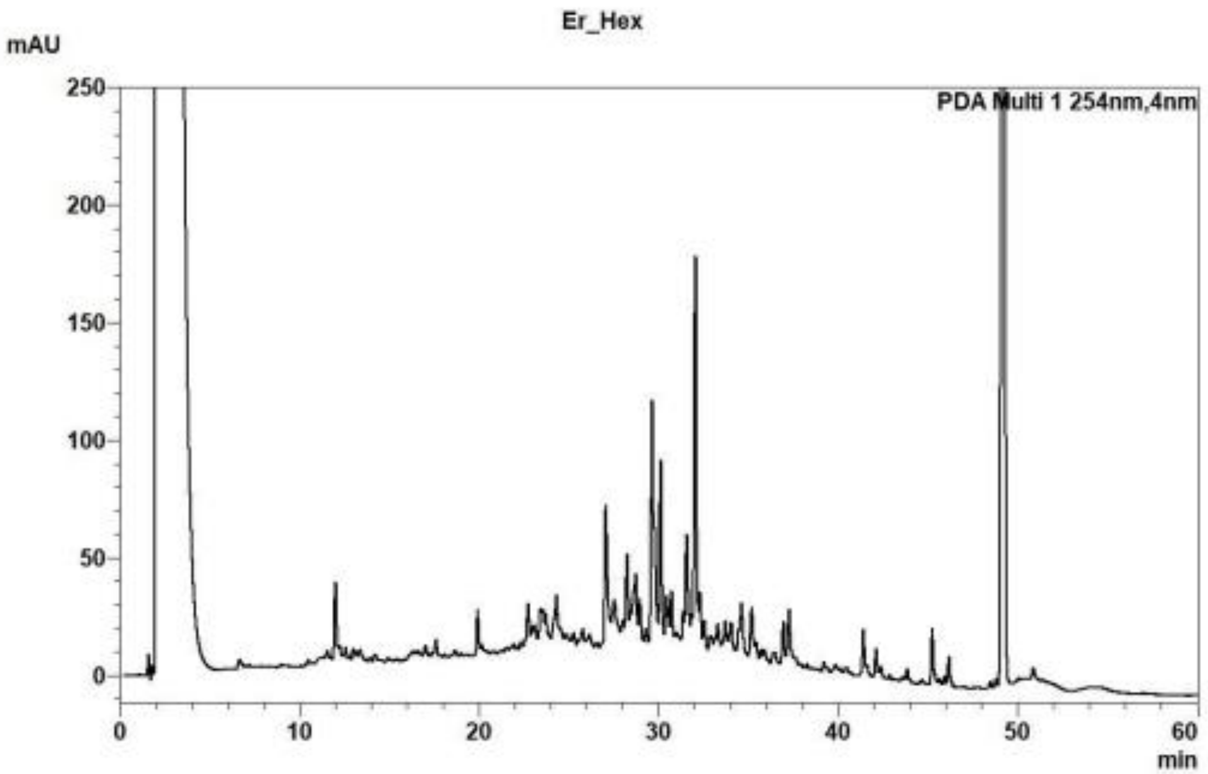
Er_Hex
mAU
PDA Multi 1 254nm,4nm
250
200
150
100
50
0
0
10
20
30
40
50
60
min

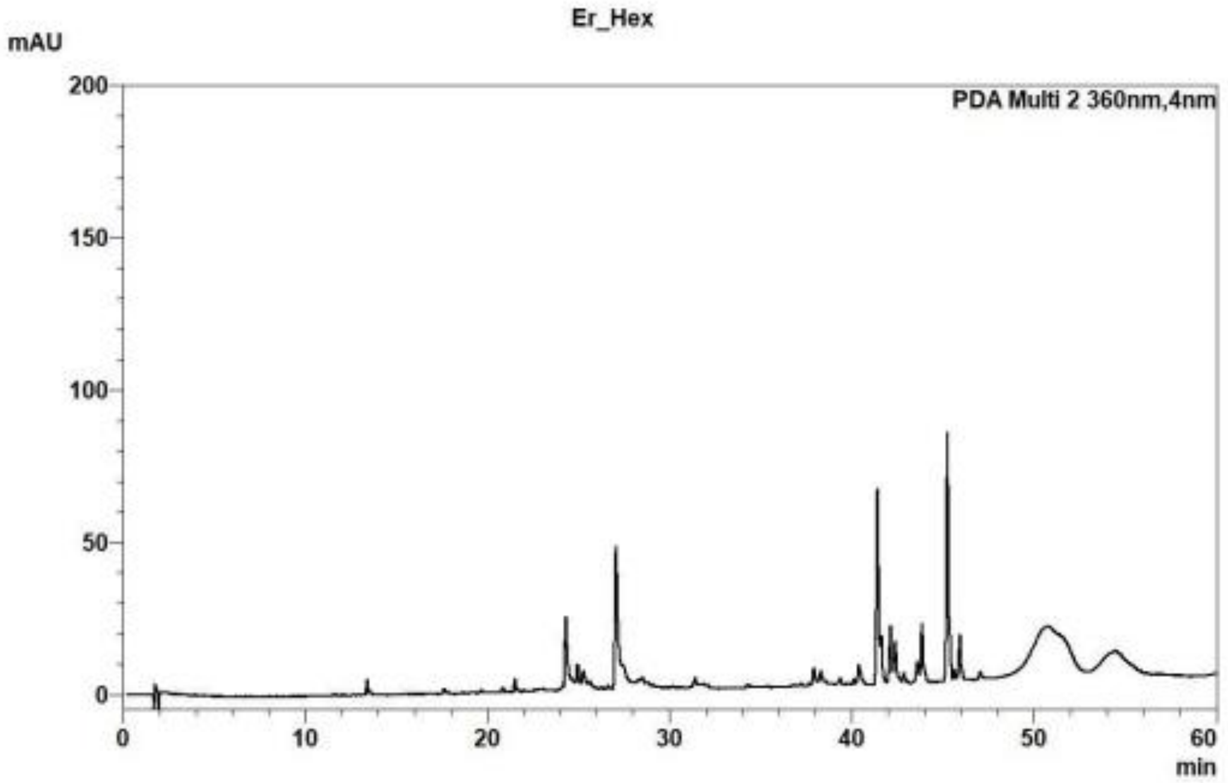
Er_Hex
mAU
PDA Multi 2 360nm,4nm
200
150
100
50
0
0
10
20
30
40
50
60
min

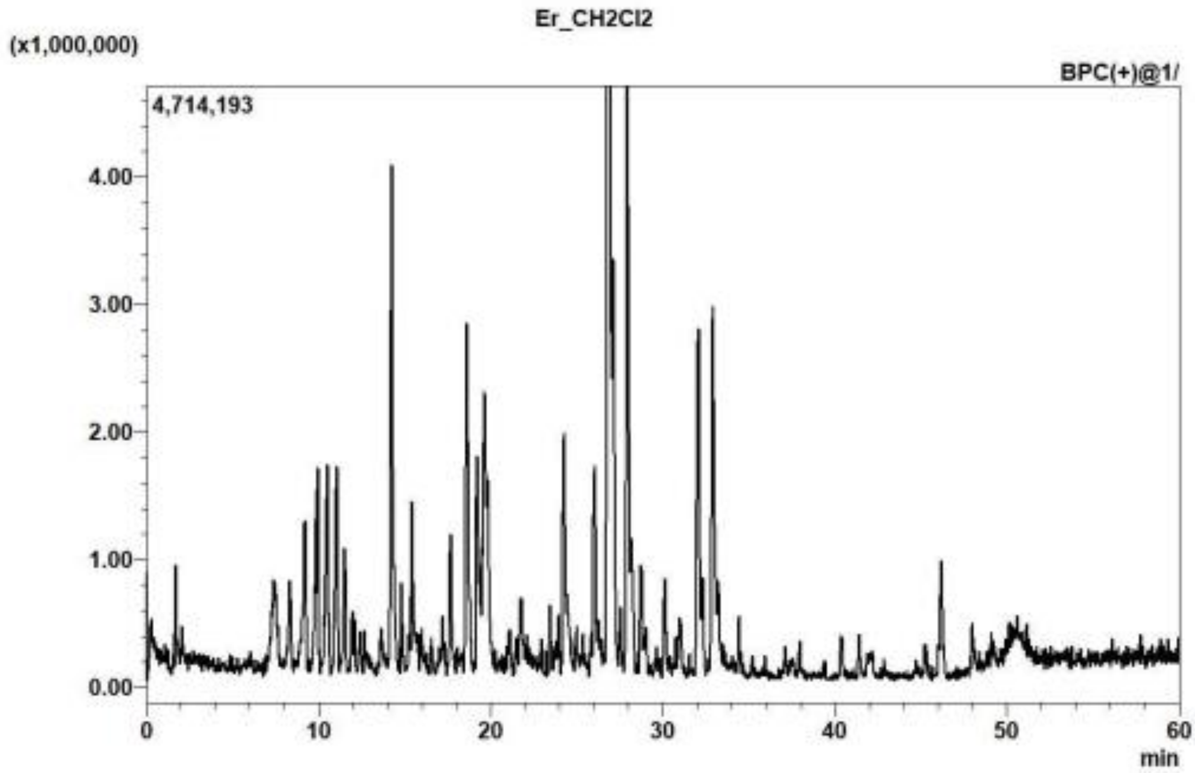
Er_CH2Cl2
(x1,000,000)
BPC(+)@1/
4,714,193
4.00
3.00
2.00
1.00
0.00
0
10
20
30
40
50
60
min

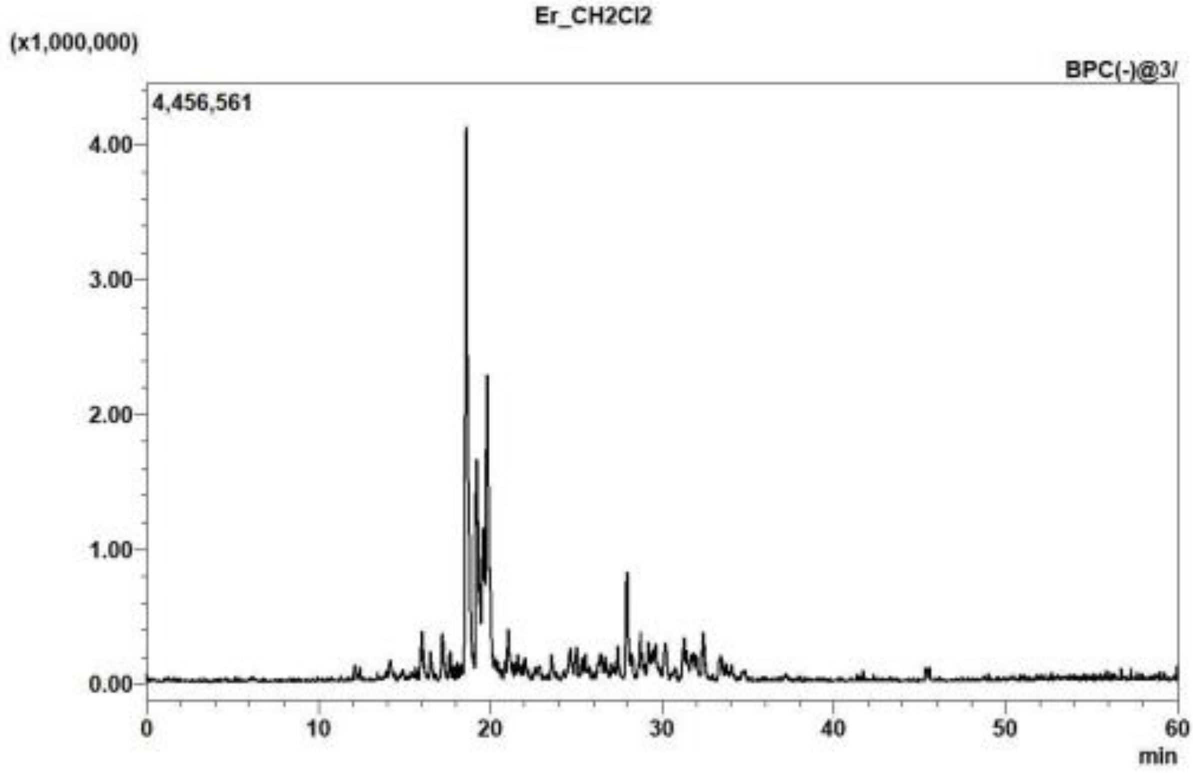
Er_CH2Cl2
(x1,000,000)
BPC(-)@3/
4,456,561
4.00
3.00
2.00
1.00
0.00
0
10
20
30
40
50
60
min

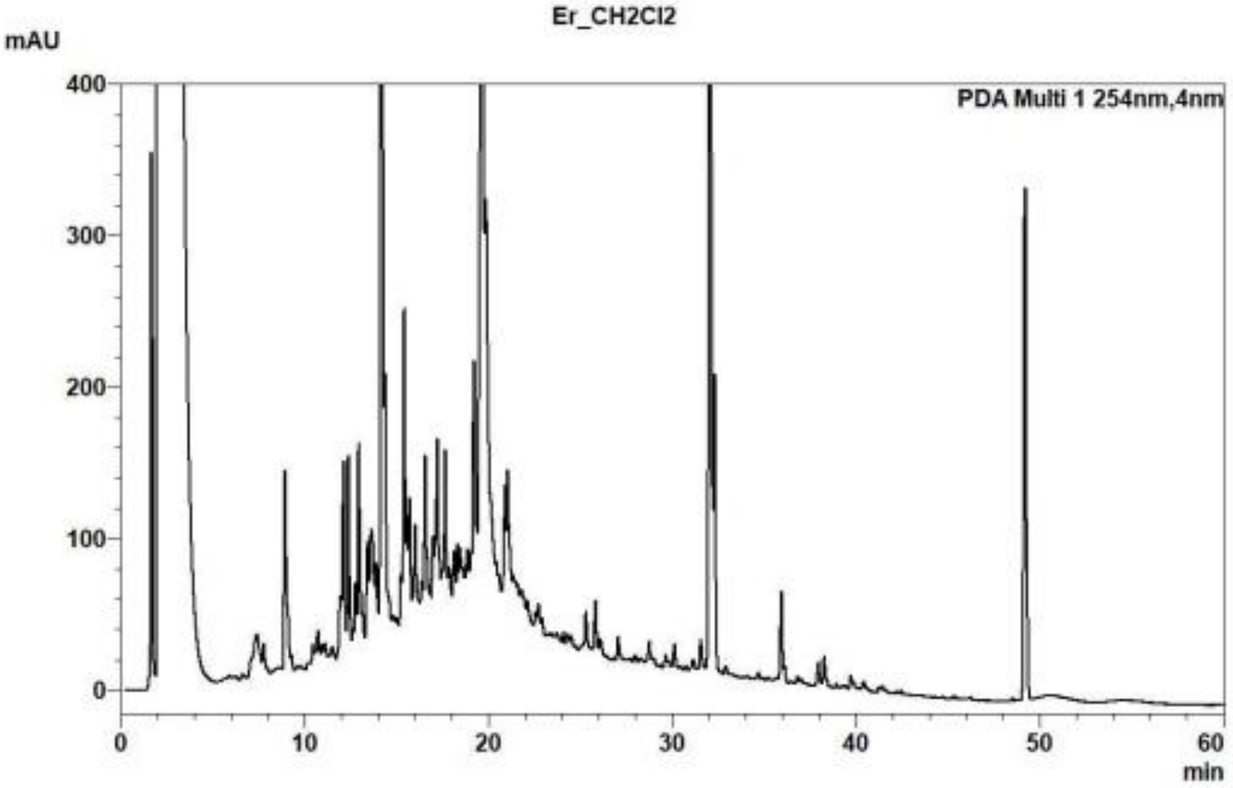
Er_CH2Cl2
mAU
PDA Multi 1 254nm,4nm
400
300
200
100
0
0
10
20
30
40
50
60
min

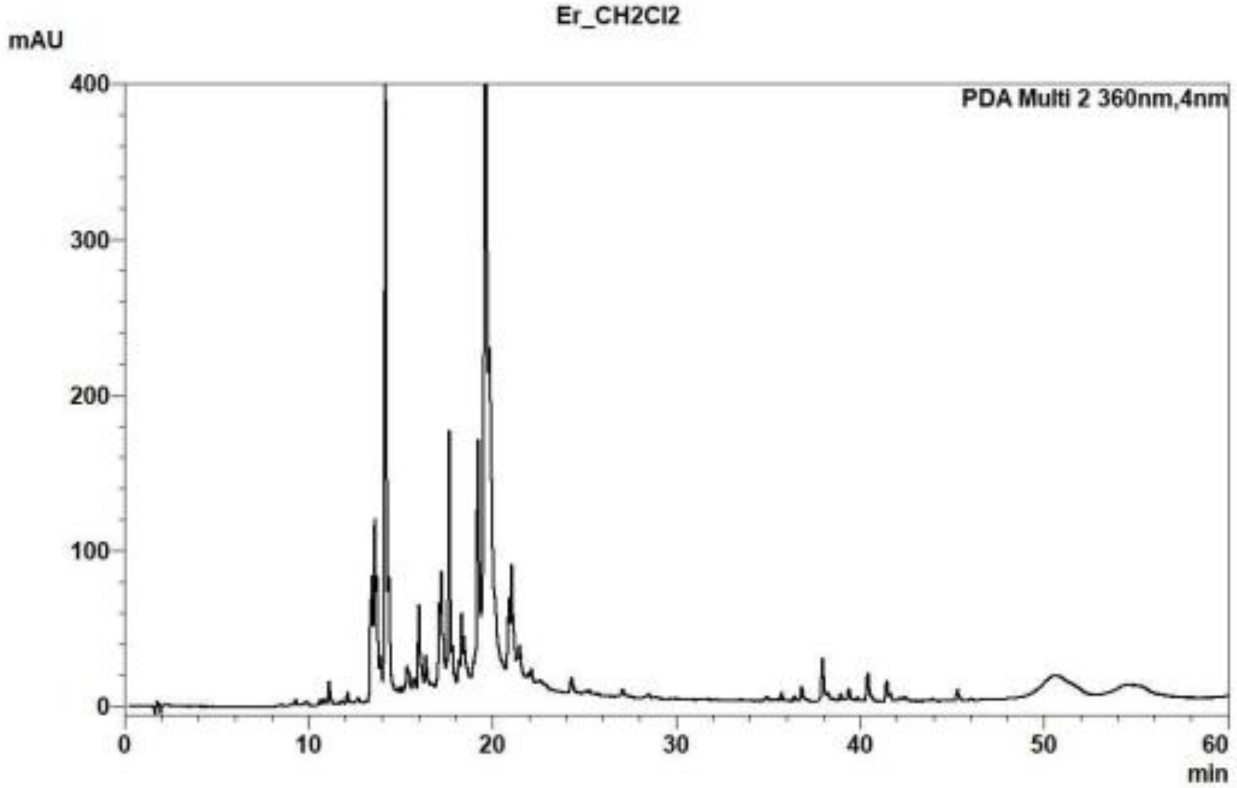
Er_CH2Cl2
mAU
PDA Multi 2 360nm,4nm
400
300
200
100
0
0
10
20
30
40
50
60
min

Prasium majus

List of Figures

H_2O phase
Figure S16a. Base Peak Chromatogram (BPC) in the positive ionization mode of water extract of *Prasium majus*.
Figure S16b. Base Peak Chromatogram (BPC) in the negative ionization mode of water extract of *Prasium majus*.
Figure S16c. PDA chromatogram of water extracts of *Prasium majus* at 245 nm.
Figure S16d. PDA chromatogram of water extracts of *Prasium majus* at 360 nm.
Hexane extract
Figure S17a. Base Peak Chromatogram (BPC) in positive mode of *n*-hexane extract of *Prasium majus*.
Figure S17b. Base Peak Chromatogram (BPC) in negative mode of *n*-hexane extract of *Prasium majus*.
Figure S17c. PDA chromatogram of *n*-hexane extracts of *Prasium majus* at 245 nm.
Figure S17d. PDA chromatogram of *n*-hexane extracts of *Prasium majus* at 360 nm.
CH2Cl2 extract
Figure S18a. Base Peak Chromatogram (BPC) in positive mode of dichloromethane extract of *Prasium majus*.
Figure S18b. Base Peak Chromatogram (BPC) in negative mode of dichloromethane extract of *Prasium majus*.
Figure S18c. PDA chromatogram of dichloromethane extracts of *Prasium majus* at 245 nm.
Figure S18d. PDA chromatogram of dichloromethane of *Prasium majus* at 360 nm.

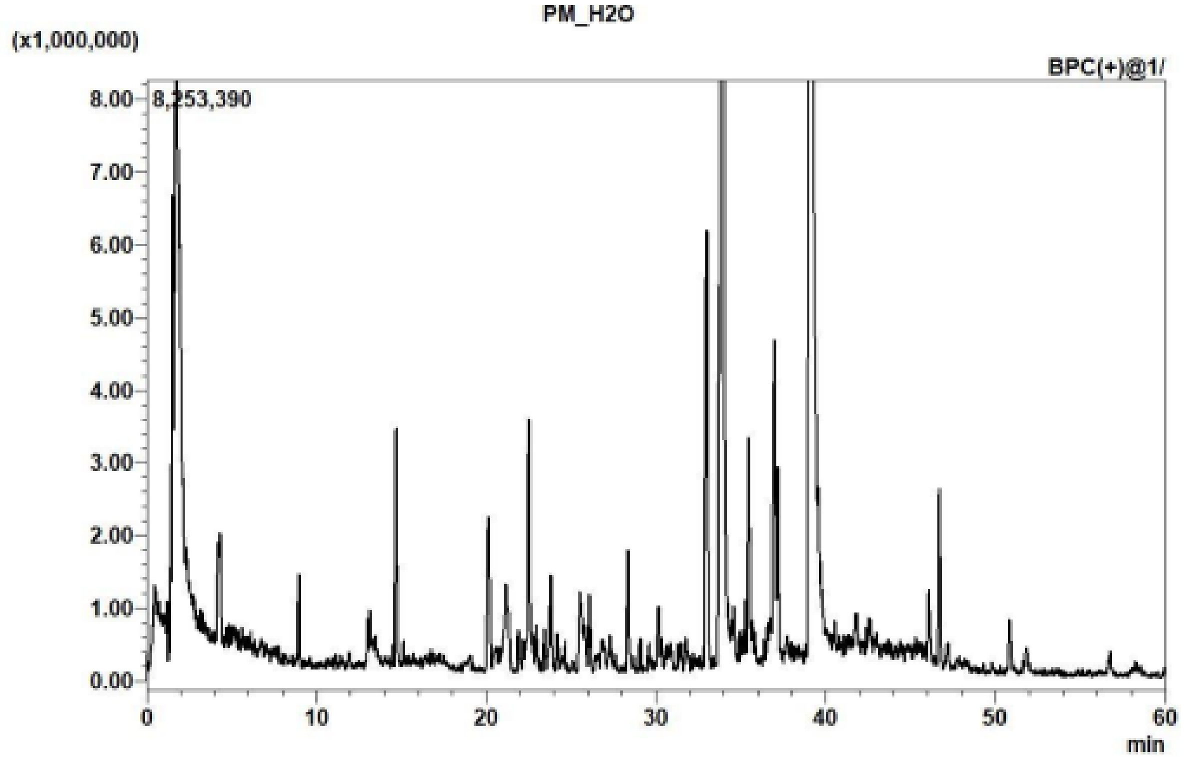
PM_H2O
(x1,000,000)
BPC(+)@1/
8,253,390
8.00
7.00
6.00
5.00
4.00
3.00
2.00
1.00
0.00
0
10
20
30
40
50
60
min

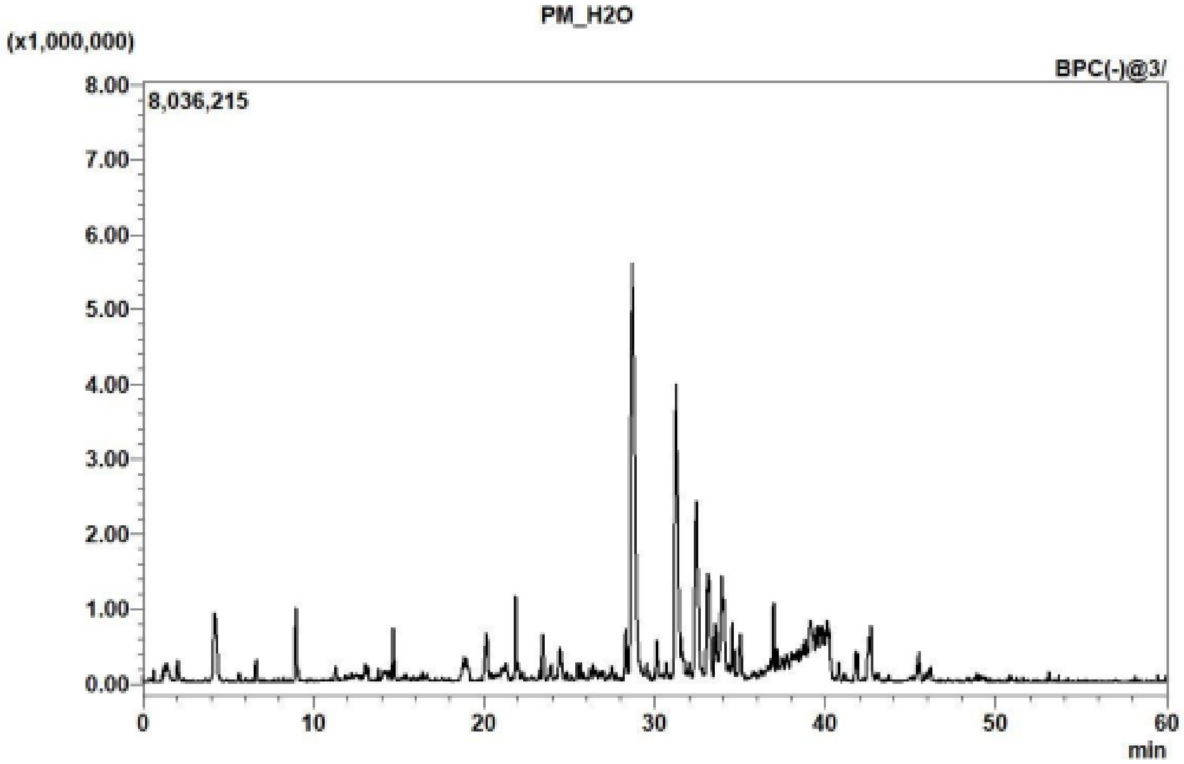
PM_H2O
(x1,000,000)
BPC(-)@3/
8,036,215
8.00
7.00
6.00
5.00
4.00
3.00
2.00
1.00
0.00
0
10
20
30
40
50
60
min

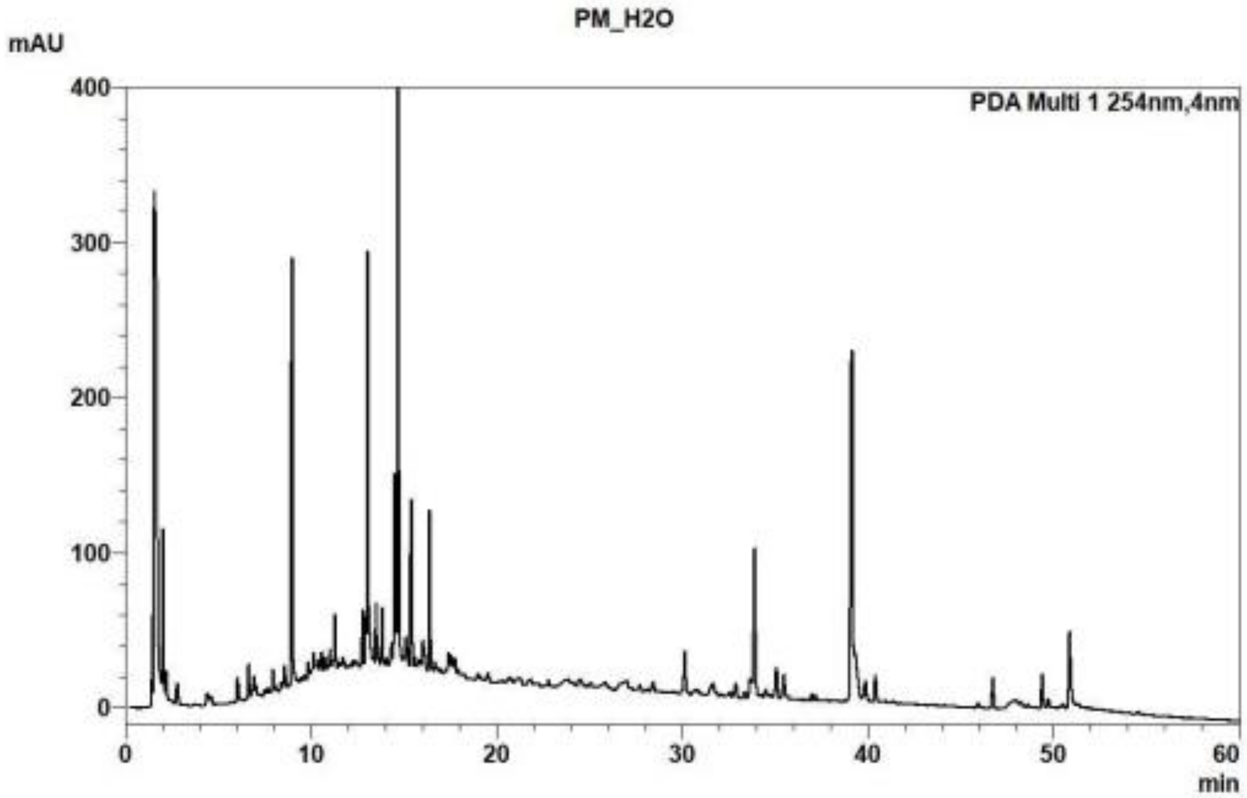
PM_H2O
mAU
PDA Multi 1 254nm,4nm
400
300
200
100
0
0
10
20
30
40
50
60
min

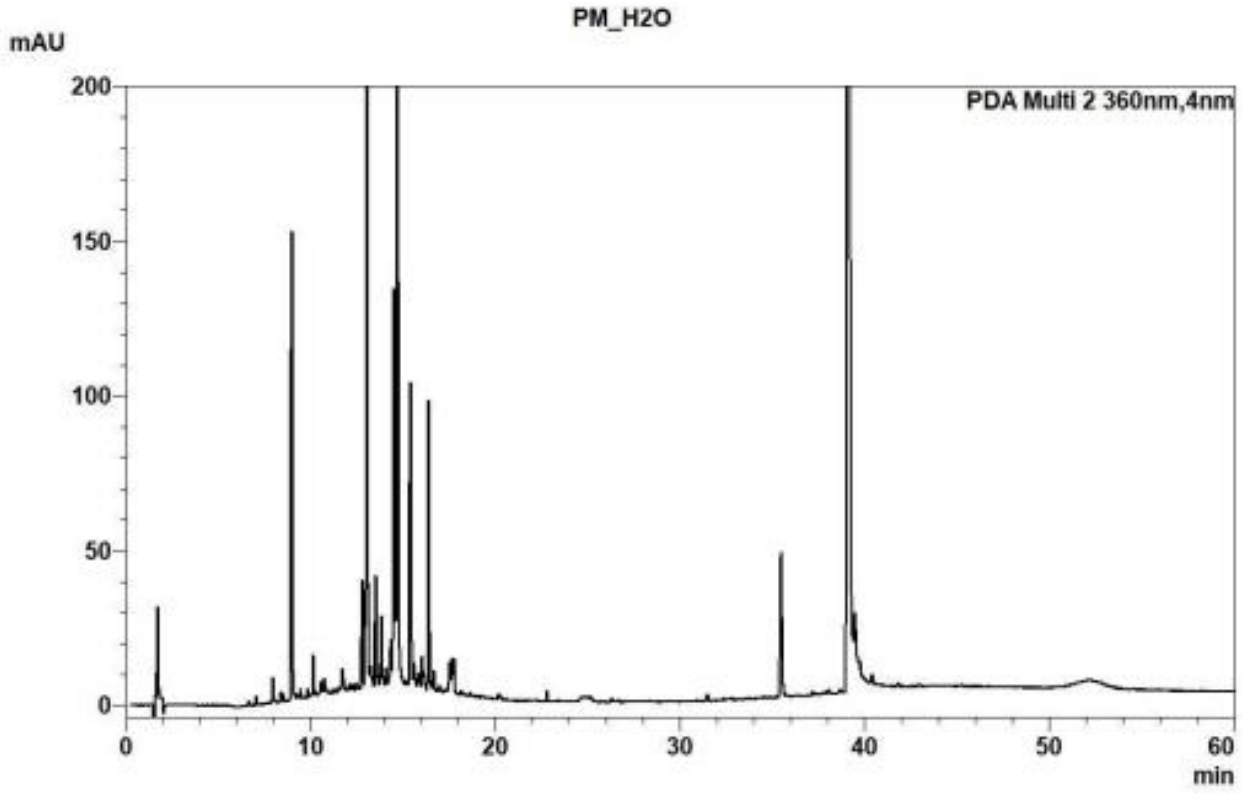
PM_H2O
mAU
PDA Multi 2 360nm,4nm
200
150
100
50
0
0
10
20
30
40
50
60
min

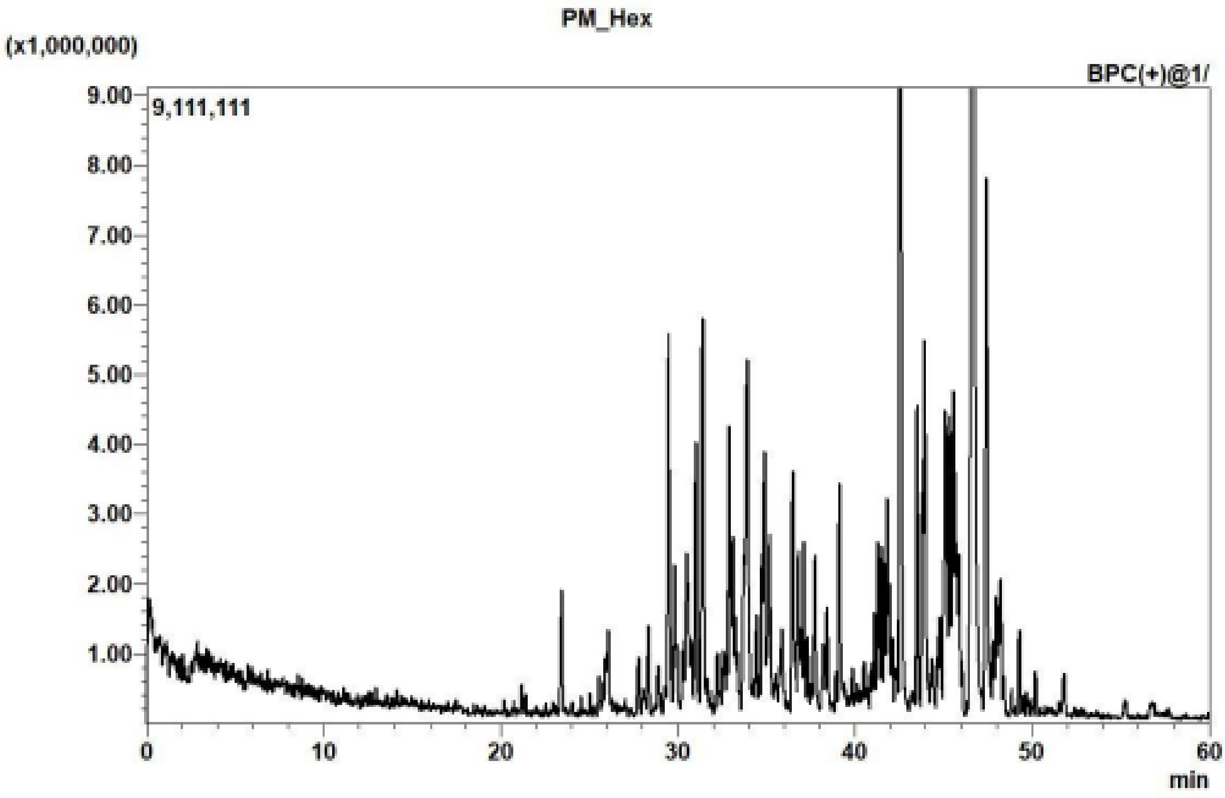
PM_Hex
(x1,000,000)
BPC(+)@1/
9,111,111
9.00
8.00
7.00
6.00
5.00
4.00
3.00
2.00
1.00
0
10
20
30
40
50
60
min

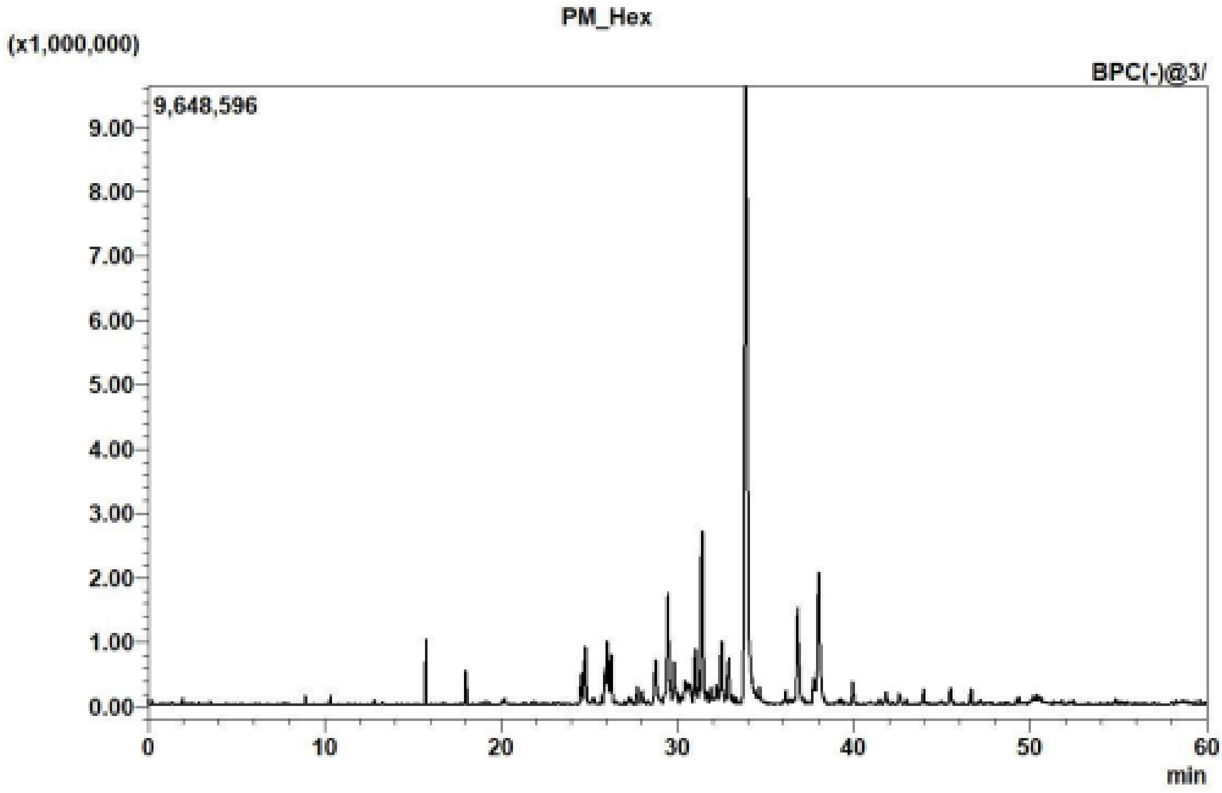
PM_Hex
(x1,000,000)
BPC(-)@3/
9,648,596
9.00
8.00
7.00
6.00
5.00
4.00
3.00
2.00
1.00
0.00
0
10
20
30
40
50
60
min

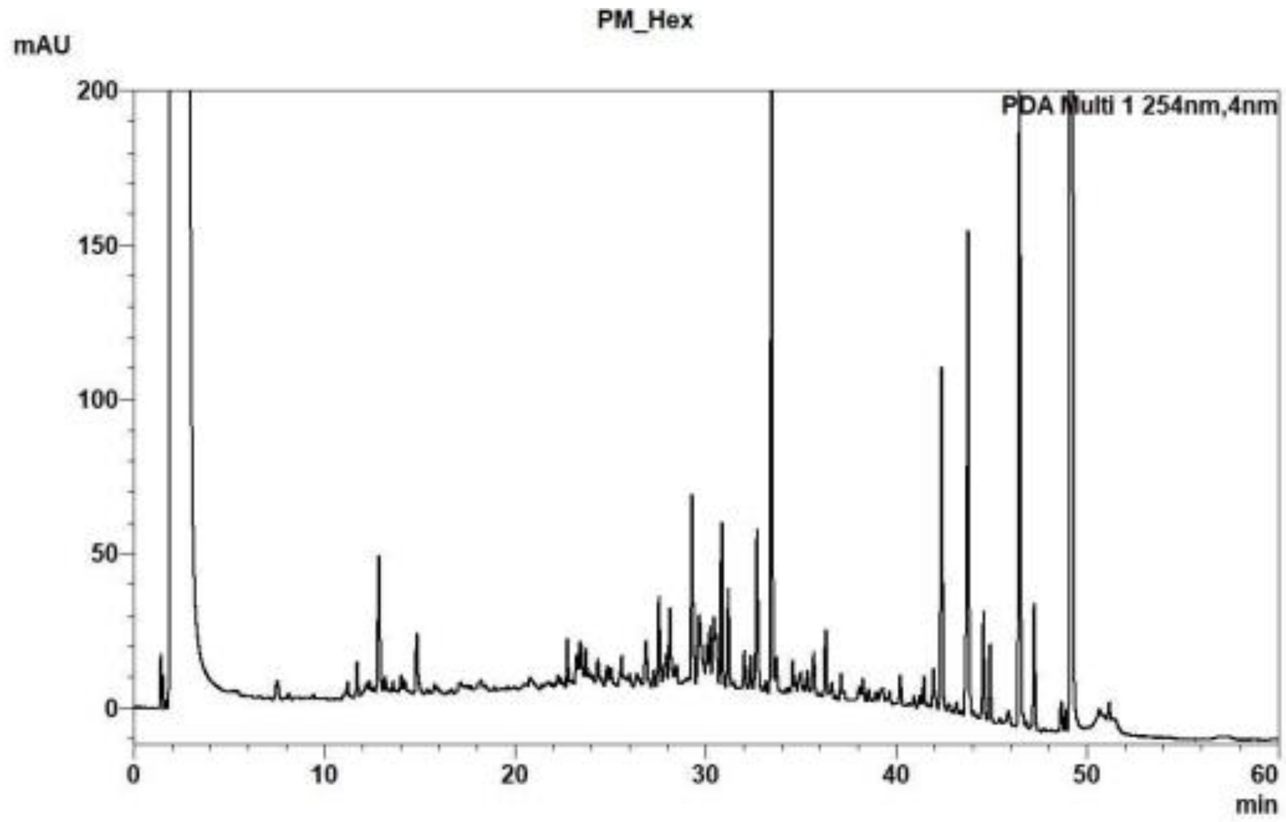
PM_Hex
mAU
PDA Multi 1 254nm,4nm
200
150
100
50
0
0
10
20
30
40
50
60
min

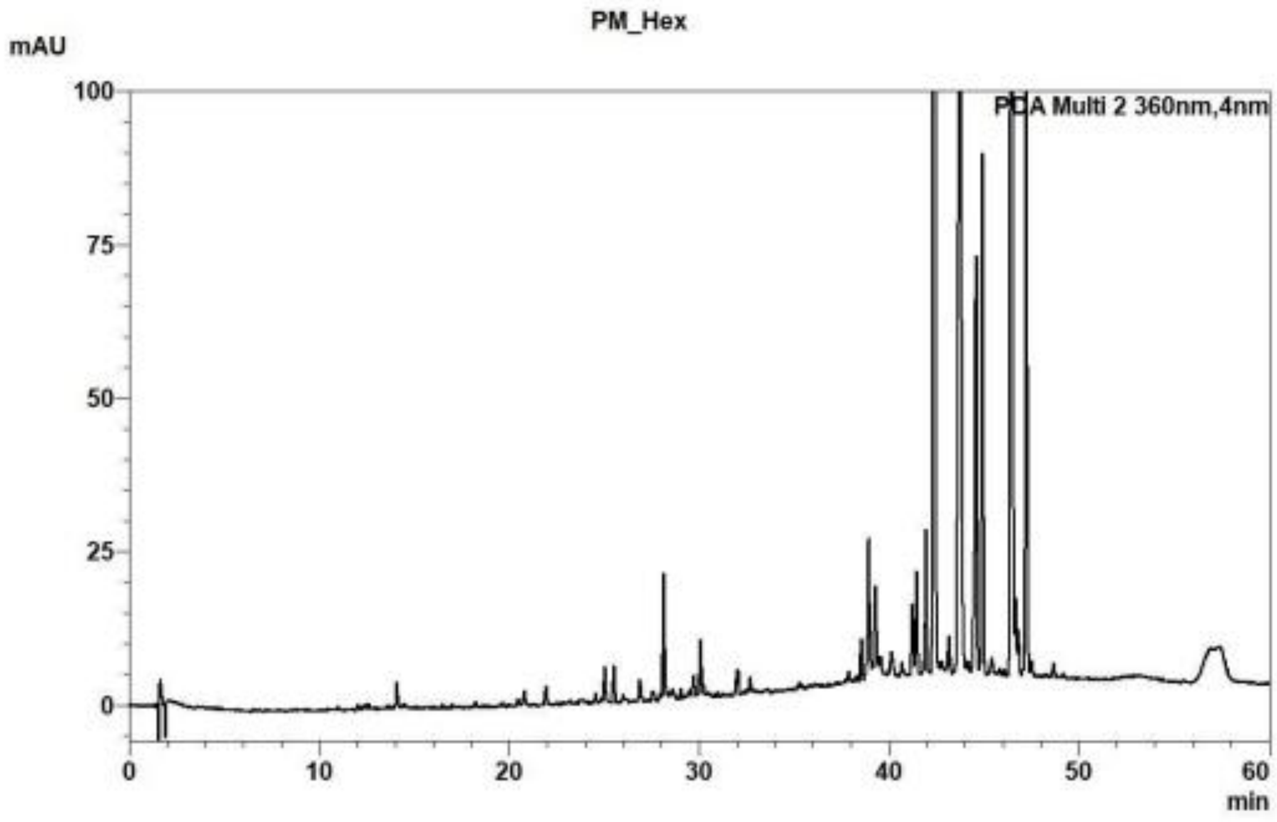
PM_Hex
mAU
PDA Multi 2 360nm,4nm
100
75
50
25
0
0
10
20
30
40
50
60
min

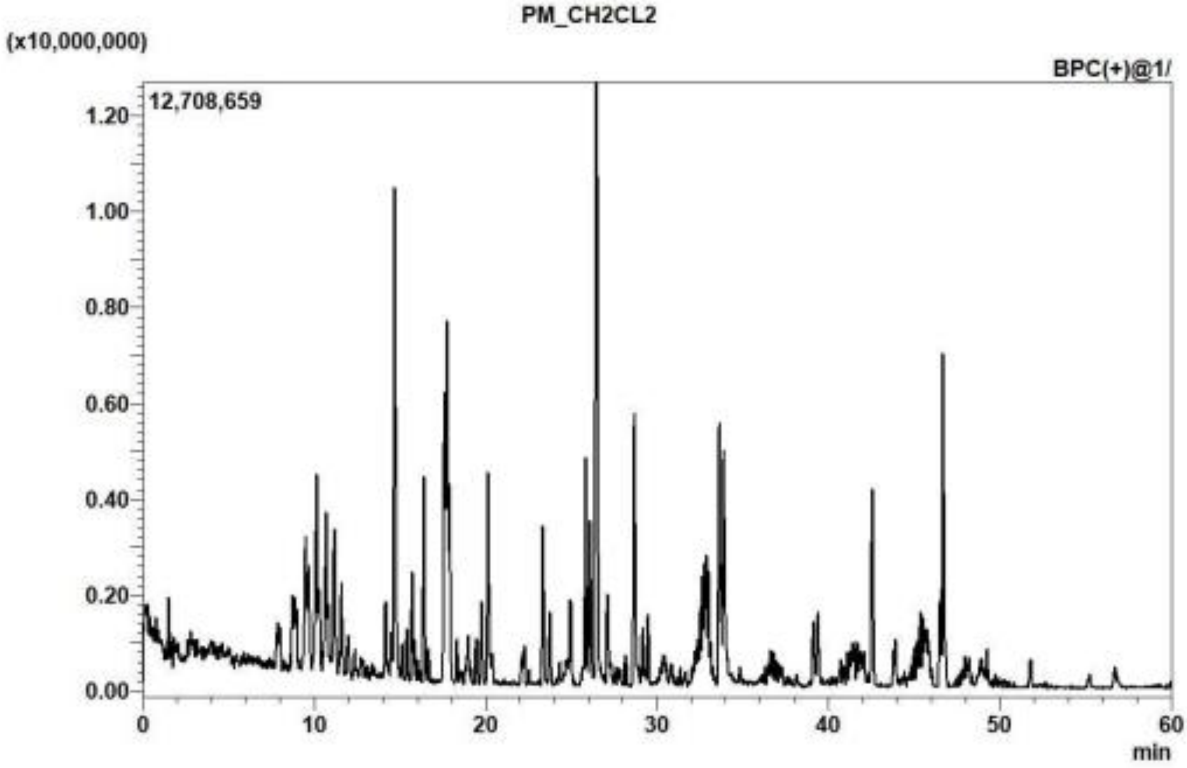
PM_CH2CL2
(x10,000,000)
BPC(+)@1/
12,708,659
1.20
1.00
0.80
0.60
0.40
0.20
0.00
0
10
20
30
40
50
60
min

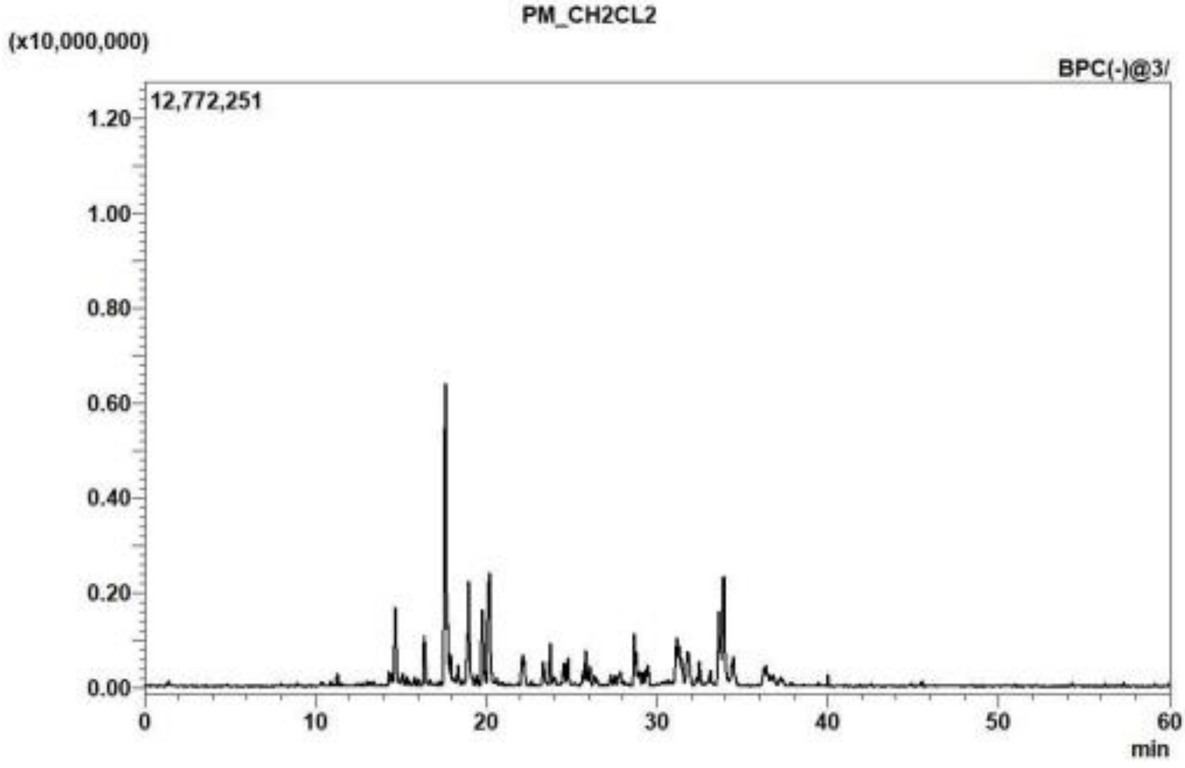
PM_CH2CL2
(x10,000,000)
BPC(-)@3/
12,772,251
1.20
1.00
0.80
0.60
0.40
0.20
0.00
0
10
20
30
40
50
60
min

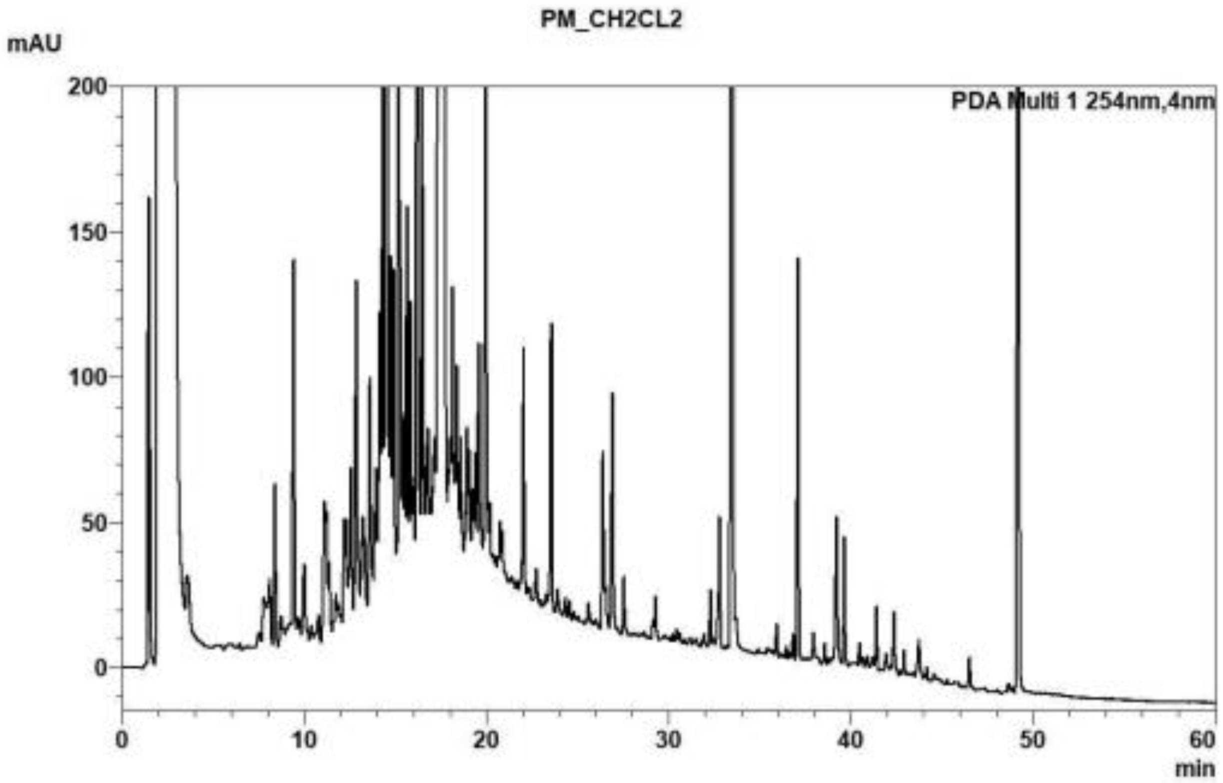
PM_CH2CL2
mAU
PDA Multi 1 254nm,4nm
200
150
100
50
0
0
10
20
30
40
50
60
min

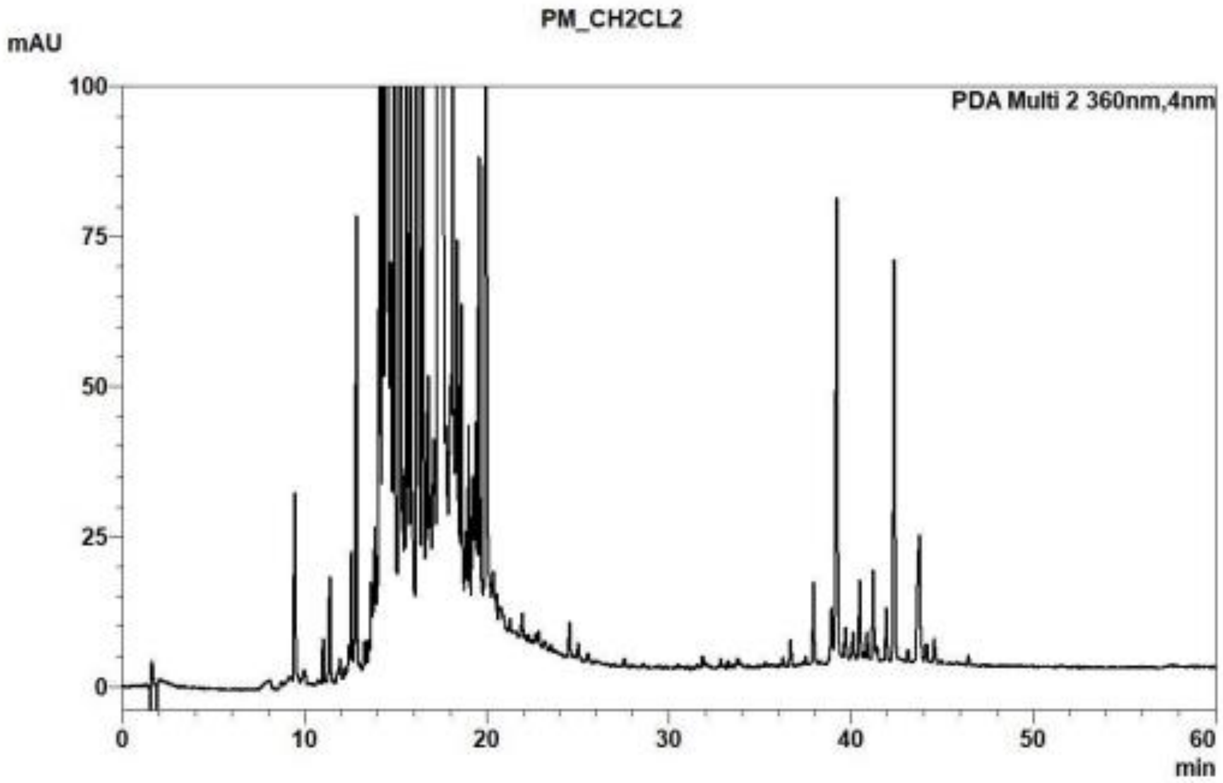
PM_CH2CL2
mAU
PDA Multi 2 360nm,4nm
100
75
50
25
0
0
10
20
30
40
50
60
min

Atriplex portulacoides

List of Figures

H_2O phase

Figure S19a. Base Peak Chromatogram (BPC) in the positive ionization mode of water extract of *Atriplex portulacoides*.

Figure S19b. Base Peak Chromatogram (BPC) in the negative ionization mode of water extract of *Atriplex portulacoides*.

Figure S19c. PDA chromatogram of water extracts of *Atriplex portulacoides* at 245 nm.

Figure S19d. PDA chromatogram of water extracts of *Atriplex portulacoides* at 360 nm.

Hexane extract

Figure S20a. Base Peak Chromatogram (BPC) in positive mode of *n*-hexane extract of *Atriplex portulacoides.*

Figure S20b. Base Peak Chromatogram (BPC) in negative mode of *n*-hexane extract of *Atriplex portulacoides.*

Figure S20c. PDA chromatogram of *n*-hexane extracts of *Atriplex portulacoides* at 245 nm.

Figure S20d. PDA chromatogram of *n*-hexane extracts of *Atriplex portulacoides* at 360 nm.

CH2Cl2 extract

Figure S21a. Base Peak Chromatogram (BPC) in positive mode of dichloromethane extract of *Atriplex portulacoides*.

Figure S21b. Base Peak Chromatogram (BPC) in negative mode of dichloromethane extract of *Atriplex portulacoides*.

Figure S21c. PDA chromatogram of dichloromethane extracts of *Atriplex portulacoides* at 245 nm.

Figure S21d. PDA chromatogram of dichloromethane of *Atriplex portulacoides* at 360 nm

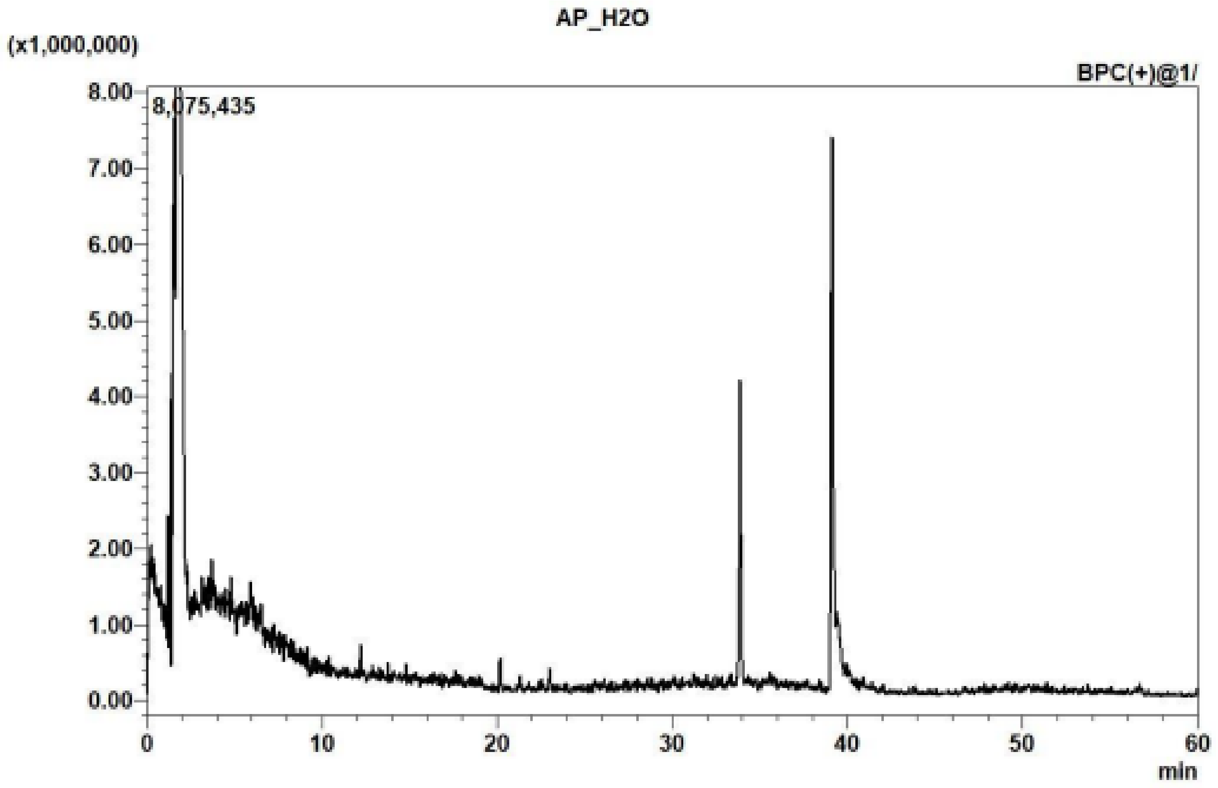
AP_H2O
(x1,000,000)
BPC(+)@1/
8,075,435
8.00
7.00
6.00
5.00
4.00
3.00
2.00
1.00
0.00
0
10
20
30
40
50
60
min

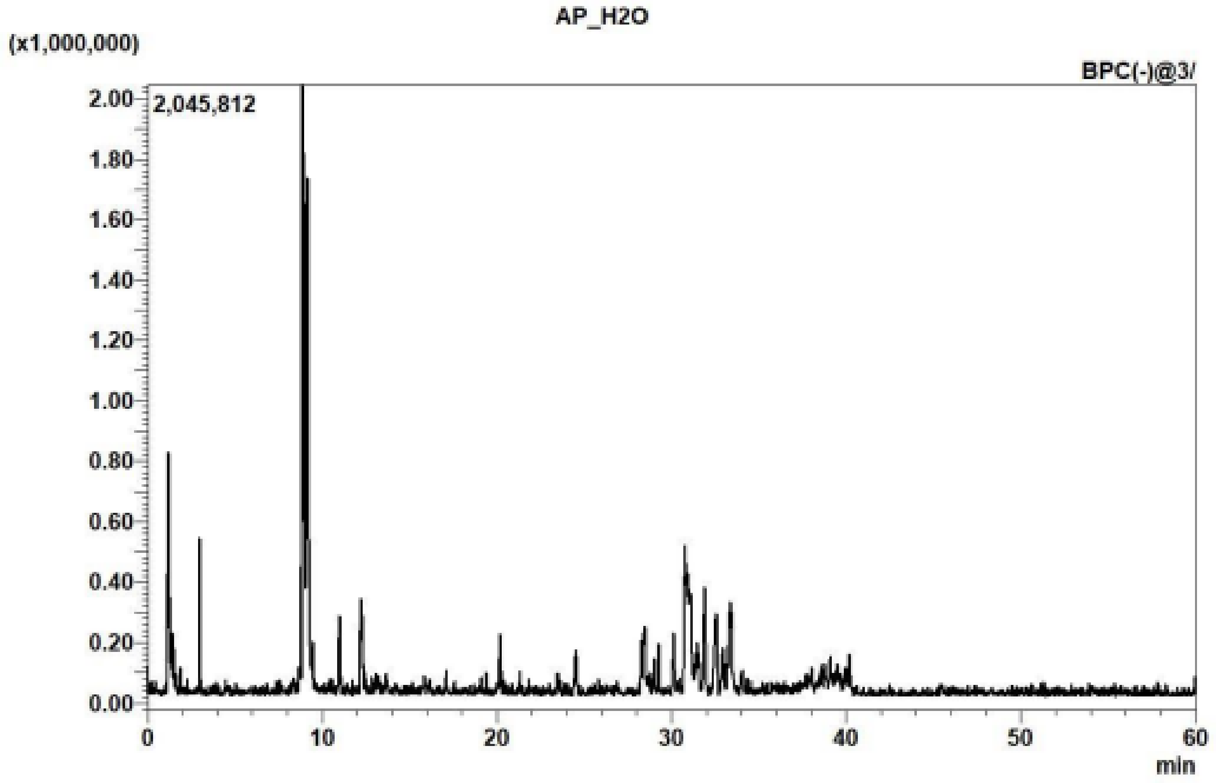
AP_H2O
(x1,000,000)
BPC(-)@3/
2,045,812
2.00
1.80
1.60
1.40
1.20
1.00
0.80
0.60
0.40
0.20
0.00
0
10
20
30
40
50
60
min

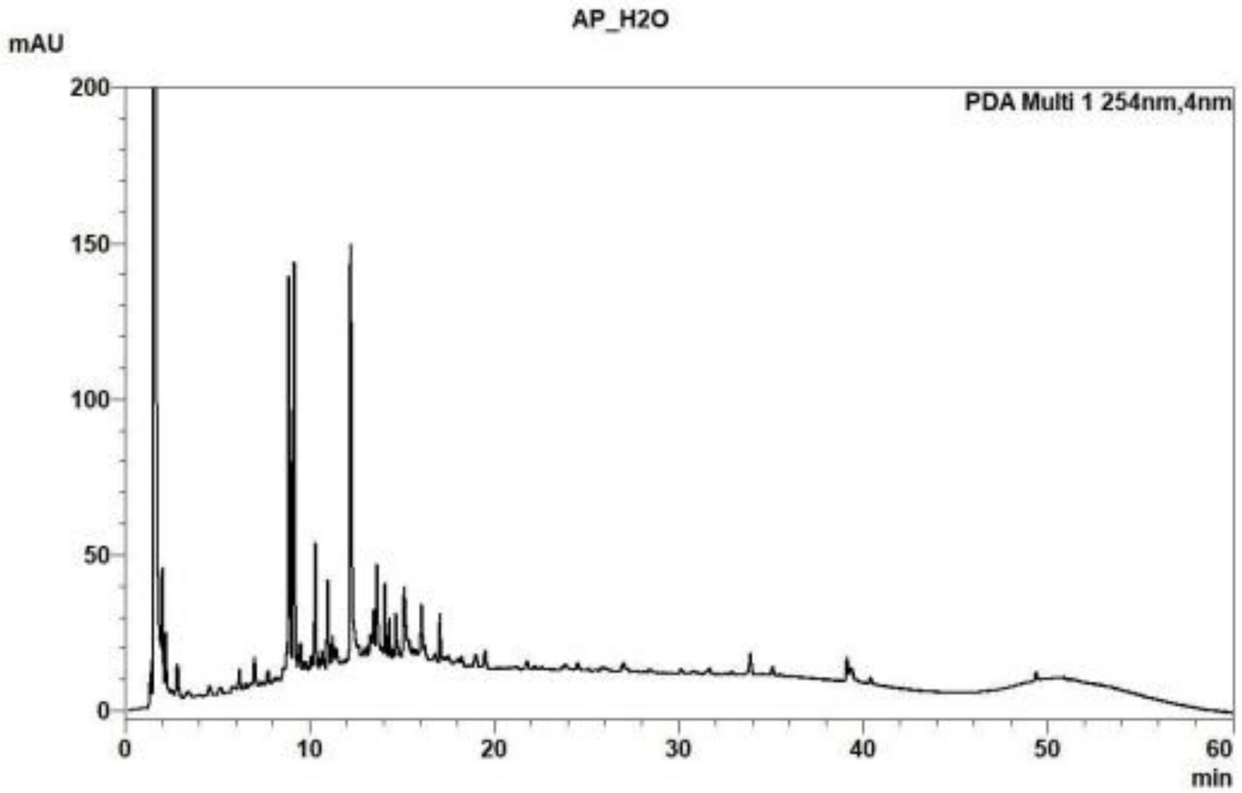
AP_H2O
mAU
PDA Multi 1 254nm,4nm
200
150
100
50
0
0
10
20
30
40
50
60
min

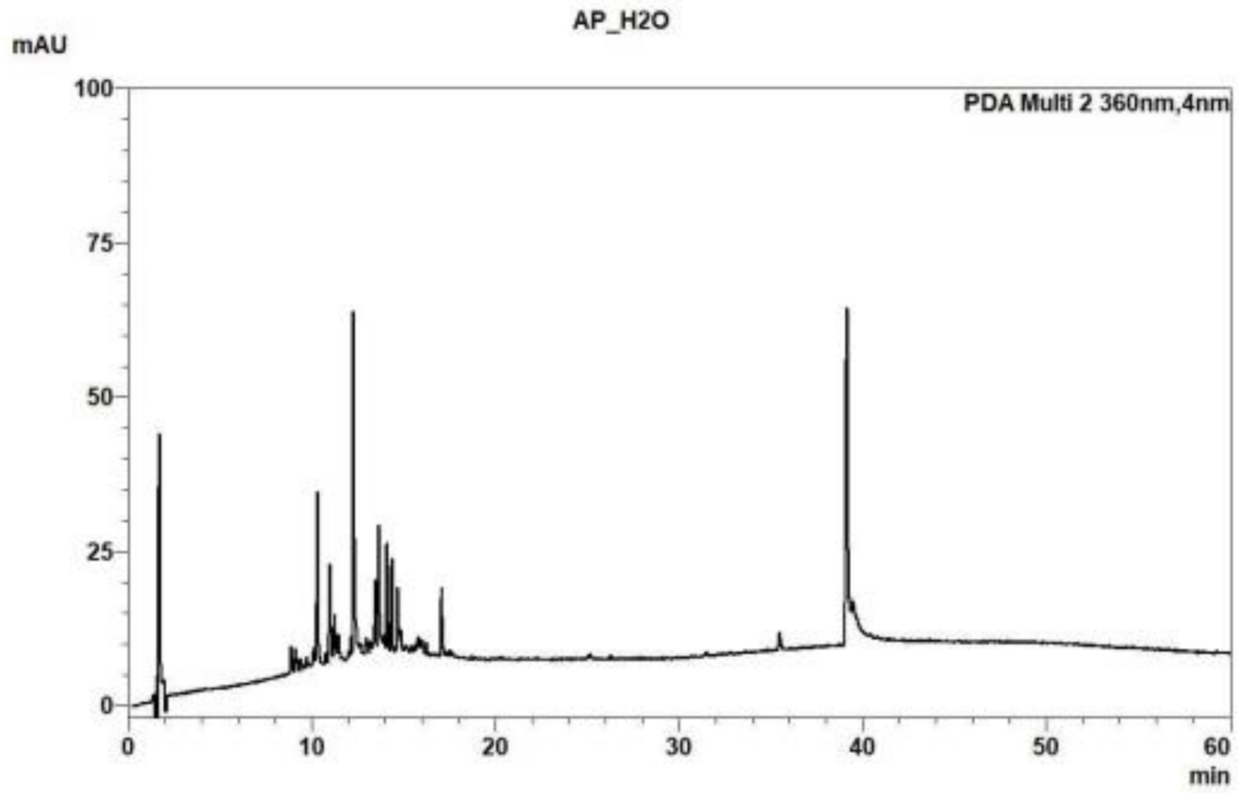
AP_H2O
mAU
PDA Multi 2 360nm,4nm
100
75
50
25
0
0
10
20
30
40
50
60
min

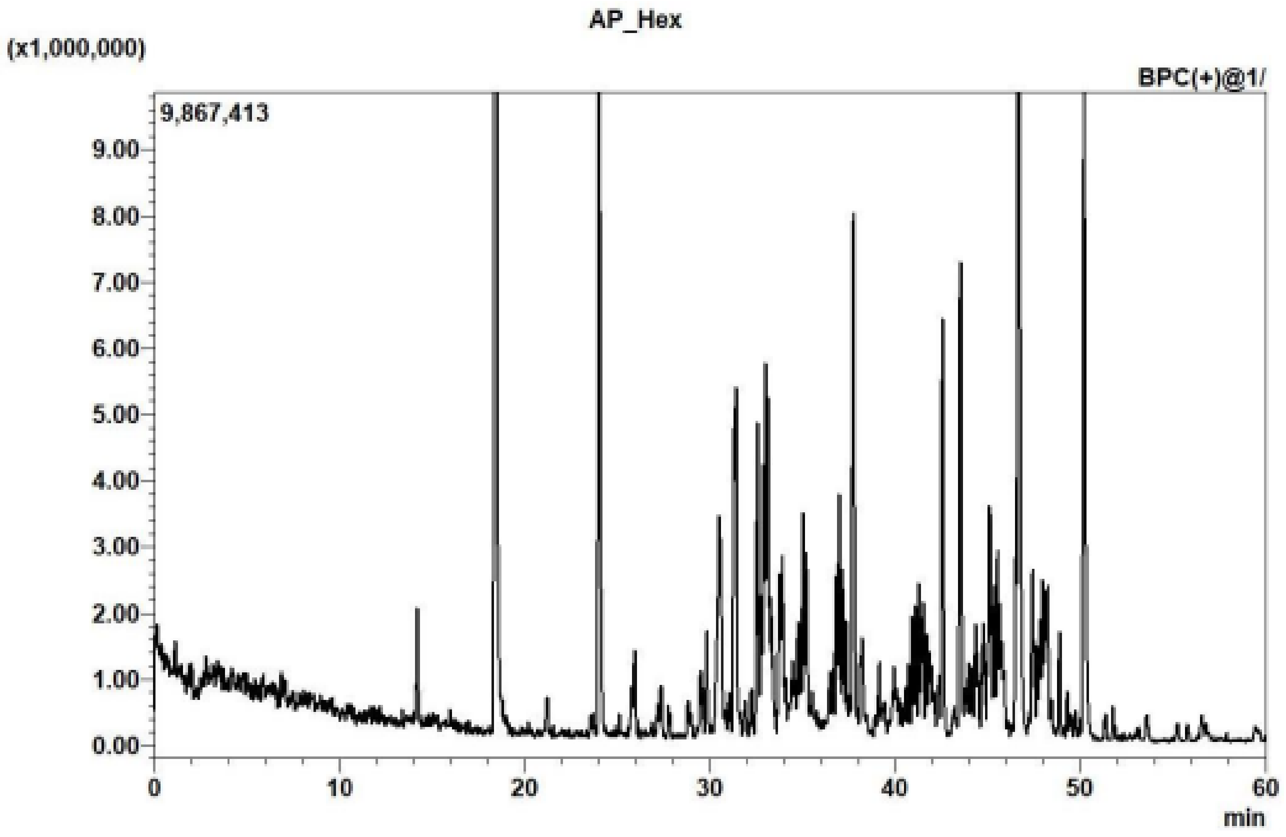
AP_Hex
(x1,000,000)
BPC(+)@1/
9,867,413
9.00
8.00
7.00
6.00
5.00
4.00
3.00
2.00
1.00
0.00
0
10
20
30
40
50
60
min

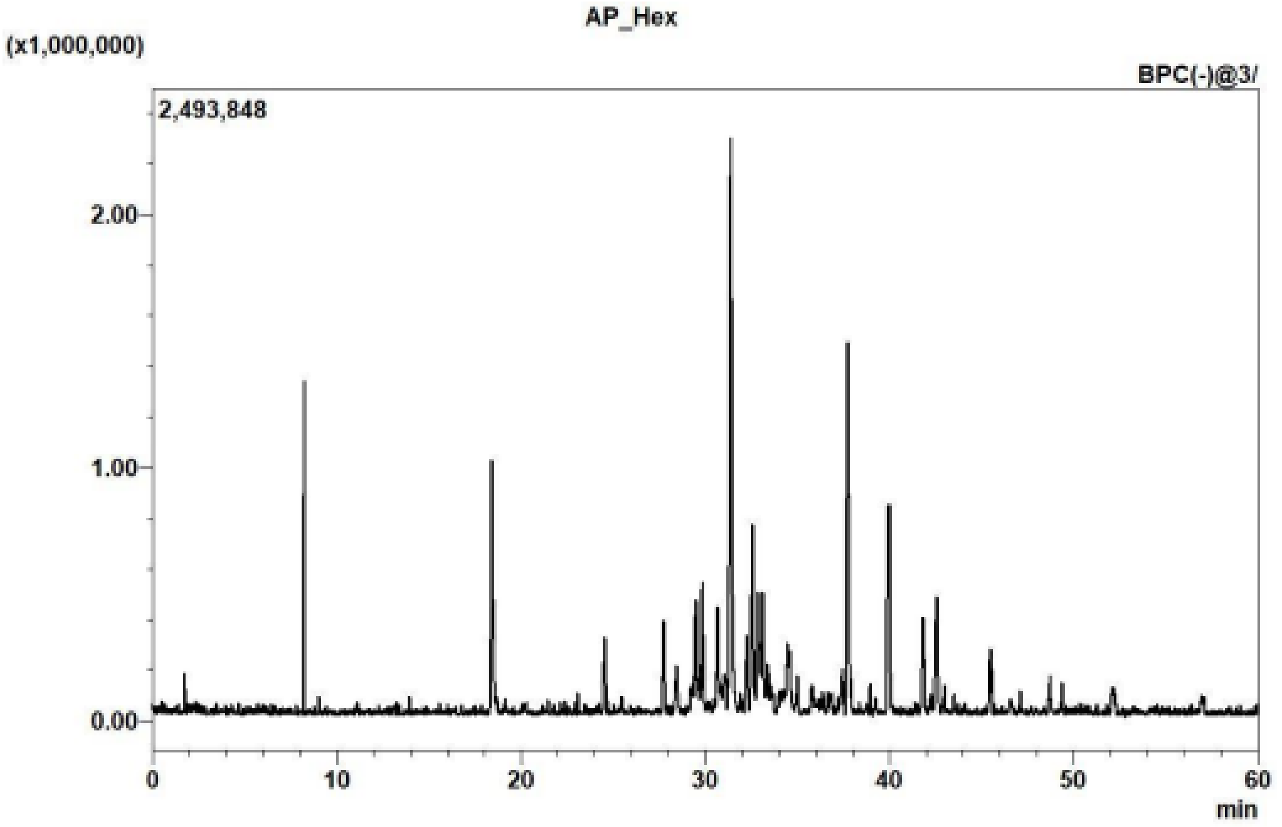
AP_Hex
(x1,000,000)
BPC(-)@3/
2,493,848
2.00
1.00
0.00
0
10
20
30
40
50
60
min

AP_Hex

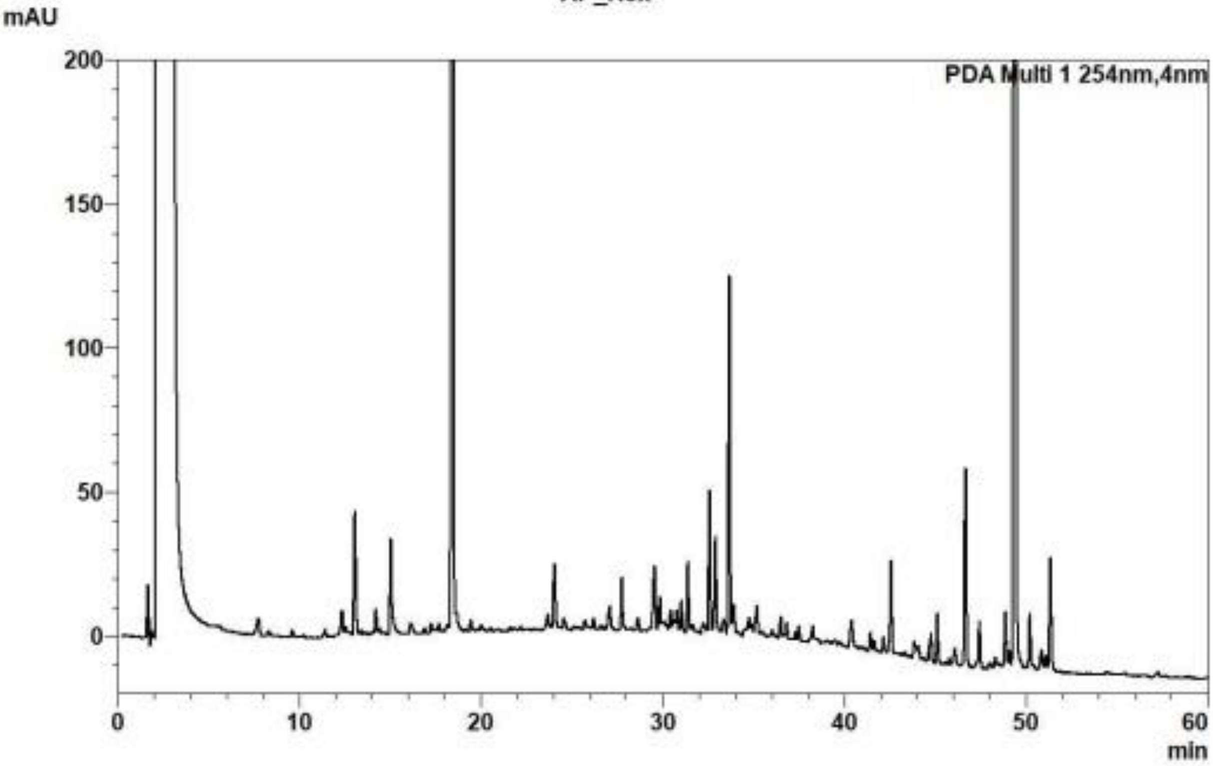
mAU
200
150
100
50
0
PDA Multi 1 254nm,4nm
0
10
20
30
40
50
60
min

AP_Hex

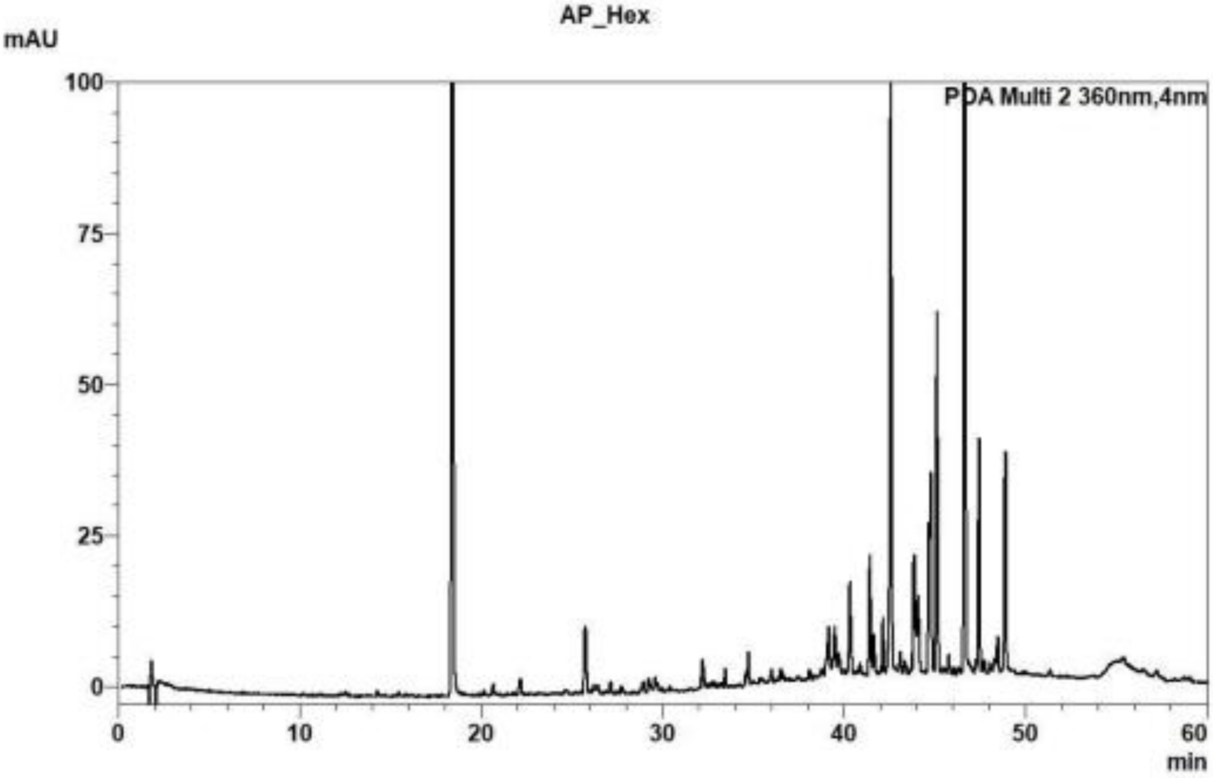
mAU
100
75
50
25
0
PDA Multi 2 360nm,4nm
0
10
20
30
40
50
60
min

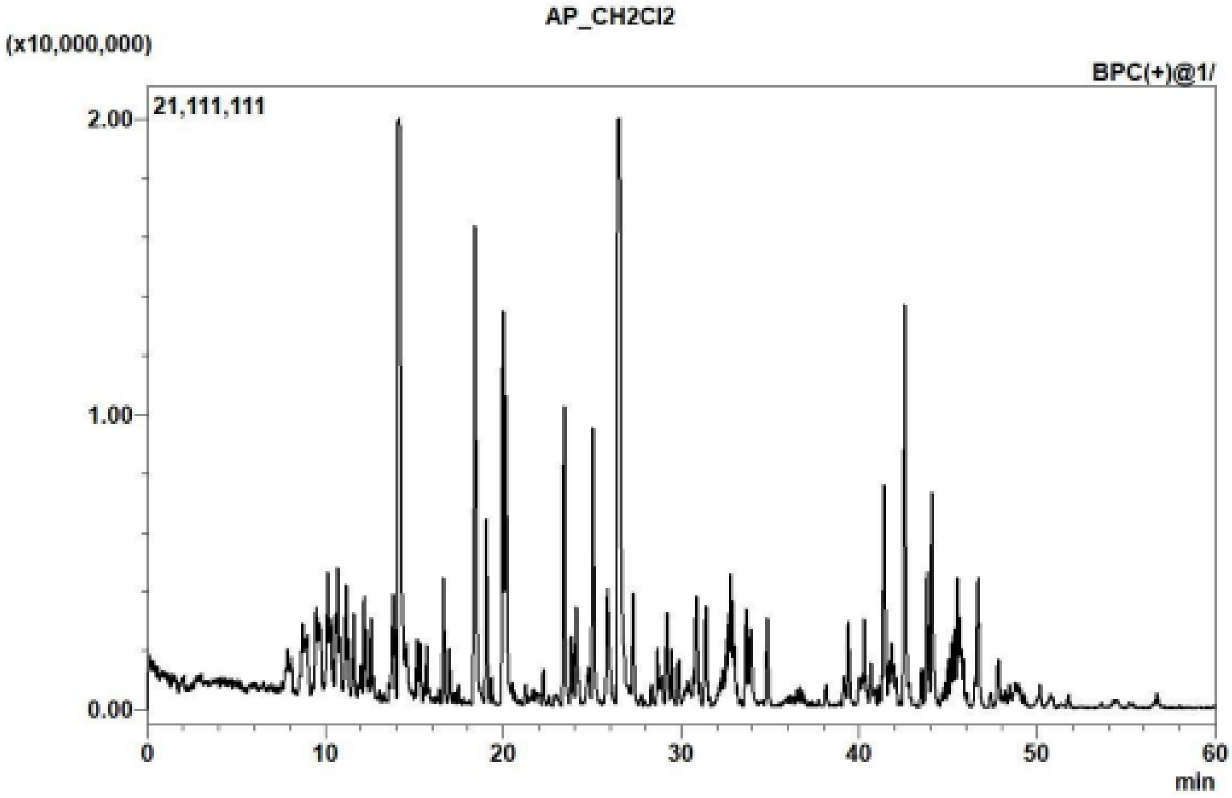
AP_CH2Cl2
(x10,000,000)
BPC(+)@1/
21,111,111
2.00
1.00
0.00
0
10
20
30
40
50
60
min

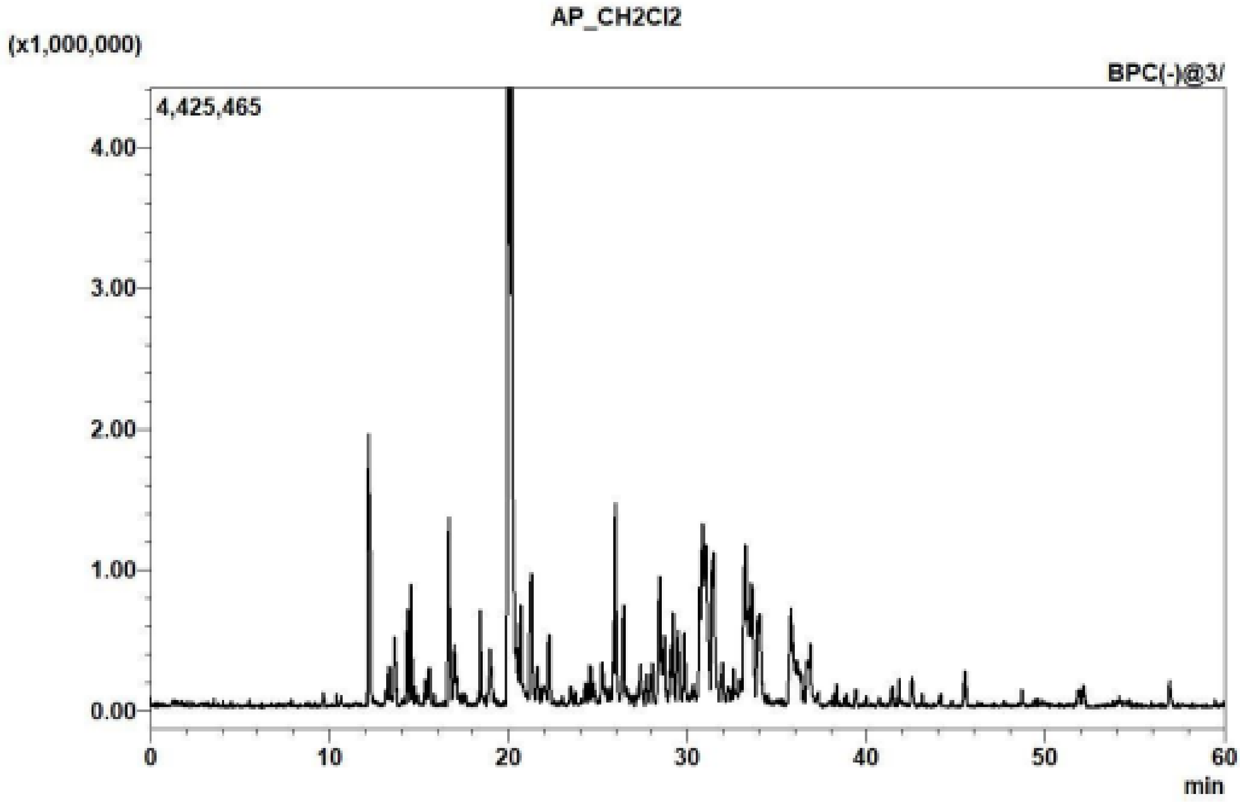
AP_CH2Cl2
(x1,000,000)
BPC(-)@3/
4,425,465
4.00
3.00
2.00
1.00
0.00
0
10
20
30
40
50
60
min

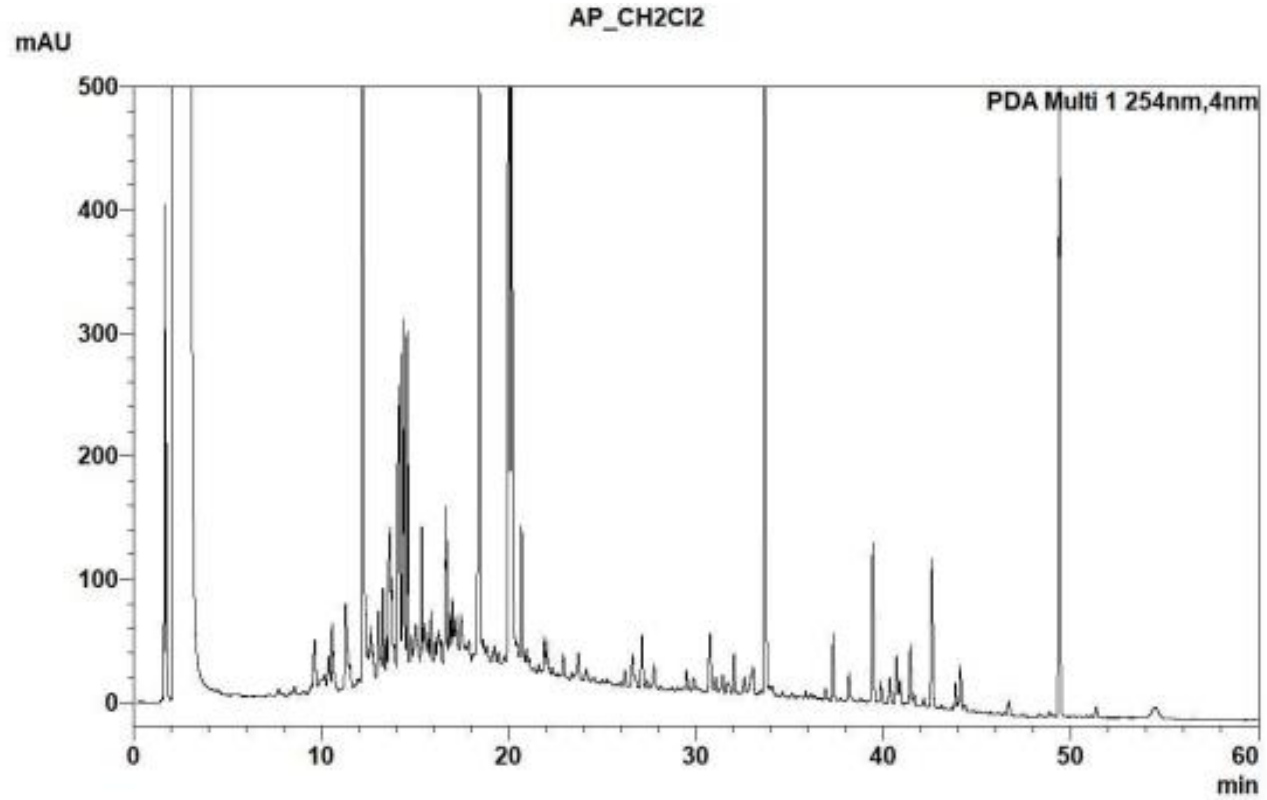
AP_CH2Cl2
mAU
PDA Multi 1 254nm,4nm
500
400
300
200
100
0
0
10
20
30
40
50
60
min

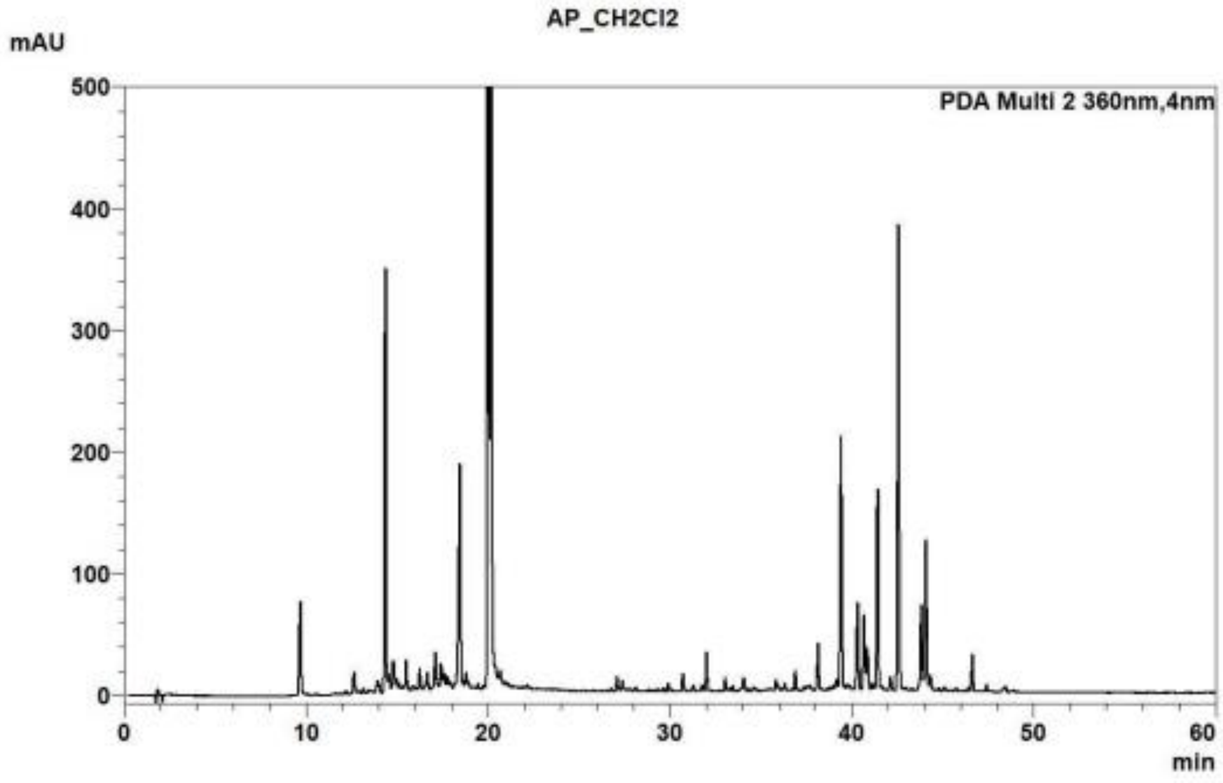
AP_CH2Cl2
mAU
PDA Multi 2 360nm,4nm
500
400
300
200
100
0
0
10
20
30
40
50
60
min
